# Glioblastoma at the crossroads: current understanding and future therapeutic horizons

**DOI:** 10.1038/s41392-025-02299-4

**Published:** 2025-07-09

**Authors:** Shilpi Singh, Devanjan Dey, Debashis Barik, Iteeshree Mohapatra, Stefan Kim, Mayur Sharma, Sujata Prasad, Peize Wang, Amar Singh, Gatikrushna Singh

**Affiliations:** 1 Department of Neurosurgery, University of Minnesota, Minneapolis, MN USA; 2 Schulze Diabetes Institute, Department of Surgery, University of Minnesota, Minneapolis, MN USA; 3 Center for Computational Natural Science and Bioinformatics, International Institute of Information Technology, Hyderabad, Telangana India; 4 Department of Veterinary and Biomedical Sciences, University of Minnesota, Saint Paul, MN USA; 5 MLM Medical Labs LLC, Oakdale, MN USA

**Keywords:** Drug development, Prognostic markers

## Abstract

Glioblastoma (GBM) remains the most aggressive and lethal brain tumor in adults and poses significant challenges to patient survival. This review provides a comprehensive exploration of the molecular and genetic landscape of GBM, focusing on key oncogenic drivers, such as epidermal growth factor receptor (EGFR), platelet-derived growth factor receptor (PDGFR), and the PI3K/AKT/mTOR pathway, which are critical for tumorigenesis and progression. We delve into the role of epigenetic alterations, including DNA methylation and histone modifications, in driving therapy resistance and tumor evolution. The tumor microenvironment is known for its pivotal role in immune evasion, with tumor-associated macrophages, myeloid-derived suppressor cells, and regulatory T cells creating an immunosuppressive niche that sustains GBM growth. Emerging therapies, such as immunotherapies, oncolytic viral therapies, extracellular vesicle-based approaches, and non-coding RNA interventions, are highlighted as promising avenues to disrupt GBM pathogenesis. Advances in precision medicine and innovative technologies, including electric field therapy and locoregional treatments, are discussed for their potential to overcome the blood‒brain barrier and treatment resistance. Additionally, this review underscores the importance of metabolic reprogramming, particularly hypoxia-driven adaptations and altered lipid metabolism, in fueling GBM progression and influencing the therapeutic response. The role of glioma stem cells in tumor recurrence and resistance is also emphasized, highlighting the need for targeted therapeutic approaches. By integrating molecular targeting, immune energetics, and technological advancements, this review outlines a multidisciplinary framework for improving GBM treatment outcomes. Ultimately, the convergence of genetic, metabolic, and immune-based strategies offers transformative potential in GBM management, paving the way for increased patient survival and quality of life.

## Introduction

Glioblastoma (GBM) is the most prevalent and aggressive malignant brain tumor in adults and presents a formidable challenge in oncology due to its rapid progression, therapeutic resistance, and poor prognosis. Despite extensive research, the median survival remains dismal at 12–15 months.^1^ The latest classification of central nervous system (CNS) tumors categorizes gliomas into a diverse group of glial-derived brain tumors, with GBM being the most aggressive grade IV subtype, characterized by an isocitrate dehydrogenase (IDH) wild-type status. GBM is further distinguished by key molecular alterations, including epidermal growth factor receptor (EGFR) amplification, telomerase reverse transcriptase (TERT) promoter mutations, and distinct chromosomal abnormalities.^2,3^ These features contribute to the highly invasive nature and resistance of tumors to conventional therapies. In contrast, IDH mutant gliomas, which are commonly found in lower-grade gliomas and secondary GBMs, exhibit distinct epigenetic landscapes and are associated with better clinical outcomes. These tumors exhibit the glioma-CpG island methylator phenotype (G-CIMP),^4,5^ influencing tumor behavior and therapeutic response, highlighting the importance of epigenetic regulation in gliomagenesis. Additional genetic alterations, such as mutations in the alpha-thalassemia mental retardation X-linked (*ATRX*) gene and DNA methylation profiles, further refine tumor classification and influence treatment strategies.^6,7^

A major obstacle in GBM treatment is its cellular and molecular heterogeneity, comprising differentiated tumor cells, glioma stem-like cells (GSCs), and a dynamic tumor microenvironment (TME). Advanced sequencing technologies have identified diverse GBM subtypes and cellular states, emphasizing the need for therapeutic strategies targeting both molecular drivers and the TME. GSCs, in particular, play pivotal roles in tumor progression, therapeutic resistance, and recurrence due to their self-renewal capabilities and adaptability.^8,9^ However, their resilience poses a major barrier to effective treatment. Additionally, genomic instability and oncogenic signaling pathways, such as the EGFRvIII-driven dysregulation of the receptor tyrosine kinase/mitogen-activated protein kinase (RTK/RAS/MAPK) pathway, fuel aggressive tumor behavior. The frequently altered phosphoinositide-3 kinase/protein kinase B/mammalian target of rapamycin (PI3K/AKT/mTOR) axis, which regulates tumor growth and survival, is a promising therapeutic target, although clinical trials of mTOR inhibitors have shown limited success.^10^ A comprehensive understanding of the interplay between molecular alterations, GSC biology, and the TME is essential for developing innovative, more effective treatment strategies.

The TME significantly contributes to tumor progression by fostering tumor growth, immune evasion, and resistance to therapy.^11^ Interactions among tumor-associated macrophages (TAMs), neutrophils, myeloid-derived suppressor cells (MDSCs), and T cells within the TME create an immunosuppressive niche that enables tumor survival and proliferation. In recurrent GBMs, these dynamics intensify, with increased immune cell infiltration and the upregulation of checkpoint proteins such as programmed death-ligand 1 (PD-L1) and PD-1, underscoring the importance of precision immunotherapy to improve outcomes.^12^ In addition to cellular components, extracellular vesicles (EVs), microRNAs (miRNAs), and long non-coding RNAs (lncRNAs) have emerged as both molecular biomarkers and therapeutic targets in GBM.^13^ For example, miRNA-21, which is frequently upregulated in GBM, is correlated with poor survival and higher tumor grades, whereas other miRNAs, such as miR-128 and miR-342-3p, exhibit therapy-induced expression changes and are linked to glioma grade.^14^ Similarly, circulating lncRNAs and circular RNAs (circRNAs) have shown potential for predicting patient outcomes,^15^ further emphasizing their value in GBM treatment strategies. Targeting the TME and integrating molecular markers into therapeutic approaches represent crucial steps toward enhancing treatment efficacy. By addressing these intricate interactions and leveraging molecular insights, GBM management can progress toward more personalized and effective strategies.

**Fig. 1:**
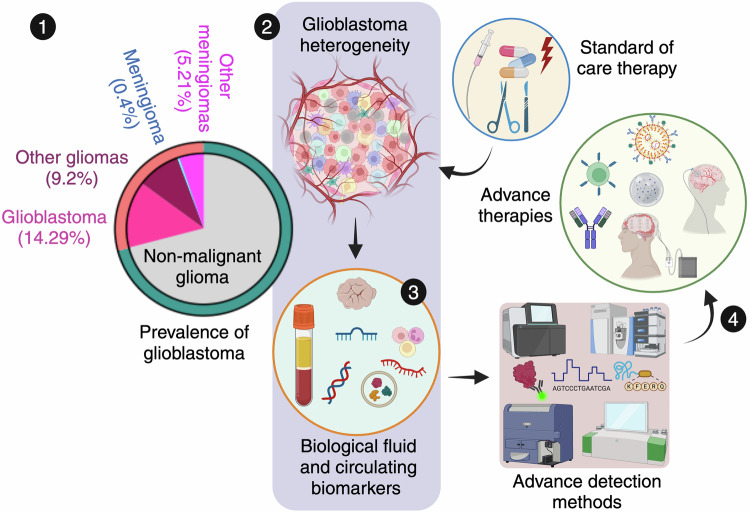
Glioblastoma landscape and path towards targeted therapies. **1.** The pie chart illustrates glioma trends, with a focus on glioblastoma (GBM) prevalence in the United States. Data source: Cancer Stat Facts: Brain and other nervous system cancers identified by the National Cancer Institute’s Surveillance, Epidemiology, and End Results Program, 2014–2020. **2.** GBM, marked by its pronounced molecular, genetic, and cellular heterogeneity, presents substantial obstacles for accurate diagnosis and effective treatment. **3.** Advanced diagnostic methods, leveraging biofluid biomarkers such as liquid biopsies and circulating biomolecules, alongside high-definition detection technologies, are crucial for precise detection. **4.** These innovations are driving the development of targeted and more effective therapies for GBM treatment

This review delves into the cellular heterogeneity of GBM, emphasizing the genetic, epigenetic and oncogenic signaling pathways that drive tumor progression, therapy resistance and recurrence. This highlights the crucial role of GSCs in tumor persistence, as well as the impact of the TME in fostering immune evasion and therapeutic resistance. Additionally, key molecular alterations, including EGFR amplification, IDH mutations, O^6^-methylguanine-DNA methyltransferase (MGMT) modifications, histone epigenetic changes and signaling pathway dysregulation, are being examined for their contributions to the aggressive behavior and treatment challenges of GBM. This review critically evaluates current and emerging therapeutic strategies, including locoregional treatments, systemic chemotherapy, and combination therapies, alongside innovative approaches such as oncolytic viral therapy, EV-based therapies, non-coding RNA (ncRNA) interventions, electric field therapy, and precision medicine advancements (Fig. 1). These approaches are discussed for their potential to overcome existing limitations, such as therapeutic resistance, tumor recurrence, immune adaptation, metabolic reprogramming and blood‒brain barrier (BBB) delivery challenges. By addressing these persistent hurdles and highlighting promising research directions, this review aims to inspire innovative strategies that could transform GBM treatment, improve patient outcomes, and advance the therapeutic landscape for this devastating disease.

## Molecular characterization of GBM and diagnostic biomarkers

### Clinical grading of GBM

GBM is classified as a World Health Organization (WHO) grade IV glioma, distinguished by aggressive behavior, high recurrence rates, and resistance to conventional therapies. Its histopathological hallmarks include nuclear atypia, cellular pleomorphism, mitotic activity, microvascular proliferation, and necrosis. In addition to these defining features, several histologic variants, such as gliosarcomas, giant-cell GBM, small-cell GBM, and epithelioid GBM, present distinct molecular and clinical implications. Notably, epithelioid GBM is characterized by v-raf murine sarcoma viral oncogene homolog B1 (BRAF) V600E mutations,^16^ highlighting the genetic heterogeneity within GBM. GBM falls within the diffuse glioma category, which presents significant treatment challenges because of its highly infiltrative nature. Unlike circumscribed gliomas, which have well-defined margins and generally better prognosis, diffuse gliomas are characterized by extensive invasion into normal brain tissue, limiting the effectiveness of surgical resection.^17^ As the most aggressive form of diffuse glioma, GBM accounts for nearly 50% of all primary malignant brain tumors and represents the most lethal intrinsic brain tumor.^18^

The evolution of molecular classification has refined GBM subtyping, moving beyond histological grading to a deeper understanding of its genetic and epigenetic landscape (Table S1). The classification system proposed by Phillips et al. divides GBM into three subtypes with distinct prognostic and therapeutic implications. 1) Proneural GBM, which is predominantly observed in younger patients, is associated with lower pathological severity and relatively better survival outcomes. It is characterized by neural-like gene expression patterns, including those of the neural cell adhesion molecules GABR1 and SNAP91, which resemble those of normal brain tissue. 2) Proliferative GBM is associated with high levels of cellular proliferation, with significant upregulation of the expression of markers such as TOP2A and PCNA, indicating a more aggressive tumor biology. 3) Mesenchymal GBM is the most invasive subtype and is characterized by the overexpression of angiogenesis markers (e.g., vascular endothelial growth factor {VEGF}, PECAM1), the loss of phosphatase and tensin homolog (PTEN) and neurofibromin 1 (NF1), and the activation of PI3K/AKT signaling, which are correlated with a poor prognosis.^19^

**Fig. 2:**
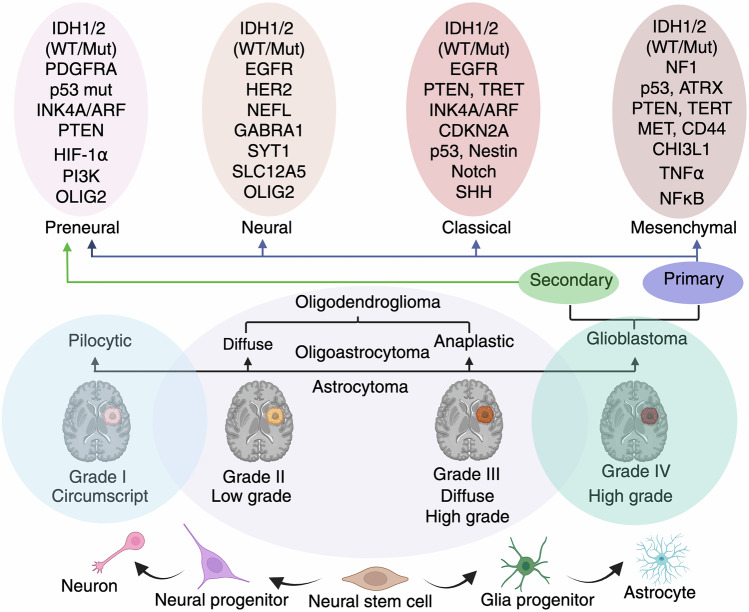
Clinical and molecular grading of gliomas. Schematic representation of the molecular classification and histopathological grading of gliomas, along with their cellular origins and progression. The bottom panel shows a developmental lineage from neural stem cells to neurons, astrocytes, and glial progenitors. Pilocytic astrocytomas (Grade I) are typically circumscribed and low grade, whereas diffuse astrocytomas (Grade II), anaplastic astrocytomas (Grade III), and glioblastomas (Grade IV) represent progressive stages of malignancy and infiltrative behavior. The top panel highlights the molecular subtypes of glioblastoma: proneural, neural, classical, and mesenchymal, each defined by distinct genetic alterations such as IDH1/2, EGFR, p53, PTEN, NF1, and others. These subtypes correlate with the primary (de novo) or secondary (progression from lower-grade gliomas) origins of glioblastoma. This classification underscores the integration of molecular and clinical parameters for diagnosis, prognosis, and therapeutic decision-making in gliomas

Verhaak et al. further expanded the classification into four subtypes: proneural, neural, classical, and mesenchymal. While proneural GBM is enriched in platelet-derived growth factor receptor alpha (PDGFR-α) expression and IDH1 mutations, which confer a potential survival advantage, it remains resistant to conventional therapy. Neural subtypes, which share gene expression similarities (SYT1, GABRA1 and NEFL) with normal neurons, exhibit enhanced sensitivity to radiation and chemotherapy.^20^ In contrast, the classical subtype is characterized by EGFR amplification, RB pathway alterations, chromosome 7 amplification, chromosome 10 loss, and high activation of the sonic hedgehog (SMO, GAS1, GLI2) and Notch signaling (NOTCH3, JAG1, LFNG) pathways, making it more responsive to aggressive treatment. Mesenchymal GBM, characterized by extensive necrosis, inflammatory markers, the upregulation of interstitial and angiogenesis genes, frequent deletions of the tumor suppressor genes tumor protein 53 (p53), PTEN and NF1 and highly expressed genes such as VEGF-A, VEGF-B, ANG1, and ANG2, represents the most aggressive subtype with limited treatment success^21^ (Fig. 2).

In addition to transcriptomic profiling, DNA methylation-based classification provides further granularity in GBM subtyping. Large-scale sequencing studies have identified six methylation clusters (M1–M6), each with distinct prognostic and biological implications. Among them, the G-CIMP subtype (cluster M5) is characterized by hypermethylation and frequent IDH1 mutations, which are correlated with improved survival outcomes and a less aggressive clinical course. In contrast, Cluster M6, characterized by relative hypomethylation and a predominance of IDH1 wild-type tumors, represents a more aggressive phenotype with a poorer prognosis. Further molecular refinement revealed the enrichment of missense mutations and deletions in histone-lysine N-methyltransferase 2A (*KMT2A*) or *MLL* and histone deacetylase (*HDAC*) family genes within Cluster M2, underscoring the role of chromatin remodeling in GBM pathogenesis.^22^ Additionally, Cluster 3 has a greater frequency of p53 mutations, along with IDH1 wild-type and 1p/19q deletions, further distinguishing high-risk subgroups with aggressive tumor behavior.^23^

The integration of DNA methylation patterns with genetic alterations offers a comprehensive framework for patient stratification, refining prognostic predictions and informing therapeutic decision making. These molecular subtypes not only highlight the heterogeneity of GBM but also provide potential targets for precision medicine. Future research should focus on unraveling the regulatory mechanisms driving these epigenetic changes, which is essential for overcoming the inherent therapeutic resistance of GBM and improving patient outcomes.

### Diagnostic biomarkers

#### IDH mutation

IDH mutations are pivotal in glioma classification and influence tumor metabolism, epigenetic regulation, redox balance, DNA repair and cellular differentiation. These mutations, which primarily affect IDH1 (R132), which is localized in the cytosol and peroxisomes, and IDH2 (R172), which is located in the mitochondria, lead to the accumulation of D-2-hydroxyglutarate metabolites, driving oncogenesis through widespread epigenetic dysregulation.^24^ These mutations serve as key molecular discriminators between glioma subtypes and are highly prevalent in lower-grade diffuse gliomas (WHO grades II–III) and secondary GBMs^25^ but are largely absent in primary GBMs, which are predominantly IDH wild-type.^26^ This distinction has led to the integration of the IDH status into the WHO glioma classification.

IDH mutations are correlated with improved survival and treatment response, distinguishing IDH-mutant gliomas from their more aggressive IDH wild-type counterparts. Large-scale analysis confirmed a high prevalence of IDH mutations in oligodendrogliomas (71%) and diffuse astrocytomas (58.6%), with a decreasing frequency in anaplastic astrocytomas (27.6%) and GBMs (10.4%).^27^ These patterns reinforce IDH mutation status as a key factor in glioma stratification and prognosis. Patients with Grade III gliomas lacking 1p/19q codeletion and harboring IDH mutations have significantly prolonged progression-free survival (PFS) and overall survival (OS), with similar trends observed in secondary high-grade gliomas (HGGs).^28^ A comprehensive meta-analysis further validated the strong correlation between IDH1/2 mutations and improved survival in patients with GBM.^29^

In addition to survival outcomes, IDH mutations define a distinct epigenetic subclass, G-CIMP, that is linked to a better prognosis. G-CIMP^+^ tumors, which frequently harbor IDH1 mutations, align with a proneural gene expression profile and are diagnosed at a younger age, whereas G-CIMP^−^ tumors, including most primary GBMs, exhibit a more aggressive phenotype.^30^ IDH status also influences surgical decisions, with supramaximal resection showing significant benefits in IDH mutant gliomas but a limited impact in IDH wild-type GBMs.^31^ At the molecular level, IDH mutations frequently cooccur with p53 mutations and 1p/19q codeletions^32^ but remain mutually exclusive with EGFR amplification and chromosome 10 loss,^33^ further reinforcing their role in glioma subtyping. The formal integration of the IDH status into the WHO glioma classification solidifies IDH mutations as essential diagnostic and prognostic molecular markers,^17,29,34^ emphasizing the need for molecularly driven therapeutic approaches to improve GBM patient outcomes.

#### MGMT promoter methylation

The *MGMT* gene is crucial for DNA repair and cellular defense, counteracting alkylating chemotherapy-induced damage by removing alkyl groups from the O^6^ position of guanine. Methylation status critically influences the GBM treatment response by regulating gene expression. Hypermethylation leads to transcriptional silencing, impairing the ability of tumors to repair alkylating agent-induced DNA damage and thereby increasing their sensitivity to temozolomide (TMZ). This epigenetic alteration is more prevalent in secondary GBMs than in primary GBMs or their precursor low-grade gliomas (LGGs) and serves as a robust predictive biomarker for chemotherapy efficacy, particularly in GBM.^35^ Clinical trials, including NOA-08, the Nordic trial, and RTOG 0525, have demonstrated that patients with MGMT-methylated tumors benefit significantly from TMZ treatment, resulting in prolonged PFS and OS.^36–38^ This predictive value is especially evident in elderly patients, where the MGMT status guides therapeutic decisions between chemotherapy and radiotherapy.^39^ Conversely, unmethylated MGMT tumors maintain their DNA repair capacity, diminishing the effectiveness of alkylating agents and correlating with poor outcomes.^40^ With approximately 50% of GBMs exhibiting MGMT promoter methylation, routine testing is increasingly recognized as essential for tailoring personalized treatment strategies. Notably, the MGMT methylation status outperforms conventional prognostic indicators such as tumor grade, performance status, and patient age in predicting therapeutic response, underscoring its clinical relevance. Future research should focus on strategies to overcome resistance in MGMT-unmethylated tumors, exploring novel therapeutic approaches to increase treatment efficacy. The continued evolution of molecular classification in GBM highlights the MGMT status as a crucial determinant of personalized treatment approaches, paving the way for improved patient outcomes.

### Imaging biomarkers

Although no clinically approved imaging biomarkers currently exist for GBM, advanced functional imaging techniques hold significant potential in tumor characterization and treatment planning. Methods such as diffusion-weighted magnetic resonance imaging (DW-MRI), dynamic susceptibility contrast-enhanced perfusion imaging, MR spectroscopy, and positron emission tomography (PET) offer valuable insights into tumor biology, genetic alterations, and therapeutic response.^41^ However, variations in sensitivity and specificity across studies highlight the need for standardized acquisition protocols and validation in clinical settings. Among promising imaging biomarkers, proton MR spectroscopy can detect 2-HG levels, which are correlated with IDH1/2 mutations, making it a noninvasive diagnostic and prognostic marker.^42^ Additionally, MRI-derived parameters such as apparent diffusion coefficient values,^43^ the T2-to-contrast-enhancing volume ratio, and relative cerebral blood volume have demonstrated predictive value for genetic alterations such as EGFR amplification and clinical outcomes such as PFS.^44^ Notably, increased tumor blood volume is strongly associated with an unmethylated MGMT status, further reinforcing the role of imaging biomarkers in guiding treatment response and prognosis.^45^

PET imaging plays a crucial role in GBM assessment, but the commonly used ^18^F-fluorodeoxyglucose (^18^F-FDG) PET has limited sensitivity because of high baseline glucose uptake in the brain, reducing its accuracy in detecting early recurrences and low-grade tumors. Consequently, alternative PET tracers, such as radiolabeled amino acids (11C-methionine [11C-MET], ^18^F-fluoroethyltyrosine [^18^F-FET], and ^18^F-fluoro-L-DOPA [^18^F-FDOPA]), along with hypoxia agents such as ^18^F-fluoromisonidazole (^18^F-FMISO), have gained prominence for their ability to visualize gliomas independent of BBB integrity. Studies indicate that higher 11C-MET uptake correlates with poorer survival, whereas ^18^F-FET and ^18^F-FDOPA effectively differentiate glioma grades and predict tumor proliferation,^46,47^ Hypoxia imaging using ^18^F-FMISO has emerged as a potential predictive biomarker that is correlated with tumor progression and decreased survival in GBM patients. Its ability to identify radiation-resistant tumor regions suggests applications in radiotherapy planning and treatment adaptation, providing critical insights for optimizing therapeutic strategies.^48^ These findings emphasize the growing relevance of molecular imaging in refining GBM prognosis and guiding personalized treatment approaches.

Despite promising results, further prospective validation is necessary before the integration of imaging biomarkers into routine clinical practice. The potential of these techniques to predict treatment response, detect early recurrence, and guide therapeutic strategies highlights their growing importance in GBM management. Future research should focus on optimizing imaging protocols, validating biomarkers across large patient cohorts, and integrating imaging data with molecular classification systems to enhance precision oncology approaches in GBM treatment.

### Circulating biomarkers

#### Biochemical biomarkers

Circulating biochemical biomarkers have emerged as potential noninvasive tools for GBM diagnosis and prognosis, reflecting the molecular and immunological landscape of the disease. These biomarkers include proteins, cytokines, and traditional cancer markers, many of which have altered expression levels in GBM patient body fluids. Notable proteins, such as glial fibrillary acidic protein, brain-derived neurotrophic factor, protein S100B, and neural cell adhesion molecules, have been identified as neuronal markers linked to GBM pathology. Additionally, metabolic and inflammatory biomarkers such as 2-HG, chitinase-3-like protein 1, interleukin-2 (IL-2), transforming growth factor-β (TGF-β), tumor necrosis factor-α (TNF-α), and matrix metalloproteinases (MMPs) have been implicated in GBM progression and immune modulation.^49^ Despite extensive research, many circulating biochemical biomarkers lack tumor specificity, limiting their diagnostic utility. However, advancements in proteomic profiling have led to the identification of more promising biomarker candidates. Recent studies have highlighted a panel of biomarkers with high diagnostic accuracy, with six markers demonstrating over 80% efficiency in distinguishing GBM from nontumor conditions. Among the most promising biomarkers, the overexpression of complement component C9 (C9), C-reactive protein, and leucine-rich α-2-glycoprotein (LRG1) is strongly correlated with GBM tumor burden and progression. Conversely, low expression of gelsolin, apolipoprotein A-IV and the Ig α-1 chain C region has also shown diagnostic significance. Importantly, the concentrations of C9, CRP, and LRG1 are significantly associated with tumor size, reinforcing their potential role in GBM prognosis and clinical stratification.^50^

The identification of circulating biochemical biomarkers represents a promising avenue for noninvasive GBM detection and monitoring. However, further validation in large-scale clinical studies is essential to establish their diagnostic reliability and prognostic value. Future research should focus on standardizing biomarker panels, integrating multiomics approaches, and improving specificity to increase the clinical utility of biochemical biomarkers in GBM management.

#### Circulating tumor cells

Circulating tumor cells (CTCs) play a pivotal role in GBM progression, offering valuable insights into tumor behavior, treatment response, and prognosis. The presence of these genes in the bloodstream correlates with tumor progression, recurrence, and the GBM subtype, establishing them as promising biomarkers for disease monitoring. As a noninvasive alternative to conventional biopsies, CTC-based liquid biopsy allows real-time tracking of tumor dynamics, enabling repeated assessments over time without the need for invasive procedures.^51^ The prevalence of CTCs in GBM exceeds 75%, with their levels directly reflecting the tumor burden and therapeutic response.^52^ A decrease in CTC counts post-therapy indicates treatment efficacy, whereas persistent or rising levels may suggest resistance to therapy. Additionally, CTC genetic profiling can be used to determine drug sensitivity, paving the way for personalized treatment strategies in GBM.^53^

CTCs represent a critical diagnostic and prognostic tool with potential applications in therapy selection and disease monitoring. Their quantification and molecular analysis provide insights into tumor evolution, facilitating precision oncology approaches.^53^ The integration of CTC assessment into routine clinical practice could enhance treatment personalization, improve early detection of therapeutic resistance, and optimize GBM management strategies. However, further standardization and validation in large-scale clinical studies are essential to establish their full clinical utility.

#### Circulating RNA

Circulating RNA biomarkers, including circRNAs, miRNAs, and lncRNAs, serve as powerful, noninvasive diagnostic tools in GBM, enabling early detection, precise prognosis assessment, and real-time monitoring of treatment response. Their presence in the bloodstream and cerebrospinal fluid (CSF) offers a unique opportunity to track tumor dynamics, paving the way for personalized therapeutic strategies and improved clinical outcomes in GBM management.

##### circRNAs

Dysregulated circRNA expression is a defining feature of GBM progression, influencing cell proliferation, metastasis, angiogenesis, and oncogenesis. High-throughput RNA sequencing and microarray analysis have identified numerous differentially expressed circRNAs in tumor tissues, highlighting their potential as diagnostic and prognostic biomarkers.^54^ Table S2 lists the circRNAs that serve as biomarkers for GBM and are involved in pathogenesis (Table S3). Studies have revealed that the expression of most circRNAs is greater in normal brain tissues than in GBM tissues, with only a few displaying elevated levels in tumor samples.^55^ Notably, circ-SMARCA5 is significantly downregulated in GBM, whereas circ-CFH and circ_0012129 are upregulated,^56^ indicating their distinct roles in tumor progression. Additionally, circRNA_0037655 and circ-MAPK4 promote tumor survival and invasion,^57^ whereas circ-E-cadherin and circ-XRCC5 are linked to GBM aggressiveness and poor prognosis.^58^ In contrast, circ-DCL1 suppresses tumor proliferation through METTL3-mediated m^6^A modification,^59^ highlighting the dual role of circRNAs as oncogenes and tumor suppressors.

In addition to promoting tumor proliferation, circRNAs interact with the TME to increase GBM progression. circ-NEIL3 stabilizes insulin-like growth factor (IGF)-2 mRNA binding protein 3, facilitating exosomal transfer to TAMs and thereby reinforcing their immunosuppressive functions.^60^ Moreover, circ-LGMN, which is significantly upregulated in HGGs, drives GBM malignancy by regulating legumain.^61^ The identification of circRNAs as potential biomarkers presents promising opportunities for noninvasive GBM diagnosis and personalized treatment strategies. Their expression profiles provide critical insights into tumor behavior, prognosis, and therapeutic response. However, further large-scale validation and functional studies are necessary to standardize circRNA-based biomarker panels, paving the way for their integration into clinical GBM management.

##### lncRNAs

lncRNAs have emerged as key prognostic biomarkers in GBM, offering insights into tumor progression, survival prediction, and therapy resistance. Table S4 presents the lncRNAs that serve as biomarkers for GBM and its pathogenesis (Table S5). Studies have revealed that several lncRNAs are strongly correlated with tumor grade, survival rates and treatment response, highlighting their clinical relevance. Among the most significant lncRNAs, the lncRNA MAGI2-AS3 is upregulated in GBM, and its expression is positively correlated with tumor grade and the Karnofsky performance score (KPS). Lower levels of the lncRNA MAGI2-AS3 are associated with poorer survival outcomes, making it an independent predictor of OS.^62^ Similarly, the lncRNA ELF3-AS1 is significantly elevated in tumor tissues, reinforcing its potential as a GBM-specific biomarker.^63^ Additionally, the lncRNA PXN antisense RNA-1 is overexpressed in GBMs and serves as an indicator of poor prognosis.^64^ The diagnostic value of N^6^-methylandenosine (m^6^A)-related lncRNAs has also been demonstrated in prognostic models incorporating m^6^A-LPS, age, and WHO grade, effectively predicting OS in LGG patients.^65^ Furthermore, elevated levels of the lncRNA HOTAIR in GBM patient serum further support the diagnostic utility of lncRNAs.^66^

lncRNAs also contribute to therapy resistance and immune regulation in GBM. Metastasis-associated lung adenocarcinoma transcript 1 (MALAT1) expression is linked to TMZ resistance, positioning it as a prognostic marker for chemoresistant GBMs.^67^ Immune-related lncRNAs, such as DiGeorge syndrome critical region gene 5, are associated with immune and stromal cell infiltration, highlighting their role in regulating the tumor immune response.^68^ Additionally, upregulation of the lncRNA CRNDE in GBMs is linked to tumor size, recurrence risk, and chemosensitivity to TMZ,^69^ reinforcing its role in predicting therapeutic response. Some lncRNAs, such as the lncRNA GAS5, are expressed at higher levels in LGGs than in GBM,^70^ suggesting their role in monitoring tumor progression. Conversely, the zinc finger E-box-binding homeobox 1 (ZEB1)-lncRNA AS1 and the lncRNA ANRIL are highly expressed in GBM and are correlated with tumor size and malignancy grade.^71^ However, further large-scale validation and functional studies are necessary to establish their clinical applicability. Integrating lncRNA-based biomarker panels into GBM diagnosis and personalized treatment strategies could enhance patient stratification, therapeutic decision-making, and overall clinical outcomes.

##### miRNAs

miRNAs have demonstrated significant potential as biomarkers for diagnosis, tumor grading, and monitoring treatment response in GBM.^72^ Among these, miR-21 is consistently upregulated in GBMs, with elevated levels detected in the CSF and serum of HGG patients, making it a reliable biomarker for early detection and disease progression.^73^ Additionally, the levels of miR-21, miR-222, and miR-124-3p are significantly elevated in HGGs compared with those in LGGs and healthy patients, with levels decreasing post-surgery,^74^ reinforcing their prognostic and diagnostic importance. Several miRNAs, such as miR-128 and miR-342-3p, are downregulated in GBM, increasing after surgery and chemoradiation, suggesting their potential as indicators of therapeutic efficacy. Similarly, miR-20a-5p, miR-106a-5p, and miR-181b-5p are associated with tumor progression, whereas miR-19a-3p, miR-106a-5p, and miR-181b-5p are linked to poor prognosis.^75^ Furthermore, miR-1238 is elevated in recurrent GBM, highlighting its role in disease monitoring and predicting recurrence risk.^76^

miR-301a expression is correlated with tumor progression and a reduced KPS, with exosomal levels dynamically changing following tumor resection and recurrence, making it a valuable biomarker for disease monitoring.^77^ Additionally, exosomal miR-210, miR-5194, and miR-449 target key genes in the EGFR and cellular mesenchymal epithelial transition (c-MET) signaling pathways and are correlated with histopathological grade and GBM aggressiveness.^78^ Table S6 presents the miRNAs that serve as biomarkers associated with GBM pathogenesis. Some miRNAs, such as miR-524-3p and miR-524-5p, are downregulated in GBM and associated with EGFR overexpression and EGFRvIII mutation, while their overexpression inhibits tumor proliferation and migration, improving OS through the TGF-β, Notch, and Hippo pathways.^79^ Similarly, low miR-133 levels correlate with poor prognosis, as its overexpression inhibits EGFR mRNA translation, suppresses GBM growth and induces apoptosis.^80^ Conversely, miR-148a functions as an oncogene, negatively impacting survival through its regulation of BIM, MIG6, and EGFR,^81^ making it a potential therapeutic target.

miRNA expression profiling offers a noninvasive and dynamic approach for GBM diagnosis, prognosis, and treatment monitoring. miR-34a deletion and EGFR amplification are linked to poor survival, whereas high miR-340 and miR-615 expression are correlated with longer overall and recurrence-free survival,^82,83^ reinforcing their potential as independent prognostic factors. The identification of circulating miRNAs in serum and plasma provides a powerful tool for personalized GBM management, allowing early detection, prediction of therapeutic response, and disease monitoring. However, further validation in large-scale clinical studies is essential to fully integrate miRNAs into routine GBM diagnostics and treatment planning.

#### Circulating DNA

The analysis of circulating tumor DNA (ctDNA) provides a noninvasive approach for disease monitoring and treatment response assessment in GBM. Studies have demonstrated that circulating cell-free DNA levels fluctuate throughout treatment, with elevations before surgery and at disease progression, reinforcing its potential as a dynamic biomarker.^84^ Importantly, next-generation sequencing and methylation assays have identified key genetic alterations in ctDNA, including mutations in genes such as *p53*, *EGFR*, *MET*, *PIK3CA*, and *NOTCH1*, highlighting the feasibility of liquid biopsies in molecular profiling and personalized therapy selection.^85^ The detection rates of ctDNA in GBM remain variable, with 51% of advanced primary GBM patients exhibiting detectable ctDNA, some of whom have genomically targetable mutations.^86^ Notably, somatic alterations in genes such as *p53*, *JAK2*, *NF1*, *EGFR*, *BRAF*, *IDH1*, *NRAS*, *GNAS* and ataxia telangiectasia mutated (*ATM*) further illustrate the genetic heterogeneity of GBM,^87^ underscoring the importance of ctDNA in tumor characterization. Additionally, CSF-based ctDNA analysis has shown higher sensitivity than plasma ctDNA analysis,^87^ suggesting that CSF-based ctDNA analysis is a more reliable method for tumor-specific genetic assessment.

ctDNA has demonstrated potential in detecting drug resistance mutations in patients receiving kinase inhibitor therapy, aiding in treatment adaptation and precision oncology approaches. Furthermore, integrated platforms analyzing key genes such as *IDH1*, *IDH2*, *p53*, *ATRX*, *TERT*, and H3 histone family 3 A (*H3F3A*) enable more efficient subclassification of diffuse gliomas.^88^ However, ctDNA detection remains challenging in localized tumors such as GBMs, emphasizing the need for further optimization of ctDNA extraction and analysis methods. As liquid biopsy technology advances, refining ctDNA-based assays will be crucial in enhancing early detection, disease monitoring, and therapeutic decision making in GBM.

#### Extracellular vesicles

EVs have emerged as promising noninvasive biomarkers for GBM and play critical roles in tumor progression, intercellular communication, and treatment response monitoring. GBM and stromal cells release tumor-associated EVs into bodily fluids such as plasma, serum, CSF, and urine, providing an accessible liquid biopsy tool for disease monitoring and molecular profiling.^89^ Elevated EV concentrations in the peripheral blood of GBM patients, independent of specific molecular alterations (EGFR amplification, PTEN deletion, MGMT expression, and IDH mutations),^90^ suggest their broad applicability in GBM detection, prognosis, and relapse prediction. Additionally, fluctuations in EV concentrations are correlated with surgical resection and recurrence,^91^ reinforcing their potential as dynamic biomarkers. Table S7 lists the EVs that serve as biomarkers for GBM diagnosis.

In addition to their presence in the circulation, EVs carry molecular cargo, including DNA, RNA, and proteins, reflecting the genetic and epigenetic landscape of tumors. Plasma EV-based markers such as EGFR, EGFRvIII, and IDH1-R132H mutations have demonstrated high specificity for GBM classification and subtyping. The tumor progression index, which incorporates EV counts and molecular cargo^,^^92,93^ effectively differentiates treatment responders from nonresponders, offering a refined tool for therapy monitoring. The detection of IDH1 mutations in EV-derived DNA from plasma and CSF provides a minimally invasive alternative to conventional tissue biopsies, enabling a comprehensive molecular assessment of GBM. The presence of EGFRvIII in CSF-derived EVs, even when it is absent in tissue biopsies,^94^ underscores the superiority of EVs in capturing tumor heterogeneity,^95^ offering insights into oncogenic signaling and tumor progression.

EV-based biomarkers show potential for assessing treatment response and predicting patient outcomes. Studies indicate that PTEN and MGMT mRNA levels in GBM-derived EVs (GDEVs) correlate with tumor grade and therapy response,^96^ whereas miR-21 in CSF-derived EVs is linked to poor prognosis.^73^ Moreover, EV-associated epigenetic modifications, including DNA methylation, reflect the molecular profile of tumors,^97^ supporting their role in real-time GBM monitoring. With increasing evidence supporting the use of EV-based biomarkers, their integration into clinical GBM management could revolutionize diagnosis, treatment response assessment, and personalized therapy strategies. However, further validation through large-scale studies is essential to standardize EV-based assays for routine clinical application in GBM.

## Regulatory mechanisms in GBM pathogenesis

### Epigenetic characteristics of GBM

**Fig. 3:**
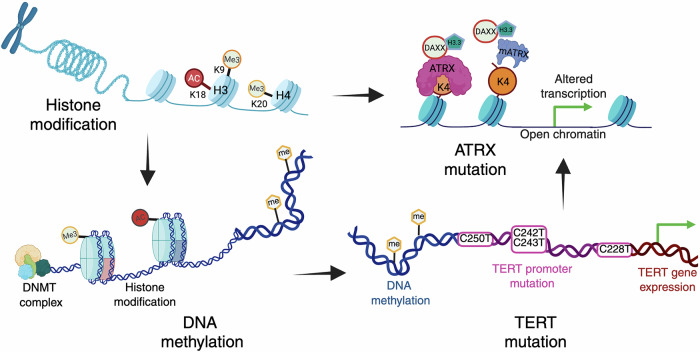
Epigenetic characteristics of glioblastoma and their role in pathogenesis. The figure depicts the key epigenetic mechanisms contributing to glioblastoma (GBM) development, including histone modifications, DNA methylation, ATRX mutations, and TERT promoter mutations. Histone modifications such as methylation (Me3) and acetylation (Ac) at specific lysine residues regulate chromatin accessibility and gene expression. DNA methylation, which is catalyzed by DNA methyltransferases (DNMTs), further influences gene silencing or activation. ATRX mutations impair chromatin remodeling by disrupting the ATRX-DAXX complex, which is responsible for H3.3 deposition, leading to altered transcription and increased chromatin accessibility. TERT promoter mutations result in aberrant telomerase expression, contributing to tumor cell immortality

GBM pathogenesis is driven by a combination of extensive genetic and epigenetic alterations that regulate gene expression and tumor progression. Among these, epigenetic changes, such as histone modifications, DNA methylation, and chromatin remodeling, play a central role in tumor biology. Aberrant histone methylation and acetylation, ATRX mutations impacting chromatin stability, and widespread promoter hypermethylation, including MGMT, disrupt the balance between tumor suppressor genes and oncogenic pathways. Furthermore, TERT promoter mutations activate telomerase, enabling replicative immortality, whereas copy number alterations exacerbate the dysregulation of key cellular pathways. The intricate crosstalk between these epigenetic mechanisms drives genomic instability, tumor proliferation, and therapy resistance, highlighting their importance in GBM pathogenesis and their potential as promising therapeutic targets (Fig. 3).

#### Histone modification

Histone modifications are crucial regulators of gene expression, influencing GBM progression, tumor proliferation, and therapy resistance. Histones undergo various modifications, including acetylation and methylation. Acetylation typically promotes gene activation, whereas methylation can either enhance or repress transcription, depending on the specific histone site.^98^ Disruptions in these processes contribute to GBM aggressiveness and treatment resistance.^99^ Distinct histone modification patterns are correlated with prognosis; for example, lower H3K18 acetylation (H3K18Ac) is linked to improved survival in primary GBM, whereas higher H4K20 trimethylation (H4K20me3) is associated with better outcomes in secondary GBM. Additionally, H3K9 trimethylation (H3K9me3), a transcriptional repressor, is linked to IDH mutant gliomas, distinguishing them from wild-type GBM.^100^

Mutations in H3F3A, including H3.3 and H3.1, frequently occur in pediatric GBM and drive distinct epigenetic alterations. The K27M mutation disrupts histone methylation and acetylation, whereas the G34R/G34V mutations alter transcription regulation. These mutations alone do not initiate tumor formation but act alongside additional genetic changes.^101^ The H3K27M mutation inhibits the polycomb repressive complex 2 chromatin-modifying complex, influencing the transcriptional programs associated with pediatric GBM.^102^ Enhancer of zeste homolog 2 (EZH2) overexpression further drives oncogenic pathways, including c-Myc activation, which is correlated with poor prognosis. Targeting EZH2 suppresses tumor growth, enhances radiation sensitivity, and disrupts GSC maintenance, making it a promising therapeutic approach. Similarly, dysregulation of protein arginine methyltransferase 1 (PRMT1) and PRMT2 alters histone methylation, further driving GBM progression and therapy resistance.^103^

Lysine demethylases (KDMs) and HDACs regulate tumor proliferation, cell death, and therapy resistance in GBM. KDM5A overexpression contributes to TMZ resistance, and its inhibition enhances the treatment response.^104^ HDAC9, a regulator of Hippo signaling via TAZ activation, promotes GBM progression, highlighting HDAC9 inhibition as a therapeutic strategy.^105^ Ras-related protein on chromosome 22 (RRP22) functions as a tumor suppressor, with low expression linked to increased tumor grade and reduced survival. Its downregulation is associated with 5’-CpG island hypermethylation and altered histone acetylation (H3/H4 acetylation loss). In primary GBM, elevated H3K9me3 levels and reduced pan-Ac-H3-bound RRP22 expression further implicate epigenetic dysregulation in tumor progression.^106^ Targeting EZH2, KDMs, and HDACs offers promising avenues for overcoming treatment resistance and improving therapeutic outcomes. Understanding the interplay between histone modifications and transcriptional regulation is essential for advancing precision medicine strategies in GBM treatment.

#### ATRX mutation

ATRX mutations play a critical role in GBM pathogenesis by disrupting chromatin remodeling, telomere maintenance, and DNA repair. ATRX loss is associated with alternative lengthening of telomeres, a hallmark of genomic instability, and is predominantly observed in LGGs with IDH mutations and without 1p/19q codeletion.^107^ Although rare in adult primary GBM, ATRX mutations are more prevalent in younger patients and secondary GBMs, where they correlate with improved prognosis. Their presence offers potential as prognostic biomarkers and therapeutic targets in GBM. ATRX deficiency has been shown to accelerate GBM growth and reduce survival, linking ATRX loss to DNA repair deficiencies, particularly impaired nonhomologous end joining.^108^ These findings suggest that ATRX-deficient GBMs are vulnerable to therapies targeting DNA damage repair pathways.

In pediatric GBM, ATRX mutations contribute to genetic instability, influencing mutation rates and molecular subtypes. Studies have revealed that ATRX mutations in grade II–III astrocytomas, oligoastrocytomas, and secondary GBMs often cooccur with IDH1 mutations and ALT activation.^109^ Additionally, the H3.3–ATRX–DAXX chromatin remodeling complex is frequently altered in pediatric GBMs, underscoring the role of ATRX in tumor development.^101^ These findings emphasize that ATRX mutations are key molecular markers for glioma classification and potential therapeutic intervention. Further exploration of ATRX-related pathways may provide novel treatment strategies for ATRX-mutated GBMs, particularly through targeted approaches that disrupt ALT and DNA repair mechanisms.

#### DNA methylation

DNA methylation, which is mediated by DNA methyltransferases, is a critical epigenetic modification that influences gene expression, tumor progression, and therapeutic response in GBM. Advances in genome-wide methylation profiling have significantly improved tumor classification, prognosis, and treatment stratification.^110^ DNA methylation patterns provide insights into molecular subtypes, with studies demonstrating their accuracy in predicting key glioma features such as IDH mutations and 1p/19q codeletions.^111^ These findings highlight methylation profiling as a powerful diagnostic and prognostic tool that complements histopathological classification. The identification of methylation signatures, such as those distinguishing IDH mutant and IDH wild-type GBMs, provides a framework for personalized treatment strategies. The integration of methylation biomarkers, including three-gene signatures (*EMP3*, *GSX2*, and *EMILIN3*), has demonstrated prognostic potential in GBM patients,^112^ allowing for more precise risk assessment and therapeutic decision-making.

In addition to classification, the DNA methylation status is instrumental in predicting therapy response. Recent studies have linked low DNMT1 expression with TMZ resistance, suggesting that alterations in DNA methylation may serve as indicators of treatment efficacy.^113^ Emerging research has also identified methylation patterns in DNA damage response (DDR) genes, including MGMT, MLH3, RAD21, and SMC4, as potential biomarkers for therapy response prediction.^114^ Findings from the EORTC 22033 phase III trial further underscore the clinical relevance of molecular stratification in GBM treatment. While no overall difference in progression-free survival was observed between dose-dense TMZ and radiotherapy, IDH mutant, 1p/19q codeleted tumors responded more favorably to chemotherapy.^115^ This finding reinforces the role of DNA methylation profiling in optimizing treatment regimens. The continued exploration of DNA methylation in GBM pathogenesis highlights its potential for refining diagnostic models, improving prognostic assessments, and guiding personalized therapies. Future research should focus on integrating methylation-based classifiers into clinical practice, further validating their predictive utility, and exploring novel epigenetic targets for therapeutic intervention.

#### Copy number alterations

Copy number alterations (CNAs) significantly impact genomic integrity, leading to the emergence of driver amplifications and deletions that disrupt crucial genes. Widespread chromosomal abnormalities, including losses on chromosomes 9 and 10 and polysomy of chromosomes 7, 19, and 20, are recurrently observed in GBM. Key focal alterations include *CDKN2A/B* deletions and high-level *EGFR* amplifications, which contribute to tumor progression and therapy resistance.^116^ Recent studies highlight the importance of CNA profiling in stratifying GBM patients and guiding clinical decision-making. Molecular characterization of CNAs improves the selection of treatment strategies, emphasizing the need for integrating CNA data into clinical trial designs to ensure more representative patient cohorts.^117^ Additionally, emerging findings suggest that both frequent and patient-specific CNAs influence survival outcomes, underscoring their potential for refining prognostic models.^118^ Computational analyses, such as those utilizing Oncoscape, have further demonstrated the prognostic significance of CNAs in GBM and diffuse gliomas. Multidimensional molecular grouping has enabled visualization of glioma classifications on the basis of CNAs, correlating specific chromosomal alterations with distinct survival outcomes. The identification of CNA-driven molecular subtypes reinforces their predictive value, highlighting critical genomic variations that could inform targeted therapeutic strategies.^119^ The incorporation of CNA profiling into routine clinical practice holds promise for improving patient stratification, treatment selection, and outcome prediction. Future research should focus on leveraging CNA data to refine GBM classification systems and develop personalized therapeutic approaches.

#### TERT promoter mutation

Mutations in the promoter region of the *TERT* gene have emerged as key molecular alterations in gliomas, influencing tumor progression, prognosis, and treatment response. These mutations, which primarily occur at C228T and C250T, create novel Ets/TCF binding sites, leading to aberrant TERT expression and sustained telomerase activity.^120^ The high prevalence of these genes in GBMs and other diffuse GBMs highlights their role in tumor maintenance and resistance to apoptosis. Clinical studies emphasize the prognostic importance of TERT promoter (TERTp) mutations, particularly in the context of other molecular alterations. In diffuse gliomas, TERTp mutations are associated with worse OS, with distinct prognostic implications depending on tumor grade and cooccurring mutations. For example, in Grade II and III gliomas, survival outcomes vary significantly on the basis of the interplay between TERTp mutations, MGMT methylation, IDH mutation, and 1p/19q codeletion.^121^ Notably, patients with IDH mutant gliomas and concurrent TERTp mutations have poorer prognoses than those with IDH mutations alone, underscoring the complex molecular interactions governing glioma progression.

The frequency and prognostic impact of TERTp mutations differ across glioma subtypes. Oligodendrogliomas, characterized by IDH mutation and 1p/19q codeletion, present the highest prevalence of TERTp mutations. In contrast, anaplastic astrocytomas and IDH wild-type GBMs also harbor these mutations but have varying prognostic outcomes. IDH wild-type GBMs with TERTp mutations exhibit particularly poor survival, reinforcing their value as prognostic biomarkers in this aggressive glioma subtype.^122^ These findings highlight the necessity of integrating the TERTp mutation status into glioma classification and clinical decision-making. Beyond prognostication, ongoing research into the mechanistic role of TERTp mutations may provide insights into novel therapeutic targets, potentially leading to the development of telomerase-directed therapies aimed at improving outcomes for GBM patients.

#### Loss of heterozygosity

Loss of heterozygosity (LOH) is a common genomic alteration in GBM that drives tumor progression by disrupting tumor suppressor genes. LOH occurs across several chromosomal regions, including 9p, 10q, 17p, 19q, and 22, with LOH at chromosome 10q being one of the most frequent and significant events in primary GBM, affecting approximately 70% of cases.^123^ Notably, LOH at 10q is more prevalent in older patients, suggesting a potential age-related influence on GBM tumorigenesis. The prognostic significance of LOH 10q is well established, particularly in differentiating primary from secondary GBM.^124^ LOH at 10q25-qter is highly specific for secondary GBM, whereas broader loss of 10q is associated with both primary and secondary subtypes. In contrast, LOH at 1p and 19q, although key molecular markers for oligodendrogliomas, lacks prognostic or predictive relevance in GBM.^125^ The tumor suppressor genes affected by LOH 10q, particularly PTEN, p53, and NF1, play crucial roles in regulating cell survival and proliferation. Among these, PTEN loss is particularly consequential, as it leads to dysregulation of the PI3K/AKT pathway, promoting unchecked cell growth and therapy resistance.^126^ Given the role of LOH 10q in GBM pathogenesis, integrating LOH analysis into molecular profiling could enhance prognostic assessment and guide targeted therapeutic strategies aimed at restoring tumor suppressor function or counteracting downstream oncogenic pathways.

#### 1p/19q codeletion

The 1p/19q codeletion is a well-established prognostic biomarker in gliomas, particularly in oligodendrogliomas, where it is correlated with prolonged PFS and OS. This genetic alteration defines a distinct molecular glioma subtype, aiding in tumor classification and therapeutic decision-making. In LGGs, the iso-deletion of chromosome 1p alone is associated with a prognosis comparable to that of the full 1p/19q codeletion, whereas the iso-deletion of 19q alone also confers prolonged PFS.^127^ The frequency of 1p/19q codeletion varies among glioma subtypes, with the highest prevalence in oligodendrogliomas (WHO grade III) and a lower occurrence in astrocytomas. This alteration is strongly associated with IDH mutations and is almost mutually exclusive with ATRX mutations, reinforcing its role as a key molecular marker in glioma classification. Clinically, 1p/19q codeletion is linked to increased chemosensitivity, particularly in LGGs that respond favorably to TMZ-based therapy.^128^ Studies have demonstrated that patients with 1p/19q codeletion derive significant survival benefits from combined treatment with procarbazine, lomustine, and vincristine (PCV) chemotherapy alongside radiotherapy compared with radiotherapy alone.^129^ These findings emphasize its predictive role in optimizing treatment strategies. Given its strong association with favorable treatment response and prolonged survival, integrating the 1p/19q codeletion status into routine GBM management enhances personalized treatment planning and improves patient outcomes.

#### Fusion genes

Advances in sequencing technologies have led to the identification of oncogenic fusion genes in GBM, including those encoding FGFR, ALK, and EGFR, and neurotrophic tyrosine receptor kinase fusions. FGFR fusions are the most common, present in 8.33% of cases, followed by EGFR (4%) and ALK (1.9%), with the latter being more prevalent in pediatric GBM.^130^ NTRK1 fusions, although rare (1.2%), may contribute to GBM oncogenesis.^131^ Clinically, inhibitors such as lorlatinib and larotrectinib show promise in targeting fusion-positive GBMs.^132,133^ FGFR3-TACC3, a recurrent fusion protein, drives tumorigenesis by promoting kinase transphosphorylation and disrupting chromosomal stability. This fusion is mutually exclusive with IDH1/2 mutations and EGFR amplification, suggesting its role as an independent driver of GBM progression.^130^ Additionally, the PTPRZ1-MET fusion represents another oncogenic event, warranting further investigation as a potential therapeutic target.^134^ The identification of these fusions highlights the importance of personalized treatment strategies in GBM, emphasizing the need for continued research into targeted therapies that exploit these unique molecular alterations.

### Genetic alterations in GBM

**Fig. 4:**
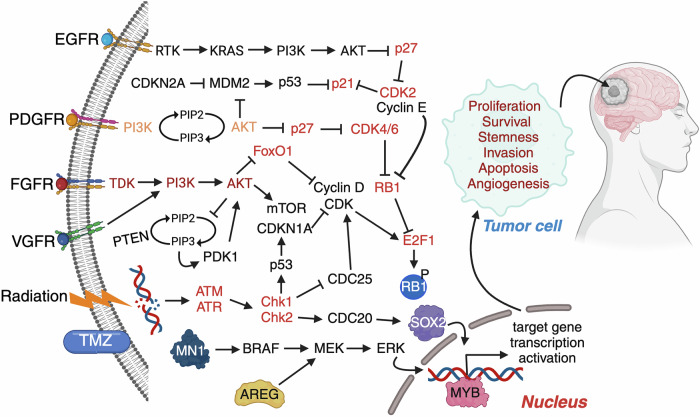
Genetic alterations driving glioblastoma pathogenesis. The schematic illustrates key oncogenic genetic alterations contributing to glioblastoma (GBM) development and progression. Receptor tyrosine kinases (RTKs), such as EGFR, PDGFR, FGFR, and VGFR, initiate downstream signaling cascades, including the PI3K/AKT/mTOR and RAS/RAF/MEK/ERK pathways. Loss of tumor suppressors (e.g., PTEN, CDKN2A, RB1, p53) and overactivation of oncogenes (e.g., EGFR, MDM2, CDK4/6, TERT, MYB, SOX2, AREG) promote cell cycle progression, proliferation, stemness, survival, angiogenesis, and resistance to apoptosis. DNA damage response elements (ATM/ATR-Chk1/Chk2) are activated by radiation and chemotherapy (TMZ) but are frequently bypassed in GBM. Downstream transcriptional regulators such as MYB and SOX2 further enhance tumor cell plasticity and malignancy. Collectively, these alterations reprogram the tumor cell phenotype, driving GBM progression and therapy resistance

Genetic alterations are fundamental to GBM pathogenesis, driving its aggressive growth and therapeutic resistance. Amplifications and mutations in RTKs, such as EGFR, PDGFR, and fibroblast growth factor receptors, lead to dysregulated signaling, promoting tumor proliferation and survival. Oncogenes such as MYB (myeloblastosis transcription factor), meningioma 1 (MN1), progranulin (PGRN) and amphiregulin (AREG) contribute to abnormal transcriptional activity and tumor progression. Concurrently, the loss or mutation of critical tumor suppressor genes, including p53 and PTEN, disrupts cell cycle regulation and DNA repair, fostering genomic instability. Deletions in CDKN2A (cyclin-dependent kinase inhibitor 2A) impair the cell cycle checkpoint, whereas aberrant activation of stem cell markers such as SRY-Box transcription factor 2 (SOX2) supports tumor cell self-renewal and invasion. These genetic changes collectively form the backbone of the highly malignant nature of GBM, underscoring the complexity of its molecular landscape and the challenges in developing effective treatments (Fig. 4).

#### EGFR

The amplification and mutation of EGFR, particularly the EGFRvIII variant, are defining characteristics of GBM, especially in the classical subtype and primary GBM cases. EGFR, a key tyrosine kinase receptor, regulates critical growth factors involved in tumor proliferation and survival. The EGFRvIII mutation results from a deletion of exons 2 and 7, eliminating the extracellular ligand-binding domain and leading to constitutive receptor activation. This alteration, driven by histone modifications at the EGFR enhancer region on chromosome 7p12, contributes to uncontrolled tumor growth and resistance to apoptosis.^135^ EGFR-mediated activation of the RTK/RAS/PI3K signaling axis disrupts the G1/S checkpoint, facilitating unchecked cell cycle progression.^136^ Patients with EGFRvIII mutations exhibit worse survival outcomes than those with wild-type EGFR, with coexpression of both forms further exacerbating tumor aggressiveness. This is attributed to cross-phosphorylation between EGFRvIII and wild-type EGFR, amplifying oncogenic signaling cascades.^137^ Notably, EGFRvIII expression is correlated with increased tumor heterogeneity, complicating treatment responses and limiting the efficacy of targeted therapies, including tyrosine kinase inhibitors and immunotherapy.^138^

In addition to the classical RTK/RAS/PI3K pathway, EGFRvIII activation has been linked to alternative tumorigenic mechanisms involving forkhead box G1 protein (FOXG1) and SOX9,^139^ which contribute to GBM stemness and invasive potential. These findings underscore the complexity of EGFR-driven oncogenesis and the necessity for precision-based therapeutic strategies. Given the resistance of EGFR-altered tumors to conventional EGFR inhibitors, ongoing research into combination therapies and novel targeted approaches remains critical for improving treatment efficacy in EGFR-mutant GBM. Understanding the molecular interplay between EGFR mutations and tumor behavior is essential for advancing therapeutic interventions and optimizing patient outcomes.

#### PDGFR

A distinct subset of GBMs, classified as the PDGFR subclass, accounts for approximately 25–30% of cases and is characterized by aberrant PDGFR signaling. The dysregulation of PDGFR in these tumors arises through various genetic mechanisms, including *PDGFRA* gene amplification, chromosomal rearrangements, and the overexpression of PDGF ligands.^140^ These alterations contribute to enhanced tumor cell proliferation, survival, and invasion. Age-related differences in PDGF signaling have been observed in GBM. Tumors in patients over 65 years of age exhibit significantly higher PDGFA expression levels than those in younger individuals do, with an increased PDGFA/PDGFRA expression ratio.^141^ In contrast, pediatric GBM patients show a greater prevalence of *PDGFRA* amplification than adult GBM patients do.^142^ This amplification is notably associated with tumors affecting the corpus callosum and is frequently linked to the aggressive H3K27M mutation found in diffuse midline gliomas.^143^ Despite its frequent occurrence, the prognostic significance of *PDGFRA* amplification remains uncertain. While some studies have associated *PDGFRA* amplification with poor survival (PS) outcomes, particularly in diffuse midline gliomas, its predictive value as an independent biomarker in GBM remains debated. Further research is needed to clarify its role in disease progression and response to targeted therapies. Given the therapeutic challenges associated with PDGFR-driven GBM, ongoing investigations into PDGFR inhibitors and combination treatment strategies could provide new avenues for improving patient outcomes.

#### FGFR

Lesions with FGFR1-TKDD mutations are primarily diffuse gliomas located in the cerebral cortex. Duplications of the FGFR1 TKD have also been found in low-grade astrocytomas, including pilocytic astrocytomas and dysembryoplastic neuroepithelial tumors (DNETs), which are typically located outside the cerebellum.^144^ These mutations are notable features of low-grade neuroepithelial tumors (LGNTs), occurring in 7.4% to 24% of cases, but they are rare in HGGs. In a study screening 33 HGG cases for FGFR1 region duplication in the tyrosine kinase domain, only one tumor was found to be positive for FGFR1-TKDD. This tumor, which was diagnosed as an anaplastic oligoastrocytoma (WHO grade III) that had progressed from a grade II tumor, exhibited FGFR1-TKDD positivity. Notably, FGFR1-TKDD has not been identified in adult-type oligodendrogliomas with IDH mutations and 1p/19q codeletion.^145,146^ Additionally, there was a case report of a glioneuronal tumor with features of both pilocytic astrocytoma and pleomorphic xanthoastrocytoma, which also carried FGFR1-TKDD and showed focal increases in mitotic activity.^147^ These findings highlight the range of gliomas associated with FGFR1-TKDD mutations and the need for further study to understand their clinical significance and potential treatment approaches.

#### MYB

MYB transcription factors, including MYBL1, function as proto-oncogenes that regulate progenitor cell proliferation and differentiation. In GBMs, *MYB* gene alterations are more common in young children and primarily affect tumors in the cerebral hemispheres. cIMPACT-Now Update 4 highlights the importance of integrated diagnostics in assessing WHO grade II IDH wild-type/H3-wild-type diffuse gliomas, particularly those with *MYB* or *MYBL1* rearrangements.^148^ These mutations are associated with a favorable prognosis, with gliomas harboring *MYB* or *MYBL1* alterations demonstrating prolonged disease stability and high OS rates. Reports indicate a 10-year OS rate of 90% and a 10-year PFS rate of 95%. The WHO CNS5 classification introduced diffuse astrocytoma, *MYB*- or *MYBL1*-altered, as a distinct entity within pediatric-type diffuse LGGs, designating it as a CNS WHO grade I tumor. *MYB* alterations are also highly prevalent in angiocentric gliomas, reinforcing their role in glioma subtyping.^149^ Future research will likely focus on MYB- and MYBL1-driven oncogenic mechanisms, particularly in pediatric LGGs, to refine diagnostic classification and identify targeted therapeutic strategies. Understanding MYB-driven pathways may lead to more personalized treatment approaches, potentially minimizing the need for aggressive therapies while maintaining favorable survival outcomes.

#### MN1

The *MN1* gene, located on chromosome 22q, functions as a transcriptional coregulator and is frequently altered in astroblastomas, a rare glioma subtype predominantly affecting pediatric and young adult populations. The WHO CNS5 classification designates astroblastomas with *MN1* alterations as a distinct molecular entity, yet further research is needed to differentiate them from other neuroepithelial tumors with overlapping genetic features. *MN1* alterations have emerged as potential prognostic markers, with studies indicating improved PSF and OS in gliomas exhibiting *MN1* rearrangements.^150,151^ Compared with BRAF V600E-mutated pleomorphic xanthoastrocytomas, *MN1*-rearranged astroblastomas have a more favorable prognosis.^152^ However, the mechanistic role of MN1 in tumorigenesis remains unclear, necessitating further studies to elucidate its functional impact on glioma biology and its potential utility in guiding clinical decision-making. Expanding the molecular characterization of *MN1*-altered gliomas could increase diagnostic accuracy and inform targeted therapeutic approaches.

#### PGRN and AREG

PGRN and AREG have emerged as critical players in GBM pathogenesis, each contributing uniquely to tumorigenesis, progression, and therapeutic resistance. PGRN, a member of the adipokine family, has gained attention for its elevated expression in GBM tissues compared with that in normal brain tissue, where it is correlated with increased tumor cell proliferation, pathological grading, and disease severity.^153^ Notably, PGRN levels in patient serum mirror those in tumor tissues, with higher expression linked to poorer overall and disease-free survival, particularly in LGGs.^154^ Multivariate analysis has identified PGRN as an independent prognostic factor, emphasizing its potential as a diagnostic and prognostic biomarker.^155^ Similarly, AREG, an EGFR ligand, plays crucial roles in GBM progression, drug resistance, and oncogenesis. AREG knockdown enhances doxorubicin (DOX)-induced endoplasmic reticulum stress, triggering autophagy and apoptosis and leading to GBM cell death. Bioinformatics analysis revealed that AREG is highly expressed in GBM and is correlated with PS.^156^ Additionally, AREG expression and methylation levels vary with astrocytoma grade, with GBM exhibiting higher mRNA expression but lower protein levels and increased methylation. Survival analysis revealed that AREG expression and methylation significantly impact patient prognosis, independent of astrocytoma grade.^157^ Furthermore, AREG is upregulated in microglia via colony-stimulating factor 1 receptor (CSF-1R) signaling, promoting GBM cell invasion. Blocking AREG through RNA interference or antibodies significantly reduces invasion, and the CSF-1R-MAPK/ERK pathway regulates its expression. Inhibiting ERK prevents microglia-stimulated invasion, and microglia require cell‒cell contact to increase invasion.^158^ Both PGRN and AREG are being explored as therapeutic targets, with preclinical studies investigating monoclonal antibodies, small-molecule inhibitors, and combination therapies to overcome resistance and improve outcomes. The dual roles of these genes as prognostic biomarkers and drivers of tumorigenesis make them promising candidates for advancing GBM research and treatment strategies.

#### SOX2

SOX2 is a critical regulator in GBM that influences key developmental pathways and contributes to tumor progression. Its overexpression is associated with increased proliferation, invasion, and self-renewal, particularly in GSCs.^159^ SOX2 is widely overexpressed across GBM but absent in normal central nervous system tissues,^160^ reinforcing its potential as a diagnostic and prognostic biomarker. High SOX2 levels are correlated with tumor aggressiveness and poor prognosis, making it a target of interest for therapeutic intervention. Studies have revealed a strong correlation between SOX2 expression and GBM malignancy, with the highest levels detected in aggressive GBM and oligodendrogliomas. SOX2 is particularly overexpressed in GBM, distinguishing malignant tissues from normal brain and nonmalignant tissues. SOX2-expressing cells are resistant to TMZ, but targeting SOX2 with inhibitors such as rapamycin has been shown to sensitize GBM cells to treatment,^159^ suggesting a potential strategy to increase therapeutic efficacy. Molecular profiling of GBM samples revealed frequent SOX2 amplification and overexpression, supporting its role in gliomagenesis. High SOX2 expression alone is sufficient to drive GBM cell invasion and migration. Additionally, silencing SOX2 in tumor-initiating cells (TICs) reduces tumor proliferation and tumorigenicity, emphasizing its functional importance in GBM progression.^161,162^ These findings underscore the importance of SOX2 as a biomarker for glioma classification and prognosis while highlighting its potential as a therapeutic target to improve treatment outcomes in aggressive brain tumors.

#### p53

p53 plays a crucial tumor-suppressive role in regulating cell cycle arrest, apoptosis, and DNA repair. Its function is tightly controlled by murine double minute (MDM) 2 and MDM4, which regulate p53 stability and activity through negative feedback mechanisms. While p53 alterations are less emphasized than other GBM markers, they are still significant in tumor pathogenesis. p53 mutations frequently occur early in gliomagenesis and accumulate as tumors progress. These alterations are particularly prevalent in the proneural GBM subtype, in contrast with the lower frequency in the classical subtype.^19,20,163^ The ARF-MDM2-p53 pathway is a major regulatory axis in GBM. The deletion of the CDKN2A/ADP-ribosylation factor (ARF) locus, which is observed in approximately 60% of GBM cases, contributes to p53 inactivation by impairing ARF-mediated MDM2 degradation. This disruption promotes tumor proliferation, invasion, and resistance to apoptosis.^164^ Additionally, MDM2 and MDM4 overexpression further suppresses p53 activity, leading to impaired DNA repair and enhanced tumor progression.^165^ Notably, MDM4-mediated p53 suppression is more common in classical GBM. Collectively, genetic alterations within the p53/MDM2/p14ARF pathway, including p53 mutations, MDM2 amplification, and p14ARF deletions, constitute major drivers of GBM pathogenesis.^166^

Targeting the p53/MDM2/p14ARF pathway represents a promising therapeutic avenue. Strategies aimed at restoring p53 function, including MDM2/MDM4 inhibitors and gene-editing approaches, could reactivate its tumor-suppressive role. Understanding how p53 mutations vary across GBM subtypes may enable more tailored therapeutic interventions. Given the high frequency of p53-related alterations, therapies targeting this pathway could improve GBM treatment outcomes by reinstating p53-driven tumor suppression.

#### CDKN2A

CDKN2A is a critical tumor suppressor gene that is frequently deleted or inactivated in GBM and LGGs. Its loss is associated with tumor progression, poor prognosis, and resistance to therapy. CDKN2A inactivation, primarily through homozygous deletion or promoter methylation, disrupts cell cycle regulation by impairing p16INK4a and p14ARF functions, leading to unchecked proliferation and reduced apoptosis. Genome-wide association studies have identified CDKN2A as a susceptibility locus for GBM, further highlighting its role in tumorigenesis.^167,168^ CDKN2A deletion is strongly linked to worse OS in astrocytoma patients, suggesting its utility as a prognostic biomarker.^169^ Lower CDKN2A expression is correlated with higher tumor grade and aggressive disease, reinforcing its relevance in glioma classification. Additionally, CDKN2A mRNA levels have been proposed as independent predictors of PFS and OS, supporting their potential clinical application in GBM management.^170^

Although targeting CDKN2A loss remains a challenge, its role in gliomagenesis underscores the need for therapeutic strategies aimed at restoring cell cycle control. Approaches such as CDK4/6 inhibitors, which compensate for p16INK4a loss, are being explored in GBM with CDKN2A deletion. Further research into CDKN2A-related pathways may provide new avenues for personalized GBM treatment, improving patient outcomes by integrating molecular diagnostics with targeted therapies.

#### PTEN

PTEN loss in GBM drives tumor progression and therapeutic resistance by dysregulating the PI3K/AKT/mTOR pathway, leading to uncontrolled cell growth, immune evasion, and an immunosuppressive TME. This is marked by increased PD-L1 expression, impaired T cell activation, and resistance to immune-mediated cell death, underscoring PTEN deficiency as a key factor in GBM immune escape.^171,172^ Additionally, PTEN loss alters the extracellular matrix (ECM) through the yes-associated protein 1 (YAP1) and lysyl oxidase (LOX) axes, facilitating angiogenesis and macrophage infiltration, which further supports tumor growth.^173^ Key mediators in this process include LOX and olfactomedin-like 3, which regulate macrophage and microglia recruitment. Inhibiting LOX in PTEN-deficient GBM enhances OLFML3 expression, promoting microglial infiltration via the nuclear factor kappa-light-chain-enhancer of activated B cells (NF-κB)-POZ/BTB and AT hook containing zinc finger 1 pathway. Targeting both macrophages and microglia through LOX inhibition and modulation of the CLOCK-OLFML3 axis, in combination with anti-PD-1 therapy, has demonstrated significant antitumor effects, highlighting a promising therapeutic strategy for GBM.^174^ This mechanism underscores the role of PTEN in modulating both cellular and microenvironmental factors in GBM progression.

The PTEN status of GBM has prognostic and therapeutic implications. Its loss is correlated with poor survival outcomes and resistance to standard treatments, including radiotherapy and chemotherapy. Given its central role in gliomagenesis, strategies aimed at restoring PTEN function or targeting downstream effectors, such as PI3K inhibitors or immune checkpoint blockade, are being explored as potential therapeutic approaches. Further research into PTEN-related pathways may enhance precision medicine strategies, offering new avenues for the effectiveness of GBM therapies.

### Tumor microenvironment

#### Tumor heterogenicity

GBM exhibits significant intratumor heterogeneity, driven by clonal evolution and cancer stem cell models. The clonal evolution model suggests that cumulative genetic and epigenetic alterations drive tumor progression, whereas the cancer stem cell model posits that a subset of tumor-initiating cells sustains growth and therapeutic resistance.^175^ These mechanisms contribute to glioma diversity, with tumor clones adapting to distinct brain regions, metabolic environments, and microarchitectures.^176^ TICs, a subset of GSCs, play a central role in GBM progression and resistance to therapy. They interact with TAMs and tumor-infiltrating lymphocytes (TILs), modulating immune evasion and tumor survival. TAMs constitute a significant proportion of the TME, promoting vascularization and resistance to immune clearance.^177,178^ The concept of interclonal cooperativity highlights how tumor subpopulations and stromal components create a supportive microenvironment that enhances tumor adaptability and malignancy.^179,180^ GBM rarely metastasizes outside the brain but frequently recurs locally. Whole-exome sequencing of recurrent GBM suggests that these tumors arise from residual primary tumor cells, supporting a model of evolutionary adaptation to treatment.^181^ Tumor heterogeneity influences differential treatment responses, particularly the expression of key biomarkers such as MGMT and RTKs.^182,183^ Studies have revealed that mixed tumor cell populations with distinct RTK amplifications, including EGFR, MET, and PDGFRA, contribute to therapeutic resistance.^184^

Cellular communication within the tumor niche occurs through EVs and tunneling nanotubes (TNTs), which facilitate the intercellular transfer of oncogenic signals, metabolic factors, and resistance-conferring molecules. TNTs, which are composed of F-actin extensions, allow tumor cells to exchange mitochondrial DNA and other critical components, driving tumor repopulation following therapy.^185^ Additionally, the role of Bruton’s tyrosine kinase (BTK) in GBM core cells suggests that BTK is a potential biomarker for distinguishing intratumor spatial heterogeneity, with implications for targeted therapies.^186^ The complexity of GBM heterogeneity presents challenges for treatment, necessitating strategies that target multiple tumor subpopulations and their interactions with the microenvironment. Overcoming therapy resistance requires a deeper understanding of GBM cell plasticity, metabolic adaptations, and immune modulation. Future therapeutic approaches must integrate precision medicine strategies that account for the dynamic evolution of GBM for better clinical outcomes.

#### GBM stem cells

GSCs exhibit key features, such as treatment resistance, low proliferative activity, and tumor recurrence potential. These stem-like cells are categorized into mesenchymal and proneural subtypes, with evidence suggesting that proneural GSCs can transition into mesenchymal GSCs upon recurrence, contributing to GBM heterogeneity and therapeutic resistance.^187,188^ GSCs play crucial roles in tumor invasion and recurrence by migrating along the vasculature and white matter tracts, where they utilize cadherins, integrins, and MMPs.^189,190^ The invasive potential of these cells is driven by upregulated signaling pathways, including L1CAM, ephrin-B2,^191^ and epithelial‒mesenchymal transition (EMT)-associated factors such as twist-related protein 1 (TWIST1), SOX2, and signal transducer and activator of transcription 3 (STAT3).^192^ Additionally, GSCs exhibit heightened DNA repair capabilities, relying on Rad3-related kinase (ATR), ATM, poly(ADP‒ribose) polymerase 1 (PARP1), and other repair proteins, which contribute to their resistance to radiation and chemotherapy.^193^ Replication stress in GSCs, associated with prolonged transcription of long neural genes, results in increased reliance on DNA damage response pathways, including ATR and checkpoint kinase 1 (Chk1) activation. These adaptations increase GSC survival under genotoxic stress, suggesting potential therapeutic targets.^194^

GSCs modulate the TME by promoting immunosuppressive mechanisms. They induce M2 differentiation in glioma-associated macrophages (GAMs) through periostin secretion and IL-10 signaling, contributing to immune evasion.^195^ Additionally, GSC-derived pericytes support angiogenesis, promoting vascular abnormalities and BBB disruption.^190^ The Wnt and Sonic hedgehog signaling pathways maintain GSC self-renewal and therapy resistance. Aberrant Wnt activation, influenced by FAT atypical cadherin 1 (FAT1) mutations, enhances tumor progression, whereas sonic hedgehog signaling promotes Nanog expression and drug efflux transporter activity, further increasing chemoresistance.^196^ GSCs contribute to GBM relapse by resisting conventional therapies. The ability of these cells to persist in a quiescent state, evade apoptosis, and promote tumor angiogenesis underscores the need for targeted strategies.^197^ Future research should focus on disrupting GSC-specific pathways, enhancing tumor immunogenicity, and integrating novel therapies to support improved disease management in GBM.

#### Angiogenesis

Angiogenesis is a key process in GBM progression and is driven by multiple growth factors and signaling pathways. VEGF is a primary regulator, and its expression increases with tumor grade, promoting vascular proliferation and tumor progression.^198^ VEGFR-1 and VEGFR-2 activation play distinct roles in GBM initiation and malignancy.^199^ The overexpression of VEGF and VEGFR-1 in low-grade astrocytomas is correlated with poor prognosis, indicating their potential as prognostic biomarkers.^200^ Angiogenic factors in GBM are regulated by oncogene activation, tumor suppressor loss, and hypoxia. FGFR signaling, which is mediated by FGF ligands, supports endothelial migration, proliferation, and angiogenesis through PI3K/AKT/mTOR activation.^201,202^ FGF-2 enhances ECM remodeling and cooperates with VEGF and PDGF to promote neovascularization, indicating their combined role in tumor vascularization.^203^ The HGF/c-MET axis further drives tumor growth, invasion, and angiogenesis, with increased expression linked to increased tumor grade and poor prognosis.^204^ The inhibition of MET and VEGF has synergistic effects on suppressing tumor growth, suggesting a viable therapeutic strategy.^205^

Angiopoietins (Ang-1, Ang-2, Ang-4) contribute to GBM vascularization. Ang-2 disrupts vessel stability, promoting neovascularization, whereas Ang-4 enhances tumor angiogenesis.^206^ The Tie-2 receptor, which is expressed in GBM, regulates VEGF expression, and dual inhibition of VEGF and Ang-2 improves survival outcomes.^207^ TGF-β modulates angiogenesis through context-dependent effects, promoting VEGF, FGF, and PDGF expression while also inducing EMT in GBM-derived endothelial cells.^208^ MMPs degrade the endothelial basement membrane, facilitating angiogenic switching. MMP-9-mediated VEGF release contributes to tumor vascularization.^209,210^ Targeting these angiogenic pathways offers potential therapeutic avenues, with combination therapies addressing VEGF resistance through simultaneous inhibition of complementary pathways. Further research is needed to refine antiangiogenic strategies and improve patient outcomes in GBM treatment.

#### Autophagy

Autophagy plays a complex role in GBM, influencing tumor progression, treatment response, and patient prognosis.^211^ While it contributes to tumor survival by providing metabolic substrates under hypoxic conditions, excessive autophagy can also lead to cell death and suppress invasion. In GBM, the expression of autophagy-related genes such as autophagy-related (*ATG*) *7*, *ATG13*, and *UNC-51*, such as autophagy-activating kinase 1 (ULK1), is often downregulated, impairing the autophagic capacity of tumors as they progress.^212^ However, high levels of autophagic markers such as microtubule-associated protein 1 light chain 3 (LC3) and Beclin-1 (BECN1) correlate with better patient outcomes, suggesting a potential tumor-suppressive function in certain contexts.^213^ The interplay between autophagy and key oncogenic pathways further complicates its role. The mTOR pathway inhibits autophagy and supports GSC proliferation, whereas autophagy suppression enhances EGFR overexpression, promoting tumor progression.^213^ Additionally, miR-224-3p downregulation under hypoxic GBM conditions reduces autophagy by targeting ATG5 and FAK family kinase-interacting protein of 200 kDa (FIP200), linking miRNA regulation to tumor metabolism.^214^ Conversely, the upregulation of Bcl-2 interacting protein 3 (BNIP3) under hypoxic conditions promotes autophagy, supporting GBM cell survival.^215^

Autophagy also regulates EMT and treatment resistance in GBM. It suppresses tumor invasion by increasing N-cadherin membrane localization and degrading EMT transcription factors such as Snail.^216^ However, stress-induced autophagy can also contribute to therapy resistance, enhancing GBM cell survival following radiation or chemotherapy. For example, autophagy promotes resistance to TMZ by maintaining GSCs, while targeting ATG4C has been shown to increase TMZ sensitivity.^217^ Autophagy-related proteins such as p62 and transcription factor EB (TFEB) are linked to GBM prognosis, with high p62 expression correlating with PS and tumor recurrence.^218^ In contrast, BECN-1 expression is associated with IDH1 mutation and 1p/19q codeletion, suggesting a context-dependent impact on GBM biology.^219^ Overall, autophagy represents a double-edged sword in GBM, with both tumor-promoting and tumor-suppressing effects. Targeting autophagic pathways may offer novel therapeutic strategies, but a deeper understanding of their dual role is necessary to optimize treatment approaches.

#### Hypoxia

Hypoxia, regulated primarily by hypoxia-inducible factor 1 (HIF-1), plays a critical role in GBM progression, influencing angiogenesis, metabolic reprogramming, immunosuppression, and therapy resistance.^220^ HIF-1 expression is strongly associated with increased tumor grade and poor prognosis, as it drives the adaptation of GBM cells to hypoxic stress.^221^ A meta-analysis confirmed that elevated HIF-1 levels correlate with reduced OS in GBM patients.^222^ However, additional hypoxia-related markers, such as carbonic anhydrase IX (CA9) and osteopontin, have emerged as potentially superior indicators of tumor aggressiveness.^223^ HIF-1 plays a pivotal role in the metabolic shift to aerobic glycolysis, known as the Warburg effect, facilitating glucose conversion to lactate despite sufficient oxygen availability. This metabolic reprogramming supports tumor proliferation and enhances malignancy by promoting lactate production and extracellular acidification, which in turn stabilizes HIF-1α and sustains tumor hypoxia.^224^ Additionally, HIF-1 regulates glutamine metabolism, shifting it toward α-ketoglutarate (α-KG) production, which fuels fatty acid biosynthesis and prevents lipotoxicity.^225^

In addition to its role in metabolism, HIF-1 contributes to GBM invasiveness by promoting EMT through the activation of the Snail and ZEB1 transcription factors, downregulating E-cadherin, and enhancing ECM remodeling.^226^ HIF-1 also upregulates matrix MMPs (MMP-2, MMP-9, and MMP-14), cathepsins, and fibronectin, facilitating basement membrane degradation and tumor cell migration.^227^ Furthermore, it fosters an immunosuppressive microenvironment by increasing lactate production and adenosine accumulation, which suppress T cell function and enhance regulatory T cell (Treg) activity. HIF-1 plays a key role in treatment resistance, particularly in radiotherapy, by activating antioxidant systems that mitigate ROS-induced DNA damage.^228^ It stabilizes DNA strand breaks, promoting survival under oxidative stress. Additionally, HIF-1 supports the maintenance of GSCs by upregulating stemness-associated genes such as KLF4, MYC, OCT4, SOX2, and NANOG, sustaining their self-renewal and resistance to conventional therapies.^229,230^

Therapeutically, targeting HIF-1α has shown promise in sensitizing GBM cells to TMZ, particularly in patients with MGMT promoter methylation. By decreasing MGMT expression, HIF-1 inhibition enhances the cytotoxic effects of alkylating agents, offering a potential strategy to improve patient outcomes. Given the extensive role of HIF-1 in GBM progression, metabolic adaptation, and therapy resistance, it remains a critical target for novel therapeutic interventions aimed at disrupting tumor survival mechanisms in hypoxic microenvironments.

#### Metabolic reprogramming in GBM

The metabolic characterization of GBM, particularly in relation to IDH1/2 mutations, provides critical insights into tumor adaptation and progression. IDH mutant GBMs exhibit distinct metabolic alterations, including the accumulation of the oncometabolite 2-HG, which inhibits α-KG-dependent dioxygenases and disrupts DNA repair and cellular differentiation. This metabolic reprogramming contributes to tumor maintenance and therapeutic vulnerability, particularly in the context of targeting NAD^+^ metabolism and PARP inhibitors. GBMs demonstrate remarkable metabolic plasticity, relying on glucose metabolism while adapting to alternative carbon sources under stress. Increased expression of glucose transporters (GLUT1/3) and hexokinase 2 (HK2), which are regulated by HIF-1α and HIF-2α, supports glycolysis, even under hypoxic conditions. Loss of PTEN function further enhances glycolysis via AKT1 activation, stabilizing phosphofructokinase (PFKP).^231,232^ Additionally, MYC-driven metabolic reprogramming promotes a shift toward aerobic glycolysis and lactate production, limiting mitochondrial oxidative phosphorylation.

Lipid metabolism plays a crucial role in GBM heterogeneity, with GSCs displaying distinct metabolic dependencies. GSCs utilize fatty acids and ketone bodies for energy, allowing survival in nutrient-limited environments.^233^ The activation of EGFR-PI3K-AKT signaling regulates sterol regulatory element-binding protein-1 (SREBP-1), driving lipid biosynthesis and promoting tumor progression. Pseudopalisading regions in GBM accumulate fatty acids via FABP3/7, supporting tumor invasion and angiogenesis.^234^ Emerging evidence suggests that targeting fatty acid synthase (FASN)^235^ and polyunsaturated fatty acid (PUFA) synthesis may provide therapeutic benefits.^236^ Nitrogen metabolism is also altered in GBM, with dysregulated glutamine and cysteine metabolism contributing to tumor growth and resistance. Increased glutaminase activity and amino acid transport (SLC7A11) promote glutathione synthesis, enhancing redox homeostasis and therapy resistance.^237^ Targeting glutaminase with inhibitors such as telaglenastat (CB-839) in combination with radiotherapy and TMZ is a promising strategy that is currently under clinical investigation.^238,239^

These metabolic adaptations highlight potential therapeutic targets in GBM. Inhibiting PTEN loss-driven glucose metabolism, disrupting PUFA biosynthesis, and blocking 2-HG production in IDH mutant tumors represent viable strategies. Understanding the metabolic vulnerabilities of GBM patients offers new opportunities for precision medicine, emphasizing the need for continued research into metabolic-targeted therapies to increase the survival and quality of life of GBM patients.

#### Impact of immune cells

TAMs play crucial roles in GBM progression by promoting tumor growth, immune evasion, and therapy resistance. TAMs secrete factors such as EGF, TGF-β, and MMP-2, which enhance GBM proliferation and invasiveness.^240^ They also drive GSC renewal via cytokines such as IL-6 and IL-12.^241^ Additionally, TAMs support an immunosuppressive TME by recruiting Tregs and suppressing effector T cells and natural killer (NK) cells through chemokines such as chemokine (CC-motif) ligand 2 (CCL2) and C-C motif chemokine ligand 22 (CCL22).^242^ The overexpression of indoleamine 2,3-dioxygenase 1 (IDO1) and TDO2 in GBM leads to the production of L-kynurenine, which interacts with the aryl hydrocarbon receptor (AHR) on TAMs, further inhibiting immune responses.^243^ Recent studies have highlighted how TAM infiltration reshapes GBM transcriptional profiles, promoting mesenchymal transformation and therapy resistance.^244^ Neutrophils also contribute to GBM progression, with increased peripheral neutrophil-to-lymphocyte ratios correlated with poor prognosis.^245^ GBM cells recruit neutrophils through IL-8 and granulocyte–macrophage colony-stimulating factor (GM-CSF) signaling, extending their survival and promoting tumor invasiveness. The receptor for advanced glycation end products (RAGE) on GBM enhances neutrophil infiltration and NF-κB activation, leading to increased tumor-supportive inflammation.^246^ While early-stage neutrophils can exert antitumor effects via ROS production, their tumor-promoting functions dominate in advanced disease. Notably, a subset of tumor-associated neutrophils (TANs) can differentiate into antigen-presenting cells (APCs), activating T cells and counteracting tumor progression.^247^

MDSCs contribute to GBM immune evasion by inhibiting T cell activation, NK-cell function, and antigen-presenting cells. Tumor-derived cytokines such as IL-6, IL-8, IL-10, CSF-1, CCL2, CXCL2, PGE2 and TGF-β promote MDSC expansion and recruitment,^248,249^ whereas hypoxia shifts MDSC metabolism toward fatty acid oxidation,^250^ reinforcing their immunosuppressive properties. MDSCs release nitric oxide and arginase 1 (Arg1), depleting essential metabolites and suppressing T cell proliferation.^251^ The ability of MDSCs to induce Tregs and impair cytotoxic immune responses makes them key targets for immune-modulatory therapies. T cells play diverse roles in GBM and are influenced by tumor genetics and immune interactions. CD8^+^ cytotoxic T lymphocytes (CTLs) can induce tumor cell apoptosis, but their infiltration and activation vary among glioma subtypes. LGGs with NF1 mutations show greater T cell infiltration, whereas mesenchymal GBMs demonstrate substantial but often ineffective T cell presence. GBM exploits T cell regulatory mechanisms, such as PD-L1 upregulation and TGF-β signaling, to evade immune surveillance. While T cell infiltration has been linked to improved survival in some GBM patients, tumor-driven immunosuppression often limits its effectiveness.^252,253^

T cell exhaustion is a key immunosuppressive mechanism in GBM that is characterized by diminished effector function and elevated inhibitory receptor expression. Unlike memory T cell differentiation in acute immune responses, exhausted T cells in GBM fail to sustain long-term antitumor immunity due to persistent antigen exposure, metabolic stress, and an immunosuppressive TME.^254^ The TME of GBM suppresses T cell activation through inhibitory cytokines such as TGF-β, IL-10, and IL-35,^255,256^ along with an abundance of regulatory immune cells, including Tregs. MDSCs, and TAMs. These factors contribute to an immune-desert phenotype, limiting T cell infiltration and function.^257^ Chronic T cell receptor stimulation and nutrient deprivation further promote exhaustion, particularly in CD8^+^ T effector memory (Tem) cells, which are critical for long-term immune surveillance. The high expression of PD-L1 on MDSCs, immature dendritic cells (DCs), and plasmacytoid DCs reinforces immune suppression by impairing T cell activation.^258,259^ The accumulation of GAMs, comprising up to 30–50% of the GBM tumor mass, further skews the immune landscape.^260^ While M1 macrophages exhibit antitumor properties, M2-polarized macrophages secrete IL-10 and PD-L1, enhancing T cell dysfunction and promoting tumor progression.^261^

Targeting myeloid cells has emerged as a strategy to restore T cell function. CSF-1R inhibitors, aimed at blocking M2-macrophage polarization, have shown promise in preclinical models but have failed to improve survival in clinical trials.^262^ In contrast, CD47-blocking antibodies have demonstrated potential in reprogramming macrophages toward the M1 phenotype, enhancing CD8^+^ Tem cell-mediated immunity.^263^ Advanced epigenetic and single-cell transcriptomic analyses revealed that GAMs exhibit plasticity, adapting to environmental stimuli to either suppress or enhance immune responses. Understanding these dynamic interactions between T cells and myeloid populations may provide novel therapeutic avenues for reversing T cell exhaustion and overcoming immune evasion in GBM. Future research should prioritize strategies that modulate GAM polarization, suppress MDSCs, and reinvigorate exhausted T cells to strengthen antitumor immunity and improve therapeutic outcomes.

### Dysregulated signaling pathways in GBM progression

**Fig. 5:**
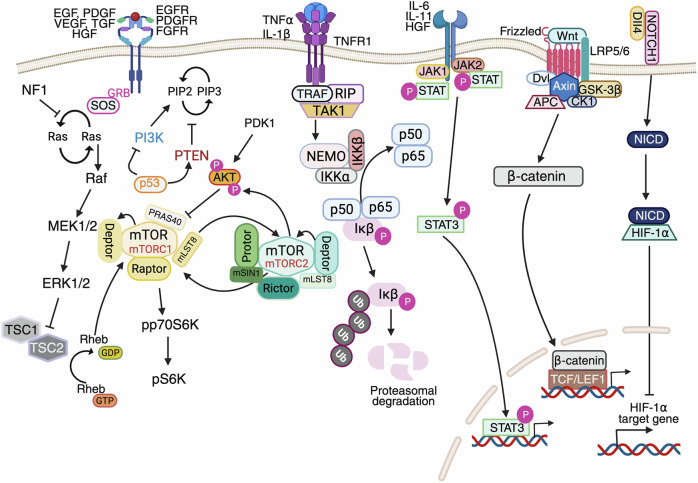
Deregulated molecular signaling pathways and crosstalk in glioblastoma. This illustration highlights key oncogenic signaling pathways and their interconnected roles in glioblastoma (GBM) pathogenesis. Dysregulated pathways such as the PI3K/AKT/mTOR, MAPK/ERK, p53, NF-κB, JAK/STAT, β-catenin, and Notch pathways collectively drive tumor progression, survival, and resistance to therapy. NF-κB activation, triggered by TNFα/TNFR1 and the IKK complex, integrates inflammatory signaling, whereas cytokine-mediated activation of the JAK/STAT pathway promotes the transcription of survival genes. The Wnt/β-catenin and Notch pathways further support stemness, angiogenesis, and immune modulation. The convergence and crosstalk among these pathways contribute to the complexity and aggressiveness of GBM

Dysregulated signaling pathways are central to GBM pathogenesis and promote tumor growth, invasion, and resistance to therapy. Aberrant activation of key molecular and immune signaling pathways leads to uncontrolled cell proliferation, enhanced tumor cell survival, and maintenance of cancer stem-like cells. These alterations not only promote aggressive tumor growth but also contribute to resistance to standard therapies by enhancing DNA repair mechanisms and evasion of apoptosis. Targeting these dysregulated pathways represents a fundamental approach for therapeutic interventions and disruption of the TME, offering the potential to improve GBM treatment outcomes (Fig. 5).

#### Molecular signaling pathways

##### PI3K/AKT/mTOR

The PI3K/AKT/mTOR pathway is a central regulator of GBM progression and influences cell survival, proliferation, and metabolic adaptation. Its activation, triggered by tyrosine kinase receptors, Ras, and integrins, promotes tumor growth and treatment resistance.^10^ Dysregulation of this pathway is observed in approximately 70% of GBM patients and is correlated with poor prognosis, highlighting its relevance as a therapeutic target. PTEN, a critical tumor suppressor, negatively regulates the PI3K/AKT/mTOR pathway, and its loss further exacerbates GBM aggressiveness.^264^ The inhibition of mTOR with rapamycin has demonstrated promising effects in vitro, but clinical trials have shown limited efficacy,^265^ suggesting the need for combination therapies. Studies using orthotopic GBM models highlight a strong correlation between AKT activation and increased tumor growth, invasion, and resistance to therapy,^266^ reinforcing its role as a therapeutic target. However, AKT also plays a role in astrocytic differentiation, adding complexity to its function in GBM.

mTOR, a key effector of PI3K/AKT signaling, is implicated in cell survival, metabolic reprogramming, and GBM cell proliferation.^267^ Both mTORC1 and mTORC2 contribute to GBM progression, with mTORC1 promoting glycolysis via HIF-1 activation and mTORC2 enhancing tumor cell motility through RICTOR overexpression.^10,268^ Additionally, alternative activation pathways, such as PKCα-mediated EGFR-mTOR signaling, indicate that multiple regulatory inputs sustain mTOR activity in GBM.^269^ The complexity of PI3K/AKT/mTOR signaling in GBM necessitates a multifaceted therapeutic approach. Combination strategies targeting mTOR, PI3K, and associated compensatory mechanisms may enhance treatment efficacy. Future research should focus on identifying resistance pathways and refining targeted therapies to improve patient outcomes.

##### NF-κB

NF-κB activation is a hallmark of GBM and is driven by oncogenic pathways such as the EGFR and PDGFR signaling pathways, as well as genetic alterations in PTEN, NF1, and ARF. The inflammatory TME further amplifies NF-κB activity, reinforcing its role in tumor progression. Additionally, NF-κB signaling is sustained by epithelial V-like antigen 1 (Eva1), which maintains GSC characteristics through the regulation of stemness-associated genes.^270,271^ In addition to its role in tumor maintenance, NF-κB promotes the mesenchymal phenotype of GBM by activating key transcription factors, including STAT3, CCAAT/enhancer-binding protein β (C/EBPβ), and TAZ. This process is reinforced by a feedback loop involving fibroblast growth factor-inducible 14, further enhancing GBM invasion.^272^ NF-κB also plays a critical role in angiogenesis by upregulating VEGF and IL-8, contributing to tumor vascularization.^273^ Metabolic reprogramming in GBM is influenced by NF-κB, particularly through its regulation of pyruvate kinase M2, a key glycolytic enzyme upregulated in response to EGFR signaling.^274^ Moreover, NF-κB is implicated in therapy resistance, enhancing DNA damage repair to promote radioresistance and regulate MGMT expression, contributing to chemoresistance.^275^ Given its multifaceted role in tumor invasion, angiogenesis, metabolism, and therapy resistance, NF-κB represents a promising therapeutic target in GBM. Future strategies should focus on disrupting NF-κB signaling to increase treatment sensitivity and inhibit tumor progression.

##### STAT3

STAT3 activation in GBM is driven by multiple receptor tyrosine kinase pathways, including the EGFR, PDGFR, and c-MET pathways, along with the loss of negative regulators such as protein tyrosine phosphatases, suppressors of cytokine signaling, and protein inhibitors of activated STAT3.^276^ This sustained activation promotes tumor growth by upregulating the expression of oncogenic transcription factors such as c-Myc, cyclin D1, and Bcl-xl. STAT3 also plays a crucial role in maintaining GSC properties through its regulation of SOX2, OLIG2, OCT4, and NANOG,^277^ reinforcing the self-renewal and invasive capabilities of tumors. Additionally, STAT3 facilitates hypoxia-driven angiogenesis and tumor migration by modulating HIF-1, VEGF, MMP2, and TWIST.^278^ In addition to promoting tumor proliferation, STAT3 contributes to GBM aggressiveness by promoting EGFRvIII-mediated invasion through JAK2/STAT3 signaling and stabilizing focal adhesion complexes.^279^ It also establishes an immunosuppressive microenvironment, enabling tumor immune evasion.^280^ Importantly, STAT3 is a major player in therapy resistance and regulates MGMT expression, conferring TMZ resistance, interacting with FOXM1 to promote radioresistance, and mediating resistance to anti-VEGF and MET inhibitors.^281,282^ Interestingly, the role of STAT3 in GBM is context dependent. In PTEN-deficient tumors, STAT3 may act as a tumor suppressor, inhibiting proliferation and invasion.^283^ This complexity underscores the need for a nuanced therapeutic approach targeting STAT3. Given its involvement in multiple oncogenic processes and therapy resistance, STAT3 represents a key target for improving GBM treatment outcomes.

##### Wnt/β-catenin

The Wnt/β-catenin pathway plays a key role in glioma progression by maintaining tumor stem cell populations, inhibiting differentiation, and promoting invasion. While essential for normal brain development, its dysregulation in GBM is linked to increased malignancy and poor prognosis.^284^ Aberrant activation of this pathway contributes to treatment resistance and tumor aggressiveness, making it a critical therapeutic target. Epigenetic alterations further regulate Wnt signaling in GBMs. Hypermethylation-mediated silencing of Wnt inhibitors is a common event, particularly in astrocytic gliomas, that influences tumor progression. Distinct patterns of Wnt pathway gene hypermethylation in primary and secondary GBMs suggest subtype-specific regulatory differences.^285^ Studies have also reported that the overexpression of Wnt ligands (Wnt2, Wnt3a, and Wnt5a), Frizzled receptors, and β-catenin in GBM correlates with tumor grade and poor patient outcomes. Knockdown of Wnt2 and β-catenin has been shown to suppress tumor growth, reduce invasion, and induce apoptosis in tumor cells.^286^ Targeting the Wnt pathway offers a promising therapeutic strategy for GBMs, with potential applications in overcoming radioresistance and chemoresistance. Further research into subtype-specific alterations and regulatory mechanisms is essential for the development of effective Wnt-targeted therapies tailored to GBM heterogeneity.

##### IGFR

Dysregulated IGF signaling contributes to GBM progression and therapy resistance. Elevated IGF ligands and IGF1R overexpression are linked to increased tumor growth, poor prognosis, and a reduced response to TMZ therapy.^287^ IGF1R activates key oncogenic pathways, including the PI3K/AKT and RAS/RAF/MAPK pathways, with ligand-driven activation playing a primary role in tumor cell proliferation.^288^ Targeting IGF signaling has shown therapeutic potential. IGF1R inhibitors such as IMC-A12 and picropodophyllin effectively suppress GBM growth in preclinical models, reducing tumor proliferation and angiogenesis.^288,289^ These findings highlight IGF1R as a promising therapeutic target, warranting further investigation into its role in chemoresistance and the potential benefits of combination therapies integrating IGF1R inhibitors with standard GBM treatments.

##### NOTCH

Notch signaling plays a complex role in GBM, exhibiting both tumor-promoting and tumor-suppressive effects depending on the molecular and cellular context.^290^ While Notch1 overexpression is correlated with PS in some cases, it is also linked to better prognosis in specific GBM subtypes. Notch pathway activity varies across tumor regions, with higher expression in peritumoral GSCs than in the tumor core, suggesting a role in maintaining stemness and therapeutic resistance. Notch2 and Notch4 also influence GBM differentiation and aggressiveness, reinforcing the impact of these pathways on tumor heterogeneity.^291,292^ Hypoxia-driven Notch activation further promotes tumor progression by increasing the expression of TRPC6, which stimulates NFAT activity and GBM cell proliferation.^293^ The interplay between Notch and STAT3 signaling in mesenchymal GBMs suggests a cooperative mechanism in driving tumor aggressiveness.^20,294^ Additionally, Notch activation in GSCs contributes to perivascular niche remodeling and angiogenesis, supporting tumor vascularization and therapy resistance.^295^ Targeting Notch signaling represents a potential therapeutic avenue, particularly in combination with hypoxia or angiogenesis inhibitors. Inhibiting Notch1 has shown promise in reducing tumor hypoxia and sensitizing GBM to radiotherapy.^296^ The convergence of the Notch pathway with the PDGF and nitric oxide signaling pathways highlights additional regulatory mechanisms that sustain GSCs and GBM progression.^295^ Understanding the context-dependent role of Notch in GBM could facilitate the development of more precise therapeutic interventions and prognostic markers for patient stratification.

##### Hedgehog pathway

The Hedgehog (Hh) signaling pathway plays a critical role in GBM, influencing tumor growth, stemness, angiogenesis, and treatment resistance. Its key effectors, particularly GLI family zinc finger 1 (GLI1), regulate cell proliferation through interactions with p53 and are essential for GSC maintenance. A truncated GLI1 variant (tGLI1), detected in most GBM cases but absent in normal brain cells, promotes tumor progression by activating genes not regulated by canonical GLI1, including VEGFR1, VEGF-C, TEM7, HPSE, CD24, and CD44.^297^ tGLI1 drives GBM invasion by upregulating CD24 and contributes to angiogenesis through VEGF signaling. It also induces the mesenchymal GBM subtype by increasing the expression of CD44, a key marker of mesenchymal GSCs.^297,298^ Additionally, tGLI1 enhances EMT by modulating miRNAs such as miR-21, miR-128, and miR-200. Recent findings revealed that metabotropic glutamate receptor 4 (mGluR4) negatively regulates GLI1, suppressing proliferation and inducing apoptosis, suggesting a potential therapeutic target.^299^

In addition to its role in tumor growth, GLI1 contributes to treatment resistance. It enhances the replicative potential of GBM cells by activating TERT and promotes resistance to TMZ and radiotherapy by upregulating MGMT expression.^300^ Given its multifaceted role in GBM progression, targeting aberrant Hh signaling—particularly tGLI1—may offer promising therapeutic strategies to counteract metastasis and treatment resistance. Further research into Hh pathway dysregulation could pave the way for more effective, targeted therapies for GBM management.

##### Ceramide signaling

Acid ceramidase (ASAH1) plays a critical role in GBM metabolism by converting ceramides into sphingosine and free fatty acids. This shift promotes the production of sphingosine-1-phosphate (S1P), a key driver of GBM survival, proliferation, and resistance to apoptosis.^301^ Elevated ASAH1 expression in GBM has been linked to increased tumor cell viability, migration, and recurrence, highlighting its potential as a prognostic biomarker. Additionally, ASAH1 secretion into interstitial tissues facilitates tumor progression by modifying the surrounding microenvironment. Targeting ASAH1 represents a promising therapeutic strategy. Inhibitors of ASAH1 have demonstrated efficacy in preclinical studies, reducing tumor cell growth and potentially overcoming resistance to standard treatments such as TMZ.^302^ While no clinical trials currently focus on ceramide signaling in GBM, further research into ASAH1 inhibition could provide novel approaches to restoring ceramide-induced apoptosis and improving patient outcomes. Expanding our understanding of the role of ASAH1 in GBM progression may lead to the development of targeted therapies that disrupt its protumorigenic effects.

##### TEAD transcription factors

TEA domain (TEAD) transcription factors, in coordination with YAP1 and TAZ, play crucial roles in GBM pathogenesis. The TAZ-TEAD2 complex drives mesenchymal differentiation by binding to mesenchymal gene promoters, whereas TEAD1 and TEAD4 contribute to various tumorigenic processes.^303^ TEAD1 enhances EGFR-mediated c-Myc expression and regulates migration through aquaporin 4 (AQP4).^79^ TEAD4, in partnership with TAZ, regulates cell proliferation, apoptosis, invasion, and EMT by modulating key genes such as cyclin D1, KI67, c-Myc, Bcl2, MMP-9, vimentin, and N-cadherin.^304^ These findings highlight the TEAD family as critical mediators of GBM progression, with implications for tumor aggressiveness and treatment resistance. Targeting TEAD signaling, particularly its interaction with YAP1 and TAZ, may offer new therapeutic strategies to disrupt mesenchymal transition and GBM proliferation, paving the way for improved patient outcomes.

##### C/EBPβ

C/EBPβ is a key transcription factor implicated in GBM pathogenesis, particularly in the mesenchymal subtype. Its activation is linked to KLHL9 deletions and EGFR signaling, positioning it as a central player in tumor progression.^305^ In conjunction with STAT3, C/EBPβ drives mesenchymal differentiation, enhancing GBM cell invasion, proliferation, and survival.^192^ C/EBPβ contributes to GBM pathobiology by regulating DNA damage responses and inducing genes associated with invasion and metastasis. It also promotes angiogenesis via IL-6 and IL-8 and fosters an immunosuppressive TME by upregulating tryptophan-2,3-dioxygenase (TDO2), which enhances kynurenine production. Additionally, C/EBPβ modulates antioxidative defense mechanisms by regulating NAD(P)H quinone oxidoreductase 1 (NQO1) and glutathione S-transferase pi 1 (GSTP1), protecting GBM cells from oxidative stress.^306,307^ Given its multifaceted role in GBM progression, targeting C/EBPβ presents a promising therapeutic strategy. Inhibiting its activity could mitigate tumor aggressiveness, disrupt immunosuppression, and enhance treatment responses, making it a viable candidate for future GBM therapies.

##### c-Myc

c-Myc is a key transcription factor in GBM that influences tumor growth, stemness, invasion, and resistance to therapy. Its dysregulation, driven by gene amplification and epigenetic modifications, promotes GBM cell proliferation and mitotic activity. Additionally, c-Myc enhances tumor vascularization by upregulating miR-9 and facilitates GBM cell invasion through RhoA activation.^308,309^ In addition to its role in tumor progression, c-Myc is a central regulator of GBM metabolism, driving a shift toward glycolysis to sustain energy production under hypoxic conditions. Importantly, it contributes to resistance against radiation and TMZ by upregulating DNA repair proteins such as Nibrin (NBS1) and Reversionless 3-like (REV3L), enabling tumor cells to withstand genotoxic stress.^310^ Given its broad oncogenic influence, targeting c-Myc represents a promising therapeutic strategy in GBM. Inhibiting its activity could disrupt tumor metabolism, angiogenesis, and therapy resistance, providing a potential approach to improve treatment efficacy in this aggressive malignancy.

##### PKC

Dysregulated protein kinase C (PKC) signaling contributes to GBM growth, proliferation, and invasion. Elevated PKC activity is correlated with aggressive tumor behavior, with specific isoforms playing distinct roles in GBM progression.^311^ PKCα drives mitogenic and prosurvival signaling, enhances GBM migration via the ERK/NF-κB pathways, and regulates FGF expression for tumor cell proliferation. Other isoforms, such as PKCε and PKCη, facilitate cell adhesion, motility, and survival. Given the multifaceted role of PKC in glioma biology, targeting its isoforms offers a potential therapeutic approach to disrupt tumor growth and invasion.^312,313^ Inhibiting PKC-mediated pathways could improve treatment efficacy, providing a rationale for further exploration of the use of PKC inhibitors in GBM therapy.

#### Immune signaling pathways

##### CSF-1R

**Fig. 6:**
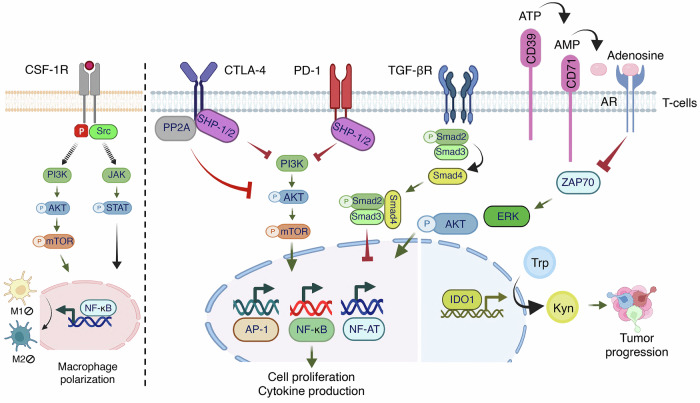
Key immune signaling pathways regulating tumor-associated immunosuppression. In macrophages, CSF-1 or IL-34 binds to the CSF-1 receptor, inducing rapid dimerization and autophosphorylation of tyrosine residues. This activation triggers downstream signaling through the PI3K/AKT and JAK/STAT pathways, regulating macrophage polarization. CTLA-4, which is expressed on activated T cells, binds to CD80/CD86 on APCs. Upon engagement, CTLA-4 signaling dephosphorylates TCR signaling components, inhibiting CD3 and ZAP70 activation and suppressing the RAS signaling pathway. CTLA-4 signaling disrupts AKT phosphorylation, negatively regulating the cell cycle and suppressing key transcription factors such as NF-κB, AP-1, and NF-AT. PD-1 interacts with its ligands, leading to the phosphorylation of two tyrosine residues on its cytoplasmic tail. This phosphorylation recruits SHP-1 and SHP-2 to the ITSM motif, inhibiting the PI3K/AKT/mTOR pathway, reducing metabolic activity, and promoting T cell exhaustion. In the case of TGF-βR2 ligand binding, the receptor activates and facilitates PI3K and AKT signaling through physical interaction with the PI3K subunit. This cascade leads to mTOR kinase activation, which drives translational responses. Collectively, these signaling pathways induce IDO1 activation, which converts tryptophan to kynurenine, thereby enhancing tumor immune evasion through immune suppression. The CD39/CD73 pathway hydrolyzes extracellular ATP into adenosine, an immunosuppressive metabolite. Adenosine prevents tyrosine phosphorylation of ZAP70, AKT, and ERK1/2 in naive αCD3/CD28-stimulated CD8^+^ T cells, impairing their activation

*The* CSF-1R pathway critically influences macrophage polarization and contributes significantly to immunosuppression and tumor progression in GBM. CSF-1R, which is predominantly expressed on macrophages and microglia, binds to CSF-1, driving the activation, proliferation, and survival of these immune cells. Within the GBM microenvironment, elevated CSF-1 signaling promotes the recruitment and polarization of macrophages toward an immunosuppressive, protumorigenic M2 phenotype, increasing tumor growth, invasion, and immune evasion^314^ (Fig. 6). Targeting the CSF-1R pathway has demonstrated potential in shifting macrophage polarization from an M2-like immunosuppressive phenotype toward an M1-like proinflammatory phenotype, thereby facilitating antitumor responses. Preclinical models of GBM have shown that CSF-1R inhibition can significantly reduce TAMs, resulting in reduced tumor growth and improved survival outcomes. Additionally, combining CSF-1R blockade with ICIs has shown enhanced therapeutic efficacy by overcoming macrophage-mediated immunosuppression.^315^ Thus, targeting CSF-1R signaling represents a promising strategy for GBM immunotherapy.

##### CTLA-4

Cytotoxic T-lymphocyte-associated protein 4 (CTLA-4) represents another pivotal checkpoint molecule that negatively regulates T cell activation. Tregs in GBM express high levels of CTLA-4, which competitively bind to B7 ligands (CD80/CD86) on antigen-presenting cells. Upon binding to its ligands, CD80 or CD86, CTLA-4 recruits phosphatases such as SHP-2 and protein phosphatase 2A (PP2A) to its cytoplasmic domain. These phosphatases dephosphorylate key signaling molecules downstream of the T cell receptor (TCR), leading to attenuation of the PI3K/AKT signaling pathway.^315^ This interaction significantly reduces the availability of essential costimulatory signals for effector T cells, leading to diminished activation, proliferation, and antitumor function of these cells (Fig. 6). Thus, CTLA-4 engagement reinforces the immunosuppressive TME, promoting tumor progression and resistance to checkpoint blockade therapies.^316^

##### PD-1/PD-L1

The PD-1/PD-L1 axis is a critical immune checkpoint pathway exploited by GBM cells to evade immune surveillance. In addition to MDSCs, tumor cells upregulate PD-L1 expression, which interacts with PD-1 on activated T cells, leading to T cell exhaustion and reduced antitumor activity. Upon engagement of PD-1 by its ligand PD-L1, the immunoreceptor tyrosine-based inhibitory motif (ITIM) and immunoreceptor tyrosine-based switch motif (ITSM) within the PD-1 cytoplasmic domain become phosphorylated. This phosphorylation recruits SH2 domain-containing phosphatases, specifically SHP-1 and SHP-2, which dephosphorylate key signaling molecules downstream of the TCR, leading to attenuation of T cell activation. Consequently, this inhibits pathways such as the PI3K/AKT pathway, reducing T cell proliferation and cytokine production, thereby contributing to an immunosuppressive environment^317^ (Fig. 6). This immunosuppressive signaling results in impaired cytokine secretion, decreased proliferation, and diminished cytotoxic functions of T cells, significantly undermining the effectiveness of ICIs in GBM.^318^

##### TGF-β signaling

Transforming growth factor-beta (TGF-β) signaling plays a pivotal role in immune modulation and tumor progression in GBM.^319^ This signaling pathway begins when TGF-β binds to type I and type II serine/threonine kinase receptors, triggering the phosphorylation and activation of receptor-regulated SMAD proteins (SMAD2 and SMAD3). Activated SMAD2/3 complexes with SMAD4 are translocated into the nucleus, where they modulate gene transcription linked to proliferation, differentiation, apoptosis, and immune regulation.^320^ In GBM, TGF-β critically contributes to immune evasion by suppressing the cytotoxic activity of CTLs and NK cells, thereby hindering the host immune response against tumor cells.^321^ Concurrently, TGF-β signaling promotes the proliferation and immunosuppressive functions of Tregs, further dampening immune surveillance.^319^ Additionally, TGF-β drives macrophages toward the M2 phenotype, which is characterized by the secretion of immunosuppressive cytokines such as IL-10 and additional TGF-β, reinforcing the suppressive TME.^322^ TGF-β induces EMT, which increases tumor invasiveness and metastatic potential.^323^ Furthermore, TGF-β signaling positively affects the NF-κB and MAPK pathways, amplifying immunosuppressive and protumorigenic signals in GBM. This interaction enhances tumor progression by promoting immune evasion, T cell suppression, and increased tumor cell survival and invasion^321,323^ (Fig. 6).

##### CD39/CD73-adenosine

The CD39/CD73-adenosine pathway is a critical immunoregulatory mechanism in GBM. CD39 and CD73 are ectonucleotidases that sequentially hydrolyze extracellular ATP to adenosine, an immunosuppressive metabolite. Under physiological conditions, this pathway helps maintain tissue homeostasis by modulating inflammation and preventing excessive immune responses.^324^ In GBM, however, the upregulation of CD39 and CD73 contributes significantly to increased levels of adenosine. This increased adenosine prevents rapid phosphorylation of the ZAP70 kinase as well as AKT and ERK1/2 in T cells.^325^ This leads to the inhibition of effector T cell and NK-cell functions, enhances Treg function and proliferation, reducing the ability of Tregs to mount effective antitumor responses.^326^ Consequently, targeting the CD39/CD73-adenosine pathway has emerged as a promising therapeutic strategy to reverse immunosuppression, enhance antitumor immunity, and potentially improve clinical outcomes in GBM (Fig. 6).

##### IDO1 and kynurenine

IDO1 is a heme-containing enzyme that catalyzes the initial step of tryptophan catabolism through the kynurenine pathway, generating the immunosuppressive metabolite kynurenine. Under physiological conditions, IDO1 modulates immune tolerance by regulating T cell function.^327^ In GBM, IDO1 expression is significantly elevated.^328^ Elevated IDO1 activity in GBM leads to tryptophan depletion and increased kynurenine production, which leads to GCN activation, PD-1/PD-L1 upregulation, AHR activation, and increased kynurenine metabolite production.^329^ This leads to the inhibition of effector T cell and NK cell functions while promoting Treg differentiation, thus impairing antitumor immunity^330^ (Fig. 6). Consequently, targeting the IDO1-kynurenine pathway with specific inhibitors represents a promising therapeutic approach to restore immune function and improve GBM treatment outcomes.^331^

## GBM therapeutics for clinical management

**Fig. 7:**
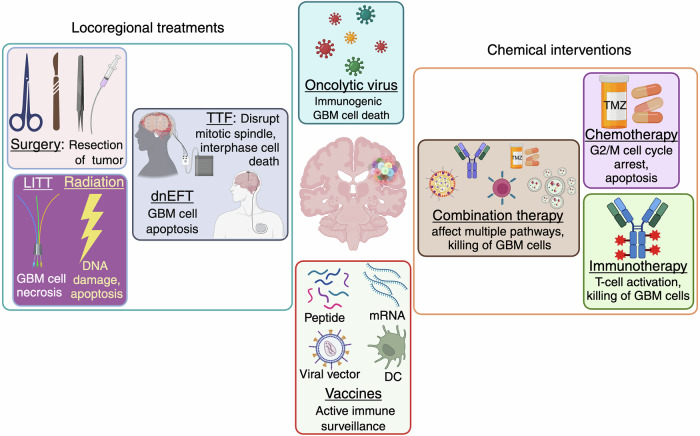
Current and emerging glioblastoma therapeutics for clinical management. The figure provides an overview of current therapeutic strategies used in the clinical management of glioblastoma (GBM). Locoregional treatments include surgical resection, laser interstitial thermal therapy (LITT), and radiation, which aim to eliminate tumor tissue through direct cytotoxic effects. Tumor-treating fields (TTFs) and directional nonrotating electric field therapy (dnEFTs) promote mitotic disruption and apoptosis. Chemical interventions include chemotherapy (TMZ), which induces G2/M arrest and apoptosis, and immunotherapy, which enhances T cell-mediated tumor killing. Oncolytic viruses induce immunogenic cell death, whereas therapeutic vaccines such as peptides, mRNAs, viral vectors, and dendritic cell-based platforms stimulate immune surveillance. Combination therapies leverage multiple modalities to simultaneously target diverse oncogenic pathways, offering a promising route toward overcoming resistance and advancing personalized GBM treatment

Current standard care for GBM patients offers modest survival benefits, but the prognosis remains poor due to tumor recurrence and therapy resistance. Advances in GBM therapeutics have introduced novel approaches, including targeted therapies (e.g., EGFR inhibitors), immunotherapies such as ICIs and CAR-T cell therapies, and noninvasive modalities such as the tumor-treating field (TTF) (Fig. 7). Despite these developments, clinical management remains challenging due to the genetic heterogeneity, invasive nature, and ability of GBM to evade treatment, emphasizing the urgent need for innovative and effective therapeutic strategies.

### Locoregional treatments

#### Surgery

Surgical resection remains a primary treatment for gliomas, contributing to both diagnostic accuracy and therapeutic efficacy. Extensive resection is linked to improved survival in both LGGs and HGGs,^332^ although the precise correlation between the extent of resection and patient outcomes requires further study. In patients with metastatic brain lesions, resection offers survival benefits and enhances quality of life. The evidence suggests that for single brain metastases, surgery is more effective than radiation therapy alone. Fluorescence-guided surgery, particularly with 5-aminolevulinic acid (5-ALA), has significantly improved the extent of resection (EOR) in HGGs.^333^ Clinical trials indicate that 5-ALA enhances gross total resection rates, outperforming conventional surgical methods.^334^ Studies have reported that 5-ALA-guided resection increases EOR, extends OS, and improves PFS. Additionally, compared with intraoperative MRI alone, combining 5-ALA with intraoperative imaging in eloquent brain regions enhances resection success.^335^ These advancements underscore the critical role of fluorescence-guided techniques in neurosurgical oncology, improving tumor visualization, maximizing resection, and ultimately enhancing clinical outcomes.

#### Radiosurgery

The treatment of brain metastases has evolved with a shift toward targeted radiation approaches that enhance tumor control while minimizing neurocognitive decline. Whole-brain radiation therapy, once a standard for patients ineligible for surgical resection or stereotactic radiosurgery (SRS), is now being reevaluated owing to its impact on cognitive function.^336^ SRS techniques, particularly hypofractionated stereotactic radiosurgery, have demonstrated efficacy in treating larger tumors and lesions in eloquent brain regions while potentially engaging immune mechanisms.^337^ Gamma knife (GK) radiosurgery remains a highly precise modality for treating localized tumors, with emerging combinations of GK and bevacizumab showing promise in improving therapeutic outcomes. However, further clinical validation is necessary to refine protocols and mitigate potential treatment biases.^338^ Leading-edge radiosurgery represents an evolving strategy to increase the safety and efficacy of GBM treatment. Gamma tiles or brachytherapy, particularly with cesium-131 isotopes, have shown potential advantages over traditional iodine-125 implants by offering improved tumor control with reduced radiation necrosis,^339^ making it a viable option for recurrent GBM treatment. These advancements reflect a growing emphasis on personalized, precision-driven radiation strategies that aim to optimize both survival and quality of life in patients with brain metastases.

#### Laser-interstitial thermotherapy

Laser-interstitial thermotherapy (LITT) provides a minimally invasive alternative for GBM patients ineligible for surgical resection. By inducing localized hyperthermia, LITT effectively eradicates tumor cells while preserving surrounding tissue, offering advantages over other thermal ablation techniques.^340^ MRI-guided LITT has demonstrated safety and efficacy, with potential benefits such as enhanced BBB permeability for improved drug delivery. While rapid recovery is a key advantage, patient selection and monitoring remain critical to mitigate risks.^341^ LITT shows promise in managing recurrent GBM and hard-to-access HGGs, potentially extending PFS.^342^ Ongoing clinical trials (NCT02880410, NCT03022578, NCT03341806, and NCT03277638) are evaluating its efficacy, safety, and potential synergy with chemotherapy. Similarly, ^18^F-DOPA PET-guided, dose-escalated, hypofractionated proton beam therapy has shown potential survival benefits with manageable adverse effects,^343^ warranting further investigation in phase 2 trials (NCT05781321). These studies will be crucial in defining LITT’s long-term impact and integration into GBM treatment protocols.

#### Focused ultrasound

Focused ultrasound (FU), including high-intensity (HIFU) and low-intensity (LIFU) modalities, has emerged as a potential GBM treatment strategy.^344^ HIFU induces thermal ablation, effectively destroying tumor cells, whereas LIFU, combined with microbubbles, transiently disrupts the BBB, enhancing targeted drug delivery.^345^ This approach significantly increases therapeutic concentrations within tumor tissue while lowering systemic drug exposure.^346,347^ LIFU with MB-mediated delivery improves GBM treatment by increasing drug permeability, downregulating efflux transporters such as P-glycoprotein, and increasing apoptosis^.^^347,348^ Studies have demonstrated that this method enhances the efficacy of chemotherapeutic agents such as etoposide, paclitaxel (PTX), and DOX, reducing tumor growth and extending survival. In preclinical models, PTX liposomes with anti-PD-1 increased survival by 40%, whereas cabazitaxel treatment reduced tumor size by two-thirds.^349,350^ Additionally, nanoparticle-based drug carriers, including mesoporous silica nanoparticles (MSNs) and shRNA-loaded liposomes, further optimize therapeutic delivery and tumor suppression.^351^ The optimization of the acoustic pressure and treatment parameters plays a critical role in maximizing the treatment efficacy. Compared with a lower pressure, a higher acoustic pressure (0.80 MPa) improves tumor inhibition by 64%.^352^ Despite the promising results, challenges remain in standardizing treatment protocols and translating preclinical findings into clinical applications. Future research should focus on refining acoustic parameters, evaluating long-term safety, and integrating focused ultrasound with combination therapies to improve GBM treatment outcomes.

#### Tumor-treating field

The approval of TTF as an adjuvant therapy for newly diagnosed GBM in 2015 introduced a novel approach to treatment. TTF, delivered via the Optune® device, applies low-intensity, intermediate-frequency alternating electric fields to disrupt GBM cell proliferation by interfering with mitotic spindle formation. This noninvasive therapy has shown clinical efficacy in extending OS and PFS, particularly when combined with TMZ.^353^ For recurrent GBM, TTFs can be used as monotherapies, whereas in newly diagnosed patients, TTFs can be combined with the standard chemotherapy TMZ. Ongoing clinical trials (NCT01925573) continue to assess TTFs in combination with bevacizumab and hypofractionated stereotactic irradiation for recurrent GBM. Completed clinical trials have demonstrated that TTFs significantly improve patient outcomes without severe adverse effects, in addition to mild skin irritation. A phase III randomized clinical trial revealed that the combination of TTF with TMZ extended the median survival to 19.2 months in newly diagnosed patients^,^^353,354^ supporting its integration into standard GBM treatment. However, the high cost of TTFs remains a potential barrier to their widespread adoption.

#### Directionally nonrotating electric field therapy

Directionally nonrotating electric field therapy (dnEFT) employs implanted electrodes to deliver continuous, targeted electric fields directly to GBM-affected brain regions. Preclinical studies have demonstrated that dnEFT, which is applied via a clinical-grade spinal cord stimulator or a custom two-electrode system, induces apoptosis in GBM cells and significantly reduces the tumor burden in vivo. dnEFTs exhibited prolonged survival and an immune shift toward an antitumor response in a preclinical model, marked by increased M1 macrophages and reduced M2-associated gene expression.^355^ dnEFTs offer potential advantages over TTFs by maintaining a consistent directional field, potentially enhancing tumor disruption while minimizing resistance development.^355^ Further refinement of electrode placement and field modulation could optimize precision, particularly with real-time adaptation to tumor evolution. Future studies should explore the integration of dnEFTs with immunotherapies, CAR-T cell therapy, and gene editing to increase therapeutic efficacy. Additionally, the development of wearable dnEFT devices may improve patient compliance and accessibility, paving the way for their clinical translation in GBM management.

### Chemical interventions

#### Chemotherapy

TMZ is the standard chemotherapy used for GBM. When radiotherapy is added, the median survival time increases from 12.1 months to 14.6 months. This combination also improved the two-year survival rate from 10.4% to 26.5%, leading to its adoption as the “Stupp protocol”. TMZ, an alkylating agent, exerts cytotoxic and mutagenic effects predominantly by alkylating the O^6^ position of guanine in DNA. The cytotoxic effects of O^6^-methylguanine induce replication arrest, and the accumulation of single-stranded DNA breaks ultimately leads to G2‒M cell cycle arrest and apoptosis. However, its efficacy is significantly limited by DNA repair mechanisms, particularly the activity of MGMT, which reverses guanine methylation and reduces TMZ-induced cytotoxicity.^356^ Patients with MGMT promoter methylation receive the greatest benefit, as their tumors have a diminished capacity to repair TMZ-induced DNA damage. Despite its clinical utility, TMZ resistance remains a major challenge, affecting more than 50% of GBM patients through both intrinsic and acquired mechanisms. In addition to MGMT, multiple molecular pathways contribute to resistance, complicating treatment strategies.^357^ Addressing these mechanisms is crucial for improving GBM therapy, highlighting the need for novel approaches such as combination therapies, MGMT inhibitors, and alternative drug delivery systems to increase TMZ efficacy.

#### Small molecules

Small molecules such as LP-184, LMP400, and Azeliragon are emerging as promising therapeutic candidates in GBM, particularly for overcoming resistance mechanisms. LP-184 has potent anti-GBM activity, including in TMZ-resistant and MGMT-expressing tumors, with effective brain and tumor penetration. Its efficacy is linked to PTGR1 expression, EGFR signaling, and low NER/ERCC3 levels, while spironolactone enhances its cytotoxic effects, suggesting a potential combination therapy for GBM.^358^ Similarly, LMP400 shows high efficacy in PTEN-null GBM, inducing G2/M cell cycle arrest, DNA damage, and apoptosis. Combining the small molecule LMP400 with niraparib enhances cytotoxicity, evades ABC transporters, and extends survival in GBM models, supporting its therapeutic potential.^359^ Azeliragon, currently under evaluation in dose-escalation trials, aims to determine its recommended phase 2 dose while maintaining dose-limiting toxicities below 33% within 28 days and assessing its impact on PSF and OS.^360^ Furthermore, early clinical evaluation of Azeliragon (20 mg/day) with radiotherapy indicates a favorable safety profile, with no dose-limiting toxicities or treatment-related discontinuations.^361^

Additional targeted therapies show promise in GBM management. Dacomitinib effectively penetrates GBM tumors, with 14.3% of patients experiencing clinical benefit and 8.9% achieving PFS for at least one year.^362^ Napabucasin disrupts STAT3 and NF-κB signaling, inhibiting GBM cell proliferation, colony formation, and invasion, while in vivo studies have confirmed its efficacy in impairing GBM growth in xenograft models.^363^ Infigratinib demonstrates superior efficacy over larotrectinib in GBM patients with tyrosine kinase alterations, despite a greater adverse effect profile. Initial bevacizumab therapy has been associated with improved OS, reinforcing its potential role in GBM treatment.^364^ Ogremorphin, a GPR68 inhibitor, induces ferroptosis and cytotoxicity in GBM cells with minimal toxicity, highlighting its potential as a therapeutic strategy. Suppression of ATF4 via GPR68 inhibition further disrupts GBM survival, reinforcing its viability as a target for treatment.^365,366^ Additionally, epigenetic drug treatment of GSCs results in transposable element-derived transcripts that are selectively expressed in cancer cells, generating antigens with potential for targeted immunotherapy. However, the risk of unintended genomic activation raises safety concerns. CRISPR-mediated strategies may help mitigate these risks while optimizing antigen targeting for therapy.^367^ Vorasidenib, a BBB-penetrant IDH1/2 inhibitor, significantly reduces 2-HG levels, reversing gene expression and epigenetic changes in IDH mutant gliomas, highlighting its therapeutic potential.^368^

CSF-1R inhibitors, such as PLX3397, have demonstrated potential in reprogramming TAMs in GBM. TAMs predominantly exhibit an immunosuppressive M2-like phenotype, promoting tumor growth and immune evasion. Inhibiting CSF-1R shifts TAMs toward a proinflammatory M1-like state, enhancing phagocytosis and reducing immune suppression.^314^ Additionally, IDO1 inhibitors, such as epacadostat, target metabolic immunosuppression by blocking the conversion of tryptophan to kynurenine, a pathway that suppresses effector T cell activity while promoting regulatory T cell expansion. By inhibiting IDO1, these therapies restore T cell proliferation and function, enhancing antitumor immunity^328^ (Fig. 6). In combination with TMZ, the PARP inhibitor niraparib enhances immune recognition by upregulating NKG2DL, leading to increased ULBP1/Mult-1 mRNA expression and improved gamma-delta T cell-mediated cytotoxicity in GBM patient-derived xenografts.^369^

These small-molecule inhibitors, either alone or in combination with immune checkpoint blockade, hold promise for overcoming GBM immune resistance. Ongoing clinical trials are evaluating their therapeutic potential, emphasizing the need for synergistic treatment strategies to improve patient outcomes. Continued exploration of combination therapies and immune-targeting agents will be essential for advancing GBM immunotherapy and overcoming resistance mechanisms.

#### Immunotherapy

The highly immunosuppressive TME of GBM significantly limits the efficacy of immune ICIs. Despite PD-L1 expression in tumor cells ranging from 61% to 88%, clinical trials such as CheckMate-143 have failed to show significant survival benefits.^370^ This limited efficacy is linked to poor BBB penetration, low tumor-infiltrating lymphocyte levels, and PTEN mutations, which are prevalent in nonresponders. However, neoadjuvant PD-1 blockade has shown potential in stimulating tumor-specific T cell activation and modulating tumor cell cycle-associated gene expression,^371^ indicating that optimizing treatment timing and combination strategies may improve outcomes. In addition to PD-1/PD-L1 blockade, CTLA-4 inhibition is being explored, with the anti-CTLA-4 monoclonal antibody ipilimumab, which shows promise in the treatment of melanoma brain metastases. Early trials combining ipilimumab, GM-CSF, and bevacizumab in recurrent GBM reported partial responses in 31% of patients.^372^ Ongoing studies are evaluating ipilimumab in combination with TMZ, bevacizumab, and other ICIs.^373^ Additionally, IDO inhibition, which targets metabolic immunosuppression, has shown synergy with anti-PD-L1 and anti-CTLA-4 therapies in preclinical GBM models, resulting in 100% long-term survival.^374^ Furthermore, the current immunotherapeutic drugs in clinical trials are presented in Table 1.

**Table 1:** Clinical trials of immune therapies for GBM treatment

Drug	Target	Clinical trial identifier	Condition	Combination	Phase	Status
Durvalumab	Anti-PD-L1	NCT02336165	GBM	Bevacizumab and radiotherapy	II	Completed
		NCT02794883	Recurrent GBM	Tremelimumb	II	Completed
		NCT02866747	Recurrent GBM	Hypofractionated stereotactic radiotherapy	I/II	Active, not recruiting
Avelumab		NCT03341806	Recurrent GBM	MRI-guided LITT therapy	I	Completed
		NCT03291314	Recurrent GBM	Axitinib	II	Completed
		NCT02968940	IDH-mutant GBM	Hypofractionated stereotactic radiotherapy	II	Completed
		NCT03047473	Newly diagnosed GBM	TMZ	II	Completed
		NCT03750071	Progressive GBM	VXM01	I/II	Active, not recruiting
Atezolizumab		NCT04160494	Recurrent GBM	D2C7-IT	I	Active, not recruiting
		NCT03158389	MGMT unmethylated GBM	Radiotherapy	I/II	Completed
		NCT03174197	Newly diagnosed GBM	Radiotherapy and TMZ	I/II	Active, not recruiting
		NCT03673787	GBM	Ipatasertib	I/II	Recruiting
INCMGA00012	Anti-PD-1	NCT04225039	Recurrent GBM	INCAGN01876 and stereotactic radiosurgery	II	Active, not recruiting
		NCT03532295	Recurrent GBM	Bevacizumab and radiotherapy	II	Active, not recruiting
Nivolumab		NCT02529072	GBM	Dendritic cell vaccine	I	Completed
		NCT02658981	Recurrent GBM	BMS-986016	I	Completed
		NCT02311920	Newly diagnosed GBM	Ipilimumab and TMZ	I	Completed
		NCT03233152	Recurrent GBM	Ipilimumab	I	Active, not recruiting
		NCT04047706	Newly diagnosed GBM	BMS-986205, TMZ and radiotherapy	I	Active, not recruiting
		NCT04003649	Recurrent GBM	IL-13Ralpha2-CAR T-cells and Ipilimumab	I	Recruiting
		NCT04323046	Recurrent GBM	Ipilimumab	I	Recruiting
		NCT03636477	Recurrent or progressive GBM	Ad-RTS-hIL-12 and Veledimex	II	Completed
		NCT03367715	Newly diagnosed, MGMT unmethylated GBM	Ipilimumab and radiotherapy	II	Completed
		NCT02617589	Newly diagnosed, MGMT unmethylated GBM	TMZ	III	Completed
		NCT03452579	Recurrent GBM	Bevacizumab	II	Active, not recruiting
		NCT03743662	Recurrent MGMT methylated GBM	Bevacizumab and radiotherapy	II	Active, not recruiting
		NCT04195139	Newly diagnosed elderly patients with GBM	TMZ	II	Active, not recruiting
		NCT02667587	Newly diagnosed, MGMT methylated GBM	TMZ and radiotherapy	II	Active, not recruiting
		NCT03890952	Recurrent GBM	Bevacizumab	II	Active, not recruiting
		NCT03718767	IDH-mutant GBM with and without hypermutator phenotype	-	II	Recruiting
		NCT04396860	Newly diagnosed, MGMT unmethylated GBM	Ipilimumab	II/III	Active, not recruiting
		NCT04116658	Progressive GBM	Therapeutic vaccine EO2401	I/II	Active, not recruiting
Spartalizumab		NCT03961971	Recurrent GBM	MBG453	I	Active, not recruiting
Pembrolizumab		NCT03726515	Newly diagnosed, MGMT unmethylated GBM	EGFRvIII-CAR T-cells	I	Completed
		NCT03426891	Newly diagnosed GBM	Vorinostat, TMZ and radiotherapy	I	Completed
		NCT02852655	Recurrent or progressive GBM	-	I	Completed
		NCT02313272	Recurrent GBM	Bevacizumab and radiotherapy	I	Completed
		NCT03722342	Recurrent GBM	TTAC-0001	I	Active, not recruiting
		NCT04201873	Recurrent or progressive GBM	Dendritic cell tumor cell lysate vaccine	I	Recruiting
		NCT03277638	Recurrent GBM	Laser interstitial thermotherapy	I	Recruiting
		NCT02287428	MGMT unmethylated, newly diagnosed GBM	Radiotherapy, TMZ and neoantigen vaccine	I	Recruiting
		NCT02798406	Recurrent GBM	Adenovirus (DNX-2401)	II	Completed
		NCT03018288	Newly diagnosed GBM	HSPPC-96 and TMZ	II	Completed
		NCT04013672	Recurrent GBM	SurVaxM, sargramostim, montanide, and ISA51	II	Active, not recruiting
		NCT03661723	Recurrent GBM	Bevacizumab and radiotherapy	II	Active, not recruiting
		NCT03899857	Newly diagnosed GBM	-	II	Active, not recruiting
		NCT03405792	Newly diagnosed GBM	Tumor treating field (TTF)	II	Active, not recruiting
		NCT02337686	Recurrent GBM	-	II	Active, not recruiting
		NCT03797326	GBM	Lenvatinib	II	Active, not recruiting
		NCT03347617	Newly diagnosed GBM	Ferumoxytol	II	Active, not recruiting
		NCT04479241	Recurrent GBM	Oncolytic polio/rhinovirus recombinant (PVSRIPO)	II	Active, not recruiting
		NCT01174121	Progressive GBM	Cyclophosphamide, fludarabine, aldesleukin and TIL	II	Recruiting
		NCT03412877	GBM	Cyclophosphamide, fludarabine, aldesleukin and TCR	II	Recruiting
		NCT02311582	Recurrent GBM	MRI-guided laser ablation	I/II	Completed
		NCT03665545	Recurrent GBM	IMA950/poly-ICLC	I/II	Active, not recruiting
		NCT02658279	Recurrent GBM with a hypermutator phenotype	-	-	Active, not recruiting
Cemiplimab		NCT04006119	Recurrent or progressive GBM	Ad-RTS-hIL-12 and Veledimex	II	Completed
		NCT03491683	Newly diagnosed GBM	INO-5401 and INO-9012	I/II	Active, not recruiting
BMS-986205 (Linrodostat)	Anti-IDO1	NCT04047706	Newly diagnosed GBM	Nivolumab, TMZ and radiotherapy	I	Active, not recruiting
Indoximod		NCT02502708	Newly diagnosed GBM	TMZ, radiotherapy, cyclophosphamide and etoposide	I	Completed
		NCT04049669	Progressive GBM	TMZ, radiotherapy, cyclophosphamide, etoposide and Lomustine	II	Recruiting
		NCT02052648	GBM	Bevacizumab, TMZ and stereotactic radiation	I/II	Completed
Tremelimumab	Anti-CLTA4	NCT02794883	Recurrent GBM	Durvalumab	II	Completed
Ipilimumab		NCT02311920	newly diagnosed GBM	Nivolumab and TMZ	I	Completed
		NCT03233152	Recurrent GBM	Nivolumab	I	Active, not recruiting
		NCT04003649	Recurrent GBM	IL13Ralpha2-CAR T-cells and Nivolumab	I	Recruiting
		NCT04323046	Recurrent GBM	Nivolumab	I	Recruiting
		NCT03367715	newly diagnosed, MGMT unmethylated GBM	Nivolumab and radiotherapy	II	Completed
		NCT02052648	GBM	Bevacizumab, TMZ and stereotactic radiation	I/II	Completed
		NCT04396860	Newly diagnosed, MGMT unmethylated GBM	Nivolumab	II/III	Active, not recruiting
APX005M	Phage based therapy	NCT03389802	GBM	-	I	Active, not recruiting
NK-92/5.28.z	NK cell therapy	NCT03383978	Recurrent HER2-positive GBM	-	I	Active, not recruiting
	Adoptive cell therapy					
EGFRvIII CAR-T		NCT03726515	Newly diagnosed, MGMT unmethylated GBM	Pembrolizumab	I	Completed
IL-13Ralpha2- autologous T-lymphocytes		NCT02208362	Recurrent GBM	-	I	Active, not recruiting
B7-H3 CAR-T		NCT04385173	Recurrent GBM	TMZ	I	Recruiting
Chlorotoxin (EQ)-CD28- CD3zeta-CD19T		NCT04214392	Recurrent GBM	-	I	Recruiting
IL-13Ralpha2, CAR-T		NCT04003649	Recurrent GBM	Nivolumab and Ipilimumab	I	Recruiting
TIL		NCT01174121	Progressive GBM	Cyclophosphamide, fludarabine, aldesleukin and Pembrolizumab	II	Recruiting
Autologous T-cells, neoantigens		NCT03412877	GBM	Cyclophosphamide, fludarabine, aldesleukin and Pembrolizumab	II	Recruiting

Strategies targeting TAMs focus on blocking recruitment via CCL2-CCR2 inhibition, promoting M1 polarization via CD47-SIRPα blockade, or depleting M1 polarization via CSF-1R inhibitors.^375,376^ These approaches have demonstrated preclinical efficacy but require further validation in GBM patients. NK cell-based therapies, including CYNK-001, are in early clinical trials, with CAR-NK cells engineered to target GBM-specific antigens such as EGFRvIII, HER2, IL-13Rα2, and CD133 showing preclinical efficacy.^377,378^ CAR-T cell therapy also presents potential, with intrathecal bivalent CAR-T cells targeting EGFR and IL-13Rα2 demonstrating early tumor reduction.^379^ Additionally, PTP4A2 regulates GBM recurrence via roundabout guidance receptor 1 (ROBO1), and CAR-T cell targeting of ROBO1 improves survival in recurrent GBM models, highlighting a potential therapeutic strategy for GBM.^380^

Overall, the failure of single-agent ICIs underscores the necessity of combination strategies addressing immune evasion and TME constraints. Continued research and clinical trials are essential for refining immunotherapy approaches and overcoming resistance in GBM treatment.

#### Targeted therapy

Targeted therapy, which is designed to selectively inhibit molecular pathways critical for tumor progression while minimizing systemic toxicity, has become a cornerstone of GBM treatment. Unlike conventional therapies that broadly affect both malignant and normal cells, targeted approaches aim to improve efficacy while mitigating adverse effects. Bevacizumab, a VEGF inhibitor, is approved for recurrent GBM and effectively delays disease progression; however, its impact on OS remains limited.^381^ Similarly, RTK inhibitors targeting PDGFR (e.g., olaratumab and crenolanib) and c-KIT (avapritinib) show promising BBB penetration but are hindered by resistance mechanisms.^382^ EGFR inhibitors such as gefitinib, cetuximab, and ABT-414 have demonstrated variable efficacy, primarily due to tumor heterogeneity and adaptive resistance. In addition to growth factor receptors, the c-MET/HGF pathway has emerged as a key driver of GBM invasion and therapy resistance. Dacomitinib, an EGFR inhibitor, reduces tumor viability and self-renewal in EGFR-amplified GBM, although its effectiveness is influenced by the PTEN status,^383,384^ Despite their initial promise, c-MET inhibitors such as onartuzumab and rilotumumab have failed to significantly improve survival outcomes. Onartuzumab tended to reduce tumor growth but lacked clinical efficacy when combined with bevacizumab. Similarly, rilotumumab did not show notable antitumor activity as a monotherapy or in combination with bevacizumab. These findings highlight the need for refined therapeutic targeting strategies in c-MET-driven GBMs.

The PI3K/AKT/mTOR pathway, which is frequently dysregulated in GBM, remains a key therapeutic target. However, inhibitors such as buparlisib have not demonstrated significant clinical benefits, reinforcing the need for dual mTORC1/2 inhibitors such as vistusertib.^385,386^ Epigenetic modulators, including DNMT and BET inhibitors, are being explored as potential alternatives. PARP inhibitors have gained traction in GBM therapy, particularly in combination with agents that disrupt DNA repair mechanisms. Stellettin B sensitizes GBM cells to PARP inhibitors (e.g., rucaparib and olaparib) by downregulating BRCA1/2 and RAD51, leading to synthetic lethality and tumor apoptosis.^387^ BET inhibitors, such as Birabresib, further enhance this effect by impairing DNA repair and disrupting cell cycle progression. Notably, compared with monotherapies, sequential PARP-BET inhibitor treatment maintains sustained antitumor activity while minimizing toxicity.^388^ These findings underscore the potential of targeting chromatin regulators alongside DNA damage response pathways.

Resistance to targeted therapies remains a major barrier in GBM treatment. Although promising for disrupting tumor proliferation and immune evasion, STAT3 and JAK inhibitors face significant limitations due to poor BBB penetration.^279^ Overcoming these obstacles requires innovative drug delivery approaches, such as nanoparticle-based carriers and convection-enhanced delivery, to enhance the therapeutic reach. The complex and adaptive nature of GBM necessitates combination strategies that disrupt compensatory pathways while improving drug retention in tumor cells. The integration of targeted agents with immunotherapy or radiation has potential for overcoming resistance. As research progresses, precision medicine approaches and biomarker-driven strategies will be critical in refining targeted therapy regimens.

While targeted therapies have made significant strides in GBM management, their clinical efficacy remains inconsistent due to tumor heterogeneity and acquired resistance. Refining therapeutic combinations, improving drug delivery mechanisms, and leveraging biomarker-based treatment selection are critical for advancing GBM treatment.

#### Targeted combination therapies

GBM treatment resistance often arises from extensive intra- and intertumoral heterogeneity, necessitating combination therapies targeting multiple pathways. Table 2 outlines clinical trials evaluating combination strategies alongside radiotherapy and/or chemotherapy. The combination of radiotherapy with PCV has shown promising long-term outcomes. A 140-month follow-up study demonstrated significantly prolonged OS and PFS compared with adjuvant radiotherapy alone.^389^ Bevacizumab has also been evaluated in combination with TTFs and as an early intervention at the first recurrence. However, studies comparing bevacizumab-radiation combinations with bevacizumab monotherapy have yielded mixed results. Some retrospective analyses have suggested improved OS in patients with recurrent GBM,^338,390^ whereas others have reported that the addition of resurgery significantly enhances survival compared with bevacizumab alone. However, conflicting data from other studies indicate no significant survival advantage.^391^ The variability in the GBM response to combination therapies underscores the importance of personalized treatment strategies. Ongoing clinical trials continue to explore the integration of targeted agents with immunotherapies and standard treatments to improve patient survival. Advancements in biomarker-driven therapy selection, adaptive resistance monitoring, and novel drug delivery technologies are essential for more effective and personalized treatment regimens for GBM patients.

**Table 2:** Clinical trials of targeted and combination therapies for GBM treatment

Drug	Target	Condition	Clinical trial identifier	Combination	Phase	Status
Erlotinib	EGFR	Relapsed/refractory GBM	NCT00301418	-	I/II	Completed
		Newly diagnosed GBM	NCT00720356	Bevacizumab and TMZ	II	Completed
		Progressive or recurrent GBM	NCT00445588	Sorafenib	II	Completed
Cetuximab		Relapsed/refractory GBM	NCT02800486	Mannitol and radiotherapy	II	Recruiting
		Newly diagnosed GBM	NCT02861898	Mannitol	I/II	Recruiting
Osimertinib		Recurrent GBM	NCT03732352	Fludeoxyglucose F-18 PET	II	Active, not recruiting
Nimotuzumab		Newly diagnosed GBM	NCT03388372	Radiotherapy and TMZ	II	Completed
		Newly diagnosed GBM	NCT00753246	-	III	Completed
Rindopepimut		Newly diagnosed, surgically resected, EGFRvIII-positive GBM	NCT01480479	TMZ	III	Completed
		Newly diagnosed GBM	NCT00458601	Radiotherapy and TMZ	II	Completed
Depatuxizumab		Recurrent GBM	NCT02343406	TMZ	II	Completed
		GBM	NCT01800695	Radiotherapy and TMZ	I	Completed
		Newly diagnosed GBM With EGFR amplification	NCT02573324	Radiotherapy and TMZ	III	Completed
		Newly diagnosed or recurrent GBM	NCT02590263	Radiotherapy and TMZ	I/II	Completed
AZD4547	FGFR	GBM with FGFR-TACC gene fusion	NCT02824133	-	I/II	Completed
Cediranib	VEGFR	Recurrent GBM	NCT02974621	Bevacizumab and Olaparib	II	Active, not recruiting
Pazopanib		Recurrent GBM	NCT01931098	Topotecan	II	Completed
		Newly diagnosed GBM	NCT02331498	-	I/II	Recruiting
Vandetanib		Recurrent GBM	NCT00821080	Sirolimus	I	Completed
Sorafenib		Recurrent GBM	NCT01434602	Everolimus	I/II	Completed
Lenvatinib		GBM	NCT03797326	Pembrolizumab	II	Active, not recruiting
Temsirolimus	PI3K/AKT/mTOR	Recurrent GBM	NCT00329719	Sorafenib and Tosylate	I/II	Completed
		Recurrent GBM	NCT00335764	Sorafenib and Tosylate	I/II	Completed
		Recurrent GBM	NCT0223849	Perifosine	I	Active, not recruiting
		Recurrent GBM	NCT02343406	TMZ	II	Completed
Everolimus		Recurrent GBM	NCT03834740	Ribociclib	Early I	Completed
		Newly diagnosed GBM	NCT00553150	Radiotherapy and TMZ	I/II	Completed
		Newly diagnosed GBM	NCT01062399	Radiotherapy and TMZ	I/II	Completed
		Recurrent GBM	NCT01434602	Sorafenib	I/II	Completed
Buparlisib		Relapsed/refractory GBM	NCT01349660	Bevacizumab	I/II	Completed
		Newly diagnosed GBM	NCT01473901	Radiotherapy and TMZ	I	Completed
		Recurrent GBM	NCT01934361	Lomustine or Carboplatin	I	Completed
		Recurrent GBM	NCT01339052	-	II	Completed
BBI608	STAT-3	Recurrent or progressed GBM	NCT02315534	TMZ	I/II	Completed
Palbociclib	CDK	Newly diagnosed GBM without MGMT promoter methylation	NCT03158389	Radiotherapy	I/II	Completed
Ribociclib		Recurrent GBM	NCT03834740	Everolimus	Early I	Completed
		Preoperative GBM	NCT02933736	-	Early I	Active, not recruiting
Abemaciclib		Recurrent GBM	NCT04074785	Bevacizumab	Early I	Active, not recruiting
		Recurrent GBM	NCT02981940	-	II	Active, not recruiting
		Recurrent GBM	NCT04391595	LY3214996	Early I	Recruiting
		GBM	NCT02977780	TMZ	II	Recruiting
Olaparib	PARP	Recurrent GBM	NCT02974621	Bevacizumab and Cediranib	II	Active, not recruiting
		Newly diagnosed and recurrent GBM	NCT04614909	Radiotherapy, Pamiparib and TMZ	I	Recruiting
		GBM	NCT03212274	-	II	Active, not recruiting
Pamiparib		Newly diagnosed and recurrent GBM	NCT03150862	Radiotherapy and TMZ	I/II	Completed
		Recurrent GBM	NCT03914742	TMZ	I/II	Completed
		Recurrent GBM	NCT03749187	TMZ	I	Recruiting
Niraparib		Newly diagnosed and recurrent GBM	NCT05076513	Fractionated radiotherapy	0/II	Active, not recruiting
Rilotumumab	MET	Recurrent GBM	NCT01113398	Bevacizumab	II	Completed
Onartuzumab		Recurrent GBM	NCT01632228	Bevacizumab	II	Completed
Capmatinib		GBM	NCT02386826	Bevacizumab	I	Completed
Vorinostat	HDAC	Newly diagnosed GBM	NCT03426891	Radiotherapy, Pembrolizumab and TMZ	I	Completed
		Recurrent GBM	NCT00555399	Isotretinoin and TMZ	I/II	Active, not recruiting
		Newly diagnosed GBM	NCT00731731	Radiotherapy and TMZ	I/II	Completed
		Recurrent GBM	NCT01738646	Bevacizumab	II	Completed
Fimepinostat		Recurrent GBM	NCT03893487	Surgery	Early I	Active, not recruiting
AMG232	MDM2	Newly diagnosed and recurrent GBM	NCT03107780	Radiotherapy	I	Recruiting
RG7388		Newly diagnosed GBM without MGMT promoter methylation	NCT03158389	Radiotherapy	I/II	Completed
BCA101	TGF-β	GBM	NCT04429542	Pembrolizumab	II	Recruiting
Galunisertib		Recurrent GBM	NCT01582269	Lomustine	I/II	Active, not recruiting
AZD1390	ATM	Newly diagnosed and recurrent GBM	NCT03423628	Radiotherapy	I	Recruiting
Veliparib		GBM	NCT01514201	Radiotherapy and TMZ	I/II	Completed
		GBM	NCT03581292	Radiotherapy and TMZ	II	Active, not recruiting
		Newly diagnosed GBM with MGMT promoter hypermethylation	NCT02152982	TMZ	II/III	Active, not recruiting
Bortezomib	Proteasome	Recurrent GBM with unmethylated MGMT promoter	NCT03643549	TMZ	I/II	Recruiting
		Recurrent GBM	NCT01435395	Bevacizumab and TMZ	I	Completed
Ixazomib		GBM	NCT02630030	-	Early I	Completed
Marizomib		Newly diagnosed GBM	NCT03345095	Radiotherapy and TMZ	III	Completed
		GBM	NCT02330562	Bevacizumab	I/II	Completed
		Newly diagnosed GBM	NCT02903069	Radiotherapy, TMZ and TTF	I	Completed
		Newly diagnosed GBM	NCT03463265	Nab-rapamycin	II	Completed
Azeliragon	RAGE	Newly diagnosed GBM	NCT05635734	Radiotherapy and TMZ	Ib/II	Active, not recruiting
			NCT05986851	Radiotherapy	II	Active, not recruiting
Imipramine	Serotonin, Norepinephrine	Recurrent GBM	NCT04863950	-	II	Recruiting
Anlotinib	TKI	Recurrent GBM	NCT04004975	-	I/II	Unknown
Ponatinib	c-KIT	Bevacizumab-refractory GBM	NCT02478164	-	II	Completed
Erdafinitib	FGFR fusion	IDH-wild type GBM	NCT05859334	-	II	Recruiting
BGJ398		Recurrent GBM	NCT01975701	-	II	Completed
Entrectinib	NTRK fusion	Primary brain tumors	NCT02568267	-	II	Active, not recruiting
		Advanced or metastatic solid or primary brain tumors	NCT02650401	-	I/II	Active, not recruiting
Larotrectinib		NTRK-fusion positive solid tumors	NCT02576431	-	II	Active, not recruiting
PLB1001	PTPRZ1-MET fusion	Recurrent high-grade gliomas	NCT02978261	-	I	Completed
Vorasidenib	IDH	Residual or recurrent grade II glioma	NCT04164901	-	III	Active, not recruiting

Combining ICIs with complementary therapies is a promising approach for improving GBM treatment efficacy. When integrated with ICIs, radiotherapy enhances immunogenic cell death, leading to the release of tumor-associated antigens that activate DCs and prime T cells. This process improves T cell infiltration and activity, potentially overcoming immune resistance mechanisms inherent to GBM.^392,393^ Another emerging strategy involves personalized cancer vaccines that target neoantigens unique to GBM and are designed to elicit robust tumor-specific immune responses. These vaccines, when combined with ICIs, significantly increase T cell activation and amplify antitumor immunity, leading to increased tumor rejection rates and potentially improved clinical outcomes.^394^

The integration of personalized vaccines with ICIs represents a highly promising approach that is currently undergoing extensive research and clinical validation. These targeted combination strategies hold significant potential in overcoming GBM immune barriers, emphasizing the need for continued investigation and clinical development to refine their effectiveness in GBM immunotherapy.

#### Vaccines

Vaccine-based immunotherapy has emerged as a promising strategy for GBM treatment, with the aim of enhancing tumor-specific immune responses. By leveraging tumor antigens, these vaccines activate adaptive immunity and promote sustained immune surveillance against GBM cells. Currently, four primary vaccine-based strategies are under investigation for GBM: peptide vaccines, DNA vaccines, cell-based vaccines, and mRNA vaccines.^395^ Peptide and DNA vaccines introduce tumor-specific antigens or DNA sequences encoding tumor-associated proteins to elicit an adaptive immune response.^396^ Peptide vaccines target well-defined tumor antigens, whereas DNA vaccines utilize plasmid DNA to drive antigen expression in host cells. Cell vaccines, particularly DC vaccines, involve priming DCs derived from peripheral blood mononuclear cells with tumor antigens.^397^ rWTC-MBTA is an autologous vaccine that induces complete tumor regression in GBM models through T cell activation, long-term immune memory, and minimal toxicity. Its ability to enhance DC activation and T cell cytotoxicity suggests its potential for combination with other immunotherapies to improve GBM treatment.^398^ On the other hand, mRNA vaccines utilize viral vectors loaded with mRNAs encoding tumor antigens to induce robust immune responses.^399^ This strategy has gained attention because of its ability to induce strong immune responses and its adaptability in targeting multiple GBM-associated antigens.

Despite encouraging preclinical and early-phase clinical trial results, vaccine efficacy in GBM remains inconsistent. Key challenges include antigenic variability among tumors, limited infiltration of immune cells into the CNS, and the presence of immunosuppressive factors such as Tregs and MDSCs.^395^ Combination strategies integrating vaccines with ICIs, cytokine adjuvants, or personalized neoantigen approaches are being explored to enhance vaccine-induced immune responses. Another exciting avenue involves the development of personalized cancer vaccines targeting neoantigens unique to GBM tumor cells. These tailored vaccines aim to induce strong, tumor-specific immune responses, particularly those that enhance T cell activation. When utilized alongside ICIs, personalized cancer vaccines can significantly amplify immune responses, increasing tumor rejection rates and potentially leading to superior clinical outcomes. The combined strategy of personalized vaccines and ICIs represents a highly promising approach that is currently undergoing extensive research and clinical validation.^394,400^ Ongoing clinical trials (Table S8) continue to assess the therapeutic potential of GBM vaccines, with an emphasis on optimizing antigen selection, delivery methods, and immune modulation strategies. Further research is essential to refine vaccine-based immunotherapy and integrate it into multimodal GBM treatment paradigms.

#### Precision and personalized therapy

Advancements in drug screening and precision medicine are shaping the future of GBM treatment. A novel 3D brain cancer chip constructed from a photopolymerizable poly(ethylene) glycol diacrylate (PEGDA) hydrogel represents a significant breakthrough in drug testing. This platform mimics the TME by enabling controlled chemical release and replicating cell-to-cell and cell-to-matrix interactions. Its application in evaluating the combined effects of pitavastatin and irinotecan underscores its potential for high-throughput drug screening and personalized therapy, requiring minimal tumor biopsy samples.^401^ Gene expression profiling and mutation analysis further enhance the ability to develop targeted and personalized therapies. Despite the challenges posed by the spatial and temporal heterogeneity of tumors, this approach allows for the identification of effective therapeutic responses on the basis of genetic similarity. Multifocal tumors with PIK3CA mutations exhibit variable drug responses,^402^ emphasizing the necessity for comprehensive genomic analysis across multiple tumor regions to refine treatment strategies.

Additionally, induced neural stem cells (iNSCs) derived from patient skin cells present a promising avenue for personalized cell therapy. Engineered iNSCs can selectively induce apoptosis in GBM cells while retaining their differentiation potential. In preclinical models, iNSCs successfully target distant tumor sites and deliver therapeutic molecules such as TRAIL, improving survival rates by overcoming the BBB and minimizing systemic toxicity.^403^ While this approach holds potential, further validation is needed to establish its safety and efficacy for clinical application. Together, these advancements in drug screening technology, genomic profiling, and cell-based therapy highlight the shift toward more precise and effective GBM treatment strategies. Integrating these approaches could lead to improved therapeutic outcomes and personalized treatment regimens tailored to individual tumor characteristics.

## Exploration of new horizons in GBM therapy

### Stem cell therapy

The emergence of stem cell-based therapy represents a transformative approach in GBM treatment, offering a promising solution to major therapeutic challenges such as BBB penetration, tumor heterogeneity, and immune evasion.^404^ Neural stem cells (NSCs) and mesenchymal stem cells (MSCs) have garnered attention for their intrinsic tumor-homing ability, allowing them to serve as efficient vehicles for targeted drug delivery and immunomodulation in GBM. Their ability to migrate toward tumor sites is mediated by chemokine receptors such as CXCR1, CXCR2, CXCR4, and CCR2, which respond to glioma-secreted signals such as IL-8, stromal cell-derived factor 1, and monocyte chemoattractant protein-1 (MCP-1).^405,406^ This glioma-tropic migration enables direct therapeutic intervention within the TME, significantly improving drug bioavailability and reducing systemic toxicity. In addition to their innate migratory properties, genetically engineered NSCs and MSCs provide a versatile platform for delivering cytotoxic agents, cytokines, and OVs to GBMs. Patient-derived human-induced NSCs (hi-NSCs) offer a personalized therapeutic strategy, further enhancing compatibility and reducing the risk of immune rejection. In preclinical studies, TRAIL-expressing hi-NSCs have been shown to selectively induce apoptosis in GBM cells, leading to improved survival outcomes.^407^ Additionally, NSCs have been modified to secrete immunostimulatory cytokines such as IL-7, IL-12, and IL-23, promoting immune cell recruitment and antitumor activity.^408^

Stem cells also serve as delivery vehicles in enzyme/prodrug-based therapy and OV therapy. The FDA-approved HB1.F3. The CD NSC line, which converts 5-fluorocytosine into the active chemotherapeutic agent 5-fluorouracil (5-FU), has shown promising tumor localization and safety profiles in early-phase clinical trials.^409^ Similarly, carboxylesterase-releasing NSCs are being tested in combination with irinotecan to enhance its active metabolite, SN-38, for improved efficacy against HGGs. In virotherapy, NSC-mediated delivery of glioma-restricted adenoviruses (CRAd-S-pk7) enhances viral distribution while reducing immune clearance, demonstrating significant survival benefits in clinical trials.^410,411^ Overall, stem cell therapy represents a paradigm shift in GBM treatment, leveraging the ability of NSCs and MSCs to overcome therapeutic barriers, enhance precision drug delivery, and modulate the tumor immune microenvironment. While ongoing clinical trials continue to assess their safety and efficacy, stem cell-based strategies have the potential to redefine GBM management, paving the way for personalized and more effective treatment modalities for this aggressive form of brain cancer.

### Oncolytic viruses

Oncolytic viruses (OVs) represent a promising therapeutic strategy for GBM, leveraging their ability to selectively infect and lyse rapidly proliferating tumor cells while transforming the immunosuppressive TME into an immune-responsive state. Unlike other malignancies, GBM lacks distant metastases, making it an ideal candidate for OV therapy, as the virus remains localized, maximizing its tumor-specific effects. In addition to direct oncolysis, OVs trigger immunogenic cell death, releasing tumor-associated antigens, damage-associated molecular patterns, and viral pathogen-associated molecular patterns, which enhance antigen presentation and stimulate immune activation.^412^ This process reverses the “cold” tumor phenotype of GBM by promoting APC recruitment, activating CD8^+^ CTLs, and counteracting the immunosuppressive influence of TAMs and Tregs. The highly immunosuppressive TME of GBM, characterized by M2-polarized TAMs and T cell exhaustion, limits the effectiveness of conventional immunotherapies.^413^ OVs counteract these suppressive mechanisms by inducing an inflammatory response and increasing immune infiltration, facilitating sustained antitumor immune attack. OVs fall into two major categories: replication-competent viruses, which selectively replicate within tumor cells, and replication-deficient viral vectors, which deliver therapeutic genes. Engineered viruses, such as adenoviruses (Ads), herpes simplex viruses (HSVs), vaccinia viruses (VVs), vesicular stomatitis viruses (VSVs), polioviruses, and measles viruses (MVs), have been optimized for tumor selectivity, enhanced oncolysis, and immune modulation. The oncolytic HSV-G47∆ agent demonstrated promising therapeutic potential in GBM, achieving a 1-year survival rate of 84.2% and a median OS of 20.2 months posttreatment, with a favorable safety profile. Its ability to induce TIL recruitment and repeated lesion responses on imaging contributed to its approval as Japan’s first OV therapy for GBM.^414^ More than 20 different OVs, including HSV-1, Ad, reovirus, NDV, MV, and poliovirus, have progressed to clinical trials for GBM,^415–419^ underscoring their therapeutic potential.

Effective OV delivery remains a critical challenge, with intratumoral administration preferred to avoid immune clearance.^420^ Convection-enhanced delivery, which uses a pressure gradient to bypass the BBB, has shown success in delivering recombinant nonpathogenic polio-rhinovirus chimeras into the CNS. Furthermore, innovative biological vectors such as NSCs and lymphocytes are being explored for OV delivery, improving viral biodistribution and persistence. Phase I clinical trials using NSC-mediated OV delivery (NSC-CRAd-S-pk7) in GBM patients have demonstrated enhanced safety and efficacy with minimal toxicity.^410^ Similarly, the use of lymphocytes modified with the herpesvirus saimiri represents a novel approach for OV transport.^421^ Table S9 presents the OVs used in clinical trials for GBM treatment. Advancing OV therapy for GBM requires continued optimization of viral engineering, immune modulation, and delivery strategies. The integration of OVs with ICIs, CAR-T cell therapy, and radiation is under investigation to further enhance therapeutic efficacy. With ongoing clinical trials and novel bioengineering approaches, OV-based therapies hold great potential for transforming GBM treatment, offering a multifaceted approach that combines direct tumor lysis with potent immune activation.

#### Combination of OVs with chemo-, radio- and immunotherapy

The combination of OVs with ICIs has shown promising results in the treatment of GBM. Studies have shown that MV infection upregulates PD-L1 expression in GBM models, increasing the susceptibility of tumors to anti-PD-1 therapy and significantly improving survival compared with monotherapy.^422,423^ Similarly, engineered reovirus expressing GM-CSF demonstrated enhanced survival with anti-PD-1 therapy.^424^ Another OV, DNX-2401, combined with anti-PD-1 therapy has led to a substantial shift in the TME and prolonged survival in preclinical GBM models.^425^ Strong synergy is also observed with IL-12-expressing oHSVs combined with anti-PD-1 and anti-CTLA-4 therapies, which effectively eliminate GSCs and boost immune activity.^426,427^ Similarly, the efficacy of the combination of VSV engineered to express tumor-specific antigens such as HIF-2α, Sox-10, and c-Myc with dual checkpoint blockade was improved.^428^ Clinical trials using DNX-2401 with pembrolizumab (anti-PD-1) reported a 100% nine-month survival rate in GBM patients.^429^

Genetically modified OVs expressing cytokines or fusion proteins have also demonstrated improved outcomes. When combined with agents such as rapamycin and GBM-specific neoantigens, vaccinia virus or Myxoma virus expressing the IL-15Rα-IL-15 fusion enhances survival.^430^ VSV encoding IFN-β has been explored alongside CAR-T cell therapy targeting EGFRvIII, highlighting the need for further optimization to fully understand immunological interactions.^431^ Combining OVΔ-24-RGD OVs with TMZ increased CD8^+^ T cell infiltration and prolonged survival.^432^ Other OVs, such as Toca 511 and TG6002, serve as prodrug-converting agents, transforming 5-FC into cytotoxic 5-FU^433^ and offering alternative therapeutic options. In addition to direct oncolysis and immune activation, engineered OVs are being leveraged to enhance adoptive cell therapies. HER2-CAR virus-specific T cells (HER2-CAR-VSTs) have shown safety and clinical efficacy in GBM patients.^434^ Bispecific T cell engagers (BiTEs) represent another innovative strategy, linking T cells to tumor antigens, preventing antigen escape, and amplifying antitumor responses.^435^ The continuous development of OVs as combinatorial immunotherapies, particularly with ICIs, CAR-T cells, and BiTEs, holds great promise for overcoming GBM’s immunosuppressive barriers and improving patient survival.

### Extracellular vesicles for GBM treatment

#### EVs as therapeutic targets

EVs play a crucial role in GBM, facilitating tumor progression by increasing proliferation, invasiveness, chemoresistance, and immune evasion. Disrupting EV release, uptake, and circulation represents a promising therapeutic strategy for mitigating GBM progression. Several approaches have been identified, including targeting EVs in transit through hemodialysis, inhibiting their release via agents such as berberine and ketoconazole, or repurposing existing drugs such as heparin and reserpine.^436^ Berberine not only enhances photodynamic therapy sensitization but also inhibits GBM proliferation by suppressing fatty acid synthesis and reducing EV secretion.^437^ Additionally, heparan sulfate proteoglycans (HSPGs) modulate EV uptake in GBM cells, and strategies targeting HSPGs have been demonstrated to reduce EV internalization.^438^ However, the lack of cancer cell specificity in heparin-mediated EV inhibition poses a challenge for clinical application. Notably, GDEVs can activate glycolysis in human bone marrow mesenchymal stem cells (hBMSCs), leading to tumor-supportive transformation. This interaction between exosomes and hBMSCs highlights the potential of targeting EV-mediated signaling in GBM therapy.^439^

Gene and RNA therapies have gained traction in GBM treatment, with emerging research identifying multiple lncRNAs, miRNAs, and circRNAs within GDEVs that contribute to tumor progression. Key lncRNAs such as POUF3F3 and TALC significantly reshape the GBM microenvironment. POUF3F3 drives angiogenesis and tumor expansion, whereas TALC induces M2-macrophage polarization and upregulates the complement components C5/C5a, fostering chemoresistance.^440^ Other oncogenic lncRNAs, including MALAT1, MEG3, NEAT1, and HOTAIR, promote EMT and contribute to the aggressive phenotype of GBM.^441^ Moreover, targeting the mTOR pathway to suppress GDEV production offers a potential strategy to disrupt the supportive TME and curb tumor progression.^442^ Understanding the specific cargo within GDEVs is vital for designing targeted therapies. Proteomic analysis of GDEVs revealed that EGFRvIII, PDGFR, and HER2 are linked to enhanced tumor cell proliferation. In addition, proteins such as L1CAM, ANXA1, ITB1, and ACTR3 have been associated with increased tumor invasiveness. Furthermore, MRP1 has been shown to contribute to chemoresistance. Additionally, GDEVs are enriched with proangiogenic factors such as VEGF, TGF-β1, and CXCR4, which facilitate endothelial proliferation and vascular remodeling, as well as immunosuppressive mediators such as PD-L1 and MDSCs, which contribute to immune evasion.^443^ By selectively targeting these GDEV-associated proteins and pathways, novel therapeutic strategies can be developed to inhibit tumor growth, modulate the TME, and improve GBM treatment efficacy. Research into the molecular composition and functional impact of GDEVs is essential for refining these therapeutic strategies, with the potential to develop more precise and personalized treatments for GBM patients.

#### EVs as therapeutic candidates

EVs have emerged as promising therapeutic candidates for GBM because of their ability to influence key biological processes, including cell proliferation, apoptosis, differentiation, and immune modulation. Unlike viral vectors, EVs exhibit minimal adverse gene expression effects, enhancing their therapeutic potential for GBM treatment.^444^ Exosomes derived from MSCs engineered to carry tumor-suppressive miRNAs offer a targeted strategy to modulate GBM progression.^445^ Studies have demonstrated that MSC-derived exosomes loaded with miR-146b effectively reduce GBM cell proliferation and invasion in vitro while significantly decreasing tumor volume and improving survival in vivo.^446^ Similarly, the delivery of miR-124 and miR-145 via exosomes has been shown to suppress tumor growth by inhibiting GBM cell migration and altering the TME. Specifically, miR-124a-loaded exosomes (Exo-miR124a) suppress the clonogenicity of patient-derived GBM stem cells and reduce the tumor burden in intracranial xenograft models. Mechanistic studies have identified FOXA2 as a key target of miR-124a, linking its downregulation to apoptotic pathways and tumor suppression.^447^ Additionally, engineering GBM cells to express miR-302 and miR-367 profoundly affects the surrounding tumor environment, leading to decreased proliferation, reduced tumorigenicity, and the modulation of stemness markers in neighboring GBM cells.^448^ When implanted alongside GBM stem cells, these engineered cells significantly inhibited tumor growth in vivo.

In addition to miRNA-based approaches, exosome-mediated gene silencing has demonstrated efficacy in targeting oncogenic pathways in GBM. Studies have shown that exosomes engineered to carry a miR-21 sponge can effectively downregulate miR-21 while upregulating the expression of the tumor suppressors programmed cell death protein 4 and reversion-inducing cysteine-rich protein with Kazal motifs, key regulators of apoptotic and metastatic pathways. These effects have been validated in preclinical models, where modified exosomes suppressed tumor growth and enhanced the therapeutic response.^449^ Additionally, exosomes containing anti-miR-9 derived from hBMSCs, when combined with TMZ, significantly increased caspase activation and reduced GBM cell viability compared with those derived from TMZ alone, suggesting their potential to overcome chemoresistance.^450^ A landmark study using exosomes derived from rat bone marrow MSCs demonstrated their direct cytotoxic effects against GBM, indicating a shift from their traditional role as drug carriers to standalone therapeutic agents. These exosomes induced apoptosis in GBM cells and exhibited dose-dependent antitumor activity. Functional assays further revealed their ability to impair GBM cell migration and invasion, underscoring their potential in mitigating tumor progression and metastasis.^444^ Collectively, these findings highlight the growing importance of EV-based therapies in GBM treatment, providing a novel approach for targeted and personalized therapeutic interventions.

#### EVs as a drug delivery tool

EVs have emerged as a transformative drug delivery system for GBM therapy, offering a targeted and efficient approach to overcoming the challenges posed by the BBB and tumor resistance mechanisms. These vesicles efficiently transport chemotherapeutic agents such as DOX and PTX across the BBB, increasing drug accumulation within tumor cells while reducing systemic toxicity.^13^ Similarly, selumetinib-loaded EVs have demonstrated precise targeting capabilities, selectively delivering the drug to GBM cells while sparing healthy tissues, underscoring their potential for precision medicine.^451^ Methotrexate-loaded EVs modified with LDL and KLA peptides exhibited superior uptake in GBM spheroids,^452^ whereas yeast cytosine deaminase uracil phosphoribosyl transferase-engineered MSC EVs in combination with 5-FC effectively inhibited GBM growth.^453^ Neutrophil-derived EVs loaded with DOX demonstrated chemotactic migration toward tumor-infiltrating inflammatory cells, efficiently crossed the BBB and suppressed GBM progression.^454^ The adaptability of EVs for various administration routes, including intranasal and intraperitoneal delivery, further highlights their therapeutic flexibility.

In addition to conventional chemotherapy, EVs have been engineered to deliver novel therapeutic agents, including gene-editing tools and immunomodulatory molecules. EV-based systems integrating nanoparticle imaging agents with curcumin therapy have demonstrated dual functionality in GBM diagnosis and treatment, enhancing both detection and therapeutic outcomes.^455^ Their role in targeting GBM angiogenesis has also been explored, with miRNA-29a-3p-enriched MSC-derived EVs suppressing vasculogenic mimicry and angiogenesis independently of VEGF, suggesting a promising antiangiogenic strategy.^456^ Furthermore, DC-derived EVs loaded with dexamethasone exhibited immunomodulatory properties, promoting T cell activation and enhancing antitumor responses.^457^ Innovative surface modifications have further improved EV-based therapies. Arginylglycyl aspartic acid polypeptide-engineered EVs exhibit enhanced internalization into GBM cells, significantly improving drug delivery efficiency.^458^ Similarly, EVs loaded with small interfering RNAs targeting the FGFR3-TACC3 fusion gene effectively inhibited tumor cell viability while sparing adjacent normal tissues, demonstrating precision in gene-targeted therapy.^459^ Additionally, Cas9/sgRNA complexes encapsulated within Angiopep-2 (Ang)- and TAT-modified EVs achieved high-efficiency gene editing within GBM cells with minimal off-target effects,^460^ highlighting their potential in precision gene therapy.

Collectively, these advancements underscore EV-based therapies as novel and promising strategies for GBM treatment. Their ability to traverse the BBB, selectively target tumor cells and modulate the TME positions them as transformative tools for improving GBM outcomes. However, challenges such as optimizing targeting specificity, dosing, and long-term safety remain critical hurdles. Bridging the gap between experimental success and clinical application requires further research to establish standardized EV-based treatments, ultimately advancing personalized and effective GBM therapies.

### Nanoparticles

Nanoparticle-based therapies offer a promising strategy for GBM treatment by improving drug delivery, enhancing BBB penetration, and overcoming tumor resistance. Their ability to precisely target tumors while minimizing systemic toxicity has led to significant advancements. Curcumin in the nanomicellar form, combined with TMZ, reduces GBM cell invasion and modulates apoptotic and autophagy pathways.^461^ Aptamer-conjugated polyamidoamine dendrimer nanoparticles loaded with PTX and TMZ effectively suppressed tumors by decreasing autophagy and drug resistance.^462^ A nose-to-brain delivery system using nanoparticles conjugated with alpha-cyano-4-hydroxycinnamic acid and cetuximab has been shown to reduce tumor size by inhibiting EGFR activation.^463^ Transferrin-modified liposomes with cell-penetrating peptides significantly increase DOX and erlotinib transport across the BBB, leading to tumor cell apoptosis.^464^

Liposomal delivery systems integrating transferrin and penetrating peptides have further optimized receptor-mediated transcytosis, improving drug translocation and extending survival in GBM models.^465^ Codelivery of PTX and methotrexate via PLGA nanoparticles has outperformed free drug formulations. Chlorotoxin-conjugated PLGA nanoparticles effectively target and irradiate tumor cells, reducing ECM MMP-2 activity.^466^ When combined with radiation therapy, this approach results in increased nanovector accumulation and tumor suppression.^467,468^

The combination of gold nanoparticles with SI306 and radiotherapy improved tumor inhibition, while pH-sensitive polymersomes loaded with DOX exhibited excellent ability to cross the BBB.^469^ Magnetic nanoparticles loaded with camptothecin, TMZ, and indocyanine green have shown strong anti-GBM effects, as validated through imaging techniques.^470^ Composite microbowls that integrate curcumin, DOX, and amino acids have successfully delivered dual chemophotodynamic therapy, showing potential in 3D glioma spheroids.^471^ Gold–silver nanotriangles stabilized with polyethylene glycol have been demonstrated to be effective photothermal therapies, significantly reducing GBM cell viability with brief laser irradiation.^472^

Advanced nanotechnologies such as anti-EphA3-modified gold nanoparticles loaded with TMZ have been effective in overcoming TMZ resistance while enhancing photothermal therapy.^473^ A multifunctional phototheranostic agent incorporating dicysteamine-modified hypocrellin and cyclic peptides has enabled efficient tumor targeting via near-infrared absorption for chemo/photodynamic/photothermal therapy.^474^ Similarly, the indocyanine green-conjugated peptide AE105, which targets the urokinase plasminogen activator receptor, has improved the ability of fluorescence-guided surgery^475^ and photothermal therapy to prolong survival.^476^ Bradykinin aggregation-induced emission nanoparticles have shown high photothermal conversion efficiency, enabling deep-tissue tumor suppression and immune activation involving CD8^+^ T cells and NK cells.^477^ Gold nanorods conjugated with MCP-1 and iron-based frameworks significantly reduce the tumor volume after laser therapy.^478^ Immune-responsive nanoscale drug carriers, such as DOX-MSN-SS-iRGD&1MT nanoparticles, have been developed to codeliver chemotherapy and ICIs across the BBB.^479^ Compared with conventional DOX formulations, damage-associated molecular pattern-emitting nano-DOX formulations have exhibited superior immunogenicity, enhancing DC activation and CD8^+^ T cell responses in GBM. The administration of docetaxel-sHDL-CpG nanodiscs with radiotherapy has resulted in long-term tumor remission in GBM patients.^480^ Further innovations include Angiopep LipoPCB nanoparticles and poly(L-malic acid)-based nanoscale immunoconjugates, which have demonstrated enhanced BBB penetration and immune modulation.

Nanotechnology is advancing GBM treatment by integrating chemotherapy, photothermal therapy, immune modulation, and gene targeting. The ability of nanoparticles to cross the BBB, selectively target tumors, and stimulate immune responses highlights their clinical potential. Further research is needed to optimize formulations, reduce toxicity, and evaluate long-term efficacy to improve GBM therapy.

## Challenges and prospects in GBM therapy

**Fig. 8:**
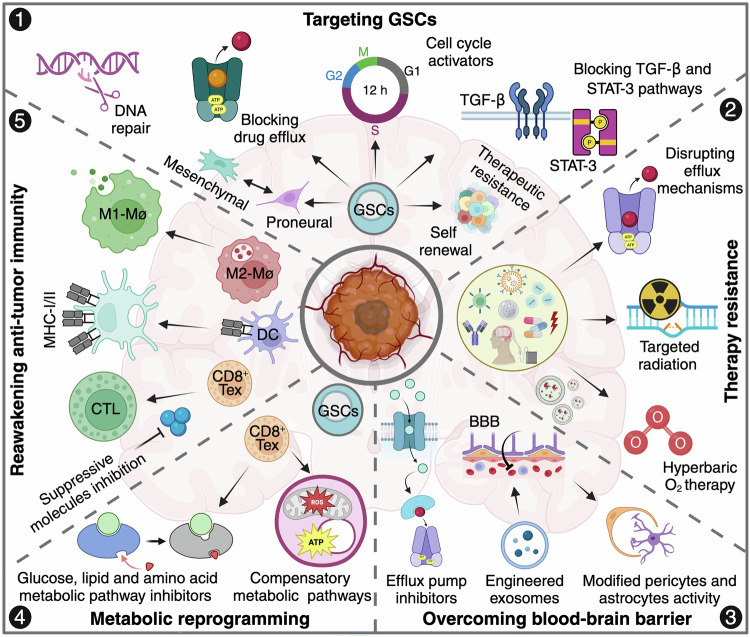
Major challenges and future therapeutic prospects in glioblastoma treatment. Key aspects, such as glioma stem cells (GSCs), therapy resistance, the blood‒brain barrier (BBB), metabolic reprogramming, and immune adaptation, are highlighted. **1.** The glioblastoma (GBM) tumor microenvironment (TME) contributes to therapy resistance and disease progression. GSCs exhibit self-renewal capacity and plasticity, driving tumor recurrence and treatment failure. The proneural-to-mesenchymal transition underscores the heterogeneity of GBM, further complicating treatment strategies. **2.** Therapy resistance mechanisms, including genetic mutations, epigenetic modifications, and adaptive survival pathways, are key obstacles to effective treatment. These mechanisms enable GBM cells to evade chemotherapy, radiotherapy, and targeted therapies. **3.** This study highlights the challenges of overcoming the BBB, which restricts drug penetration and limits the efficacy of systemic therapies. Prospects involve strategies such as engineered EV-mediated drug delivery, efflux pump inhibitors, and modified pericytes and astrocytes to increase therapeutic access to the tumor site. **4.** Metabolic reprogramming involves altered ATP production, lipid metabolism, and glycolysis, which provide energy for rapid tumor growth. Targeting metabolic vulnerabilities through the use of mitochondrial inhibitors, glycolysis modulators, and lipid metabolism disruptors is an emerging therapeutic approach. **5.** In GBM, tumor-associated macrophages (TAMs), regulatory T cells (Tregs), and exhausted CD8^+^ T cells (Tex) contribute to an immunosuppressive environment. Immunotherapy strategies, including checkpoint inhibitors, dendritic cell (DC)-based vaccines, and the reprogramming of macrophage phenotypes (M2 to M1), aim to restore antitumor immunity and improve therapeutic responses. This schematic underscores the multifaceted nature of GBM pathophysiology and emphasizes the need for multimodal approaches integrating targeted therapy, immunotherapy, metabolic intervention, and BBB-modulating strategies to increase treatment efficacy and improve patient outcomes

The treatment of GBM remains profoundly challenging in oncology because of the resilience and plasticity of GSCs, extensive tumor heterogeneity, the highly immunosuppressive TME, metabolic adaptability, and the BBB. Traditional therapies focused solely on cancer cell destruction often fail, as GSCs exploit β-catenin-mediated signaling pathways to evade apoptosis while simultaneously reinforcing immunosuppressive mechanisms that support tumor survival.^481^ Addressing these barriers requires a paradigm shift toward integrated molecular, immune, and metabolic interventions. The future of GBM therapy involves multifaceted strategies that incorporate immunotherapy, precision medicine, metabolic targeting, and advanced drug delivery systems to overcome resistance mechanisms and enhance treatment efficacy (Fig. 8).

### Targeting GSCs

GSC-driven resistance significantly contributes to tumor recurrence and therapeutic failure by regulating DNA repair mechanisms, promoting proneural-to-mesenchymal transition (PMT), and enhancing invasive pathways.^12^ Therapeutic targeting of these resistance mechanisms with PARP, ATR, and ATM inhibitors has been shown to increase radiosensitivity,^482^ whereas STAT3 and TGF-β inhibitors prevent PMT-driven resistance.^363^ Additionally, tumor invasion can be mitigated by disrupting adhesion molecules such as L1CAM and inhibiting matrix remodeling enzymes such as MMPs, thereby improving therapeutic outcomes. Pharmacological inhibition of β-catenin and Wnt signaling further disrupts GSC self-renewal, ultimately reducing tumor progression.

### Metabolic targeting

Metabolic reprogramming in GBM represents another key therapeutic target. Inhibiting glycolysis through GLUT1/3, HK2, and HIF-1α blockade, modulating lipid metabolism via FASN and SREBP-1 inhibitors, and disrupting amino acid metabolism via glutaminase (GLS) and SLC7A11 inhibitors have shown promise in limiting tumor growth.^483^ Exploiting IDH1/2 mutations with 2-HG inhibitors reverses metabolic and epigenetic dysregulation,^484^ whereas combination therapies incorporating metabolic inhibitors with standard treatments block metabolic plasticity and enhance therapeutic responses. The identification of compensatory metabolic pathways and the use of AI-driven analysis to predict resistance patterns further refine personalized treatment approaches.

### Overcoming the immunosuppressive TME

A critical limitation of immunotherapy in GBM is the highly immunosuppressive nature of the TME, which actively restricts T cell infiltration and function.^485^ A dysfunctional BBB exacerbates this issue by permitting the secretion of immunosuppressive cytokines such as TGF-β and IL-10 while promoting the accumulation of MDSCs and TAMs, both of which inhibit immune activation.^316^ To overcome these barriers, ICIs combined with TME-modulating agents, such as CSF-1R inhibitors and anti-TGF-β therapies, are being explored. However, clinical trials, including CheckMate-143, CheckMate-498,^486^ and CheckMate-548,^487^ have demonstrated limited efficacy, largely due to the low tumor mutational burden and adaptive immune resistance of GBM. These findings underscore the need for novel combination approaches that integrate epigenetic modulation and metabolic reprogramming to reinvigorate immune responses and improve therapeutic outcomes.

### Reinvigorating T cell exhaustion

**Fig. 9:**
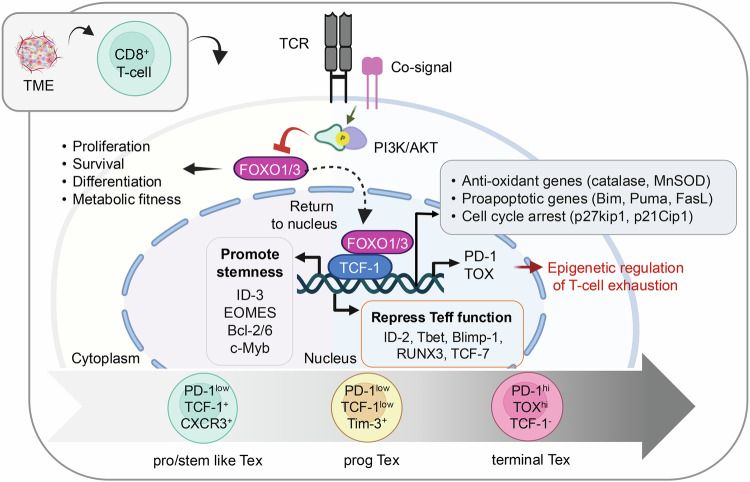
Transcriptional and epigenetic regulation and functional dynamics in CD8^+^ T cell exhaustion. The illustration depicts the intricate interplay between signaling pathways and transcriptional regulators that drive CD8^+^ T cell exhaustion in the TME. Key pathways include the PI3K/AKT signaling pathway and the modulation of the activity of FOXO1/3 transcription factors. In the nucleus, TCF-1 promotes stemness by upregulating genes such as *ID-3*, *EOMES*, *Bcl-2/6*, and *c-Myb*. Together with TCF-1, FOXO1/3 represses effector T cell (Teff) functions by regulating exhaustion-associated genes (*ID-2*, *Tbet*, *Blimp-1*, *RUNX3*, and *TCF-7*). Exhausted T cells progress through a continuum, transitioning from progenitor-like (pro/stem-like Tex) states (PD-1^low^, TCF-1^*+*^, and CXCR3^*+*^) to terminally exhausted (terminal Tex) states (PD-1^hi^, TOX^hi^, and TCF-1^*−*^). FOXO1/3 also govern antioxidant and proapoptotic genes and regulate cell cycle arrest genes, maintaining cellular integrity and upregulating PD-1 and TOX. PD-1 and TOX function as central mediators of epigenetic regulation, influencing chromatin accessibility and transcriptional programming to stabilize exhaustion phenotypes. This PD-1 epigenetic regulation shapes T cell function and metabolic fitness within the tumor microenvironment

The major limitation in GBM immunotherapy is T cell exhaustion, which results from chronic antigen exposure and leads to a progressive decline in CTL function. This exhaustion is driven by transcription factors such as FOXO1,^488^ FOXO3, and TOX and is further reinforced by epigenetic modifications involving DNMT3A, EZH2, and HBO1^489,490^ (Fig. 9). Addressing this issue through the use of FOXO1 modulators, PI3K/AKT inhibitors, and epigenetic therapies presents a promising strategy to restore T cell function and improve responsiveness to ICIs. Additionally, metabolic constraints within the TME, including glucose deprivation,^491^ amino acid competition,^492^ and lipid accumulation, further impair T cell activity. Strategies targeting these metabolic disruptions, such as GLUT1 inhibition, IDO blockade, and FASN inhibitors, have demonstrated potential in restoring T cell function.^493^ Emerging evidence also suggests that sodium chloride modulates T cell exhaustion by enhancing TCR signaling, metabolic fitness, and cytotoxicity,^494,495^ suggesting that sodium chloride is an innovative adjunct to existing immunotherapies.

### CAR-T cell therapy

**Fig. 10:**
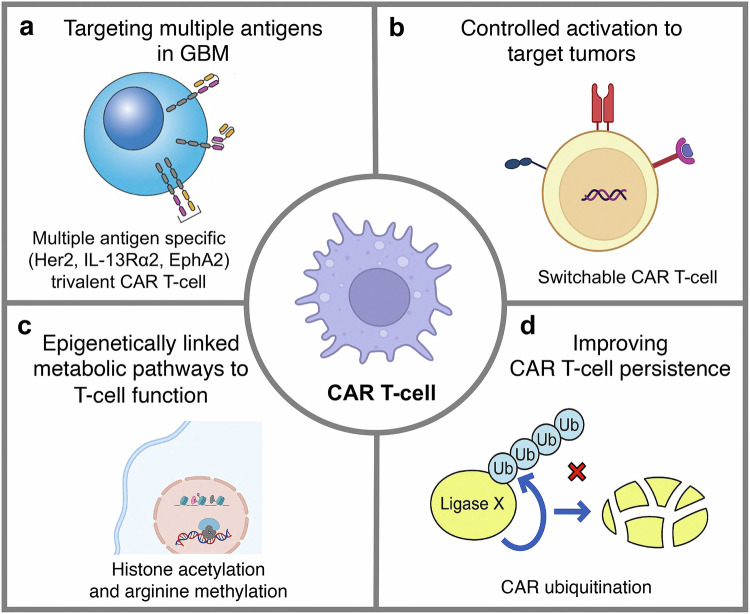
Increased therapeutic potential of CAR-T cells. The figure illustrates key strategies to optimize CAR-T cell therapy for GBM treatment. **a.** Multiantigen targeting improves CAR-T cell precision and efficacy against heterogeneous tumors. **b.** Advanced receptor designs, including costimulatory domain modifications and switch-controlled circuits such as synNotch CAR-T cells, enhance activation, persistence, and adaptability while sparing normal cells. **c.** Genome engineering introduces transcriptional and epigenetic changes to reduce exhaustion, improve memory, and increase cytokine production for sustained therapeutic effects. **d.** Inhibiting ubiquitin ligase-mediated degradation enhances CAR-T cell therapeutic potential

CAR-T cell therapy has emerged as a promising approach for GBM treatment, yet its efficacy is hindered by antigen heterogeneity and the immunosuppressive microenvironment. Optimizing CAR-T cell therapy requires multiple antigen-targeting strategies, such as dual- and trivalent CAR constructs directed against EGFRvIII, IL13Rα2, and EphA2, reducing the likelihood of immune escape.^496^ Advances in switch-controlled CAR-T cell systems, including synthetic Notch circuits, enable selective activation in high-antigen-density environments while minimizing off-target effects.^497^ Additionally, hypoxia-sensitive CAR-T cells, engineered to adapt to the oxygen-deprived microenvironment of GBM, offer a novel way to increase specificity while reducing systemic toxicity. Improving CAR-T cell persistence through metabolic engineering, including the modulation of SIRT1 and PRMT5, has also demonstrated promise in sustaining antitumor activity. Furthermore, engineering CAR-T cells to express chemokine receptors such as CCR6 enhances their ability to infiltrate the dense stromal architecture of GBM,^498^ improving overall treatment efficacy (Fig. 10).

### Overcoming the BBB

The BBB remains a formidable obstacle in GBM therapy, preventing efficient drug delivery and limiting the efficacy of systemic treatments. To overcome this barrier, advanced drug delivery systems such as nanoparticle-based carriers, focused ultrasound, and convection-enhanced delivery are being explored. Gene therapies utilizing CRISPR-based genome editing and OVs offer promising approaches for modifying the BBB or directly delivering therapeutic agents to tumor cells. Additionally, efflux pump inhibitors targeting P-glycoprotein and ABC transporters prevent premature drug elimination, whereas tumor vasculature normalization strategies enhance drug distribution.

### Drug delivery technology

EVs and ncRNAs are emerging as novel therapeutic tools for crossing the BBB in GBM therapy. Engineered EVs carrying therapeutic ncRNAs such as miRNAs and lncRNAs offer precise targeting of GSCs and the TME,^278^ although their clinical translation requires further validation and standardization. Electric field therapy, particularly TTF, has gained attention as a noninvasive strategy to disrupt mitotic processes in GBM cells and prolong patient survival.^353,354^ However, challenges such as tumor resistance and electrode placement issues necessitate further refinement. The next generation of dynamic dnEFTs aims to enhance immune modulation, reduce tumor resistance, and improve penetration for deep-seated tumors.^355^ Combining dnEFTs with immunotherapies and ferroptosis-inducing agents may amplify their therapeutic impact and increase their long-term efficacy.

### Precision medicine

Advances in precision medicine and adaptive therapy are reshaping GBM treatment by leveraging single-cell sequencing, AI-driven resistance prediction, and liquid biopsy technologies for real-time monitoring and personalized interventions. CRISPR-based genome editing and RNA interference technologies offer novel avenues for correcting oncogenic mutations and silencing tumor-promoting genes. AI-driven computational models optimize therapy selection and predict resistance mechanisms, facilitating more effective and tailored treatment regimens.

The future of GBM therapy lies in the seamless integration of diverse strategies targeting both tumor-intrinsic and microenvironmental resistance mechanisms. A comprehensive approach encompassing GSC eradication, immune reprogramming, metabolic modulation, CAR-T cell advancements, and innovative drug delivery technologies holds promise for improving GBM treatment efficacy. With continued research into synergistic treatment combinations, the translation of novel scientific advancements into effective clinical interventions offers new hope for prolonged survival and improved quality of life for GBM patients.

## Conclusion

Despite significant advancements in understanding GBM pathogenesis, effective treatments remain elusive because of tumor heterogeneity, adaptability, and complex interactions with the TME. Future research must prioritize novel drug combination therapies that simultaneously target multiple oncogenic pathways, disrupting the adaptive mechanisms of GBM and overcoming therapeutic resistance. Personalized and precision medicine offers promising strategies by integrating genomic, transcriptomic, metabolomic, and epigenomic insights with biomarker-driven treatment selection and AI-powered predictive models. These approaches optimize treatment regimens by identifying patient-specific vulnerabilities. However, challenges such as biomarker validation, refining treatment paradigms, and ensuring accessibility to advanced diagnostics must be addressed to realize their full clinical potential. Immunotherapy holds great promise but faces barriers such as T cell exhaustion, checkpoint inhibitor resistance, and antigenic heterogeneity. Future directions should focus on reprogramming the immunosuppressive TME, enhancing T cell infiltration and function, and developing next-generation immunotherapies. Innovations such as improved CAR-T cell designs, OV-based therapies, and mRNA-based cancer vaccines combined with metabolic and epigenetic modifications may significantly increase immune responses. Additionally, overcoming key obstacles such as the BBB and drug efflux is crucial for improving drug delivery and minimizing tumor recurrence. Emerging technologies, including focused ultrasound, nanomaterial-based drug carriers, and electric field therapy, offer novel solutions to enhance therapeutic penetration and efficacy. The integration of precision therapeutics, molecular targeting, immunomodulation, and metabolic interventions provides a comprehensive framework for tackling GBM. Interdisciplinary collaboration and innovative clinical trial designs will be vital in translating these scientific advances into transformative clinical interventions. By leveraging these innovative strategies, the field has moved closer to achieving significant improvements in survival and quality of life for GBM patients.

## Supplementary information

Supplementary Materials
