# Supplementary material for: Glioblastoma at the crossroads: current understanding and future therapeutic horizons
Source: Signal Transduct Target Ther. 2025 Jul 9;10:213. doi: 10.1038/s41392-025-02299-4 (PMC12238593; doi:10.1038/s41392-025-02299-4)
Supplement: Supplementary file 1 — Supplementary Materials [file 41392_2025_2299_MOESM1_ESM.docx]

Supplementary Materials for

**Glioblastoma at the crossroads: current understanding and future therapeutic horizons**

Shilpi Singh^1^, Devanjan Dey^2^, Debashis Barik^3^, Iteeshree Mohapatra^4^, Stefan Kim^1^, Mayur Sharma^1^, Sujata Prasad^5^, Peize Wang^1^, Amar Singh^2^*, and Gatikrushna Singh^1^*

Correspondence to: gsingh@umn.edu

**This PDF file includes:**

Tables S1 to S9

List of table titles:

1. Table S1: Clinical grading and molecular subtypes of GBM.
2. Table S2: Circular RNA as a prognostic and diagnostic biomarkers of GBM.
3. Table S3: Circular RNA mediated GBM pathogenesis.
4. Table S4: Long noncoding RNA as biomarkers in GBM.
5. Table S5: Long noncoding RNAs associated with GBM pathogenesis.
6. Table S6: micro RNAs associated with GBM pathogenesis.
7. Table S7: Extracellular vesicles as a biomarker for GBM diagnosis.
8. Table S8: Clinical investigations of vaccines against GBM.
9. Table S9: Oncolytic virus in clinical trial for GBM treatment.

**Other Supplementary Materials for this manuscript include the following:**

References cited in the tables S1-S9.

**Table S1. Clinical grading and molecular subtypes of GBM.**

| **Grading** | **Circumscript** | **Diffuse** | | | **References** |
| --- | --- | --- | --- | --- | --- |
|  | WHO grade-I (G1) | WHO grade-II (G2) | WHO grade-III  (G3) | WHO grade-IV  (G4) |  |
|  |  | Low grade | High grade | |  |
| Characteristics | Benign, slow growing tumor  associated with  long-term survival and  amenable to  surgical resection | Increased hypercellularity,  no vascular proliferation,  no mitosis and  no necrosis | High rate of hypercellularity,  high rate of mitosis,  high rate of  tumor recurrence,  no necrosis and  no vascular proliferation | Very high rate of hypercellularity, mitosis, presence of vascular proliferation,  presence of necrosis and nuclear atypia | ^1,2^ |
| Types | Pilocystic and  subependymal  astrocyotoma | Astrocytoma (IDH-mutant) and  oligodendroglioma  (IDH-mutant and 1p/19q-codeleted) | Anaplastic astrocytoma (IDH-mutant) and  anaplastic oligodendroglioma  (IDH-mutant and 1p/19q-codeleted) | GBM (IDH- wild-type),  GBM (IDH-mutant) and diffuse midline glioma (H3K2M mutant) |  |
| **Molecular subtypes of GBM** | | | | | |
| GBM subtypes | Proneuronal | Mesenchymal | Classical | Neural | ^3–6^ |
| Neural cell type | Oligodendrocyte | Astroglial | Murine astrocytes | Neuron, oligodendrocytes  and astrocytes |  |
| Markers | SOX, DCX, DLL3, ASCL1, TCF4, PDGFRA, NKX2-2, OLIG2 PDGFRA | CD44, MERTK | NES, NOTCH3, JAG1, LFNG, SMO, GAS1, GLI2 | NEFL, GABRA1, SYT1 and SLC12A5 |  |
| Alteration/ mutations | Alternation and  IDH1 point mutation | Lower NF1 expression, NF1, PTEN co-mutation and high expression of TRADD, RELB,  TNFRSF1A | Chromosome 7 amplification paired with chromosome 10 loss, high EGFR amplification, EGFR point or vIII mutation and lack of p53 mutation | Neuron projection, axon and synaptic transmission |  |

**Table S2. Circular RNA as a prognostic and diagnostic biomarkers for GBM.**

| **Circular RNA** | **Sample** | **Biomarker** | **Expression** | **References** |
| --- | --- | --- | --- | --- |
| circ_0067934 | Tissue | Prognostic | Upregulation | ^7^ |
| circ-SMO |  |  | Upregulation | ^8^ |
| circ-SKA3 |  |  | Upregulation | ^9^ |
| circ-SERPINE2 |  |  | Upregulation | ^10^ |
| circ_0001588 |  |  | Upregulation | ^11^ |
| circ-LGMN |  |  | Upregulation | ^12^ |
| circ-MTO1 |  |  | Downregulation | ^13^ |
| circ-ASAP1 |  |  | Upregulation | ^14^ |
| circ_0074027 |  | Predictive | Upregulation | ^15^ |
| circ_0006168 |  |  | Downregulation | ^16^ |
| circ-ENTPD7 |  | Predictive  Prognostic | Upregulation | ^17^ |
| circ_0029426 |  |  | Upregulation | ^18^ |
| circ-FLNA |  |  | Upregulation | ^19^ |
| circ-NF1 |  |  | Upregulation | ^20^ |
| circ-EPB41L5 |  |  | Downregulation | ^21^ |
| circ-FBXW7  circ-FBXW7-185aa |  | Prognostic | Downregulation | ^22^ |
| circ_005019  circ_0000880  circ_0051680  circ_0006365 | Serum | Prognostic | Upregulation | ^23^ |
| circ-WDR62 | Exosome | Prognostic | Upregulation | ^24^ |

**Table S3. Circular RNA mediated GBM pathogenesis.**

| **Circular RNA** | **Expression** | **Function** | **Mechanism** | | **Target** | **References** |
| --- | --- | --- | --- | --- | --- | --- |
| circ-NT5E | Upregulation | Sponging for miR-422a | | Inhibits apoptosis,  promote migration, invasion and proliferation | SOX4/PI3KCA | ^25^ |
| circ-HIPK3 | Upregulation | Sponging for miR-465 | | Promote tumorigenesis, invasion and proliferation | IGF2/PI3K/AKT | ^26^ |
| circ_0037655 | Upregulation | Sponging for miR-214 | | Induce invasion and viability | PI3K | ^27^ |
| circ-SHKBP1 | Upregulation | Sponging miR-544a/miR-37 | | Increase angiogenesis | PI3K/AKT | ^28^ |
| circ_0000215 | Upregulation | Sponging miR-495-3p | | Suppression of apoptosis | CXCR2/PI3K/AKT | ^29^ |
| circ_0000177 | Upregulation | Sponging miR-638 | | Promote proliferation and invasion | Wnt | ^30^ |
| circ-TTBK2 | Upregulation | Sponging miR-217 | | Suppress apoptosis,  induce migration, invasion and proliferation | MAPKs | ^31^ |
| circ_0043278 | Upregulation | Sponged miR-638 | | Induce invasion, migration and proliferation | Wnt | ^32^ |
| circ-PIP5K1A | Upregulation | Sponging miR-515-5p | | Increase EMT, invasion, proliferation and  inhibit apoptosis | TCF12 and PI3K/AKT | ^33^ |
| circ_0082374 | Upregulation | Sponging miR-326 | | Increase glycolysis, proliferation and metastasis | Wnt/β-catenin | ^34^ |
| circ-PITX1 | Upregulated | Sponging miR-379-5p | | Increase proliferation and  suppress apoptosis | MAPKs | ^35^ |
| circ_0001730 | Upregulated | Sponged miR-326 | | Increase proliferation and migration | Wnt/β-catenin | ^36^ |
| circ-ASAP1 | Upregulated | Sponging miR-502-5p | | Inhibit apoptosis and  induce proliferation | MAPKs | ^14^ |
| circ-ARF1 | Upregulated | Sponging miR-342-3p | | Increase angiogenesis | ERK | ^37^ |
| circ-MAPK4 | Upregulated | Sponging miR-125a-3p | | Increase survival and reduce apoptosis | MAPKs | ^38^ |
| circ-ATXN1 | Upregulated | Sponging miR-526b-3p | | Induce angiogenesis | - | ^39^ |
| circ_0046701 | Upregulated | Sponging miR-142-3p | | Increase invasion and proliferation | - | ^40^ |
| circ-PARP4 | Upregulated | Sponging miR-125a-5p | | Induce metastasis, invasion and migration | - | ^41^ |
| circ-UBAP2 | Upregulated | Sponging miR-1205 and miR-382 | | Reduce apoptosis,  induce proliferation, invasion and migration | - | ^42^ |
| circ_0067934 | Upregulated | - | | Induce metastasis and proliferation, | PI3K/AKT | ^43^ |
| circ_0008344 | Upregulated | - | | Reduce apoptosis,  induce proliferation, invasion and migration | - | ^44^ |
| circ-EPB41L5 | Downregulated | Sponging miR-19a | | Suppress invasion, proliferation and migration | circ-EPB41L5/miR19a/EPB41L5/p-AKT | ^21^ |
| circ-SMARCA5 | Downregulated | - | | Reduce angiogenesis | - | ^45^ |
| circ-AKT3 | Downregulated | - | | Reduce radio-resistance, tumorgenicity and proliferation | PI3K/AKT | ^46^ |

**Table S4. Long non-coding RNA as biomarkers in GBM.**

| **Long non-coding RNA** | **Source** | **Status** | **Expression** | **References** |
| --- | --- | --- | --- | --- |
| ANRIL | Serum | Upregulated | Tumor grading and prognosis | ^47^ |
| GAS5 | Serum | Upregulated | Prognosis | ^48^ |
| HOTAIR | Serum | Upregulated | Prognosis | ^49,50^ |
| linC00565 | Serum | Upregulated | Prognosis | ^51^ |
| linC00641 | Serum | Upregulated | Prognosis | ^51^ |
| MIR210HG | Serum | Upregulated | Diagnosis | ^52^ |
| SAMMSON | Plasma | Upregulated | Diagnosis | ^53^ |

**Table S5. Long non-coding RNAs associated with GBM pathogenesis.**

| **Long non-coding RNA** | **Expression** | **miRNA** | **Mechanism** | **Target** | **References** |
| --- | --- | --- | --- | --- | --- |
| ATB | Upregulated | miR-200a | Proliferation, migration and invasion | TGF-β2 | ^54^ |
| ECONEXIN | Upregulated | miR-411-5p | Proliferation | TOP2A | ^55^ |
| FTX | Upregulated | miR-342-3p | Proliferation and invasion | AEG1 | ^56^ |
| MIR155HG | Upregulated | miR-155-3p  miR-155-5p | Proliferation | PCDH7, PCDH9, Wnt/β-catenin | ^57^ |
| TP73-AS1 | Upregulated | miR-142 | Proliferation and invasion | HMGB1 | ^58^ |
| TUG1 | Upregulated | miR-144 | Angiogenesis | HSF2, ZO-1, OCLN, CLDN5 | ^59^ |
| HOXA11-AS | Upregulated | miR-214-3p | Proliferation, invasion, migration and apoptosis | EZHZ | ^60^ |
| CCAT1 | Upregulated | miR-181b  miR-410 | Proliferation | FGFR3, PDGFRA | ^61^ |
| HOTAIR | Upregulated | miR-148b-3p  miR-326 | Proliferation, invasion and migration | ZO-1, OCLN, CLDN5, USF1  FGF1, PI3K/AKT, MEK1/2 | ^62^ |
| MALAT1 | Upregulated | miR140  miR-101 | Proliferation, chemosensitivity, BTB permeability, migration and autophagy | Nuclear factor YA, STMN1, RAB5A, ATG4D | ^63,64^ |
| PVT1 | Upregulated | miR-488-3p  miR-186  miR-190a-5p | Proliferation, migration, invasion, angiogenesis,  cell cycle and apoptosis | MEF2C, ATG7, Beclin1, EZH2, JAGGED1 | ^65^ |
| UCA1 | Upregulated | miR-122  miR-182 | Proliferation, migration, invasion and apoptosis | iASPP | ^66–68^ |
| Xist | Upregulated | miR-137  miR-152  miR-429  miR-29c | Proliferation, migration, invasion, apoptosis, angiogenesis and chemosensitivity | RAC1, XCR7, ZO-2, FOXC1,  MGMT, SP1 | ^69–71^ |
| H19 | Upregulated | miR-140  miR-675  miR-29a | Proliferation and  angiogenesis | iASPP, CDK6,  VASH2 | ^72,73^ |
| NEAT1 | Upregulated | miR-181d-5p  miR-107  miR-449b-5p  let-7e  miR-132 | BTB permeability, proliferation, invasion, migration and apoptosis | SOX5, CDK6,  c-MET, PI3K/AKT/mTOR, MEK/ERK, SOX-2 | ^74,75^ |
| EGFR-AS1 | Upregulated | miR-133b | Migration, invasion and apoptosis | RACK-1 | ^76^ |
| CCDC26 | Upregulated | miR-203 | Proliferation and migration | - | ^77^ |
| HOXA11-AS3 | Upregulated | miR-140-5p | Proliferation | - | ^78^ |
| AB073614 | Upregulated | - | Proliferation, invasion, migration and apoptosis | PI3K/AKT,  E-cadherin, Vimentin | ^79^ |
| CCAT2 | Upregulated | - | Proliferation, cell cycle progression and migration | Wnt/β-catenin | ^80^ |
| CCND2-AS1 | Upregulated | - | Proliferation | Wnt/β-catenin | ^81^ |
| FOXD3-AS1 | Upregulated | - | Proliferation, migration and invasion | FOXD3 | ^82^ |
| Linc-OIP5 | Upregulated | - | Proliferation and migration | YAP, Notch | ^83^ |
| ZFAS1 | Upregulated | - | Proliferation, migration, invasion and disease prognosis | Notch | ^84^ |
| ZEB1-AS1 | Upregulated | - | Proliferation, invasion, migration and apoptosis | CycD1, CDK2 ZEB1, MMP2, MMP9, CDH2, Integrin-β1 | ^85^ |
| CRNDE | Upregulated | - | Proliferation, invasion, migration and apoptosis | mTOR | ^86,87^ |
| HOTAIR | Upregulated | - | Angiogenesis  Proliferation, invasion, migration and apoptosis | BRD4, VEGF-A | ^88^ |
| HULC | Upregulated | - | Proliferation invasion, migration, angiogenesis and adhesion | Survivin, c-MYC, CycA/D1/E, p-Rb, Skp-1/2, CDK2/4, EZH2, Bcl-2/Bax, caspase-3/8 | ^89^ |
| MALAT1 | Upregulated | - | Proliferation and apoptosis | CCND1, MYC | ^90^ |
| UCA1 | Upregulated | - | Proliferation | CycD1 | ^67^ |
| HMMR-AS1 | Upregulated |  | Tumor growth and radio-resistance | ATM, RAD51, BMI1 | ^91^ |
| MVIH | Upregulated | - | Proliferation, migration and invasion | - | ^92^ |
| MIR210HG | Upregulated | - | Proliferation | - | ^93^ |
| FER1L4 | Upregulated | - | Proliferation, invasion and apoptosis | - | ^94^ |
| POU3F3 | Upregulated | - | Proliferation and angiogenesis | - | ^95^ |
| SPRY4-IT1 | Upregulated | - | Proliferation, invasion, migration and apoptosis | - | ^96^ |
| PVT1 | Upregulated | - | Patient survival, chemotherapy and radiotherapy | - | ^94^ |
| HOXA11-AS3 | Upregulated | - | Proliferation | - | ^97^ |
| NEAT1 | Upregulated | - | Proliferation, invasion and migration | - | ^98^ |
| TP53TG1 | Upregulated | - | Proliferation, invasion, migration and apoptosis |  | ^99^ |
| CASC2 | Downregulated | miR-181a  miR-193-5p  miR-21 | Proliferation, chemoresistance, migration, invasion and apoptosis | PTEN, mTOR | ^100^ |
| MALAT1 | Downregulated | miR-155  miR-203 | Proliferation, survival and  chemoresistance | FBXW7, TS | ^101^ |
| MEG3 | Downregulated | miR-19a | Proliferation and apoptosis | PTEN | ^102^ |
| TUSC7 | Downregulated | miR-23b | Proliferation, apoptosis and survival | TUSC7 | ^103^ |
| TUG1 | Downregulated | miR-26a | Proliferation and apoptosis | PTEN | ^104^ |
| ADAMTS9-AS2 | Downregulated | - | Proliferation, migration, invasion and survival | ADAMTS9, DNMT1 |  |
| CASC2 | Downregulated | - | Proliferation | β-catenin, CycD1,  c-MYC | ^105^ |
| H19 | Downregulated | - | Chemoresistance | MDR, MRP, ABCG2 | ^106^ |
| HOTTIP | Downregulated | - | Proliferation and apoptosis | BRE, CycA, CDK2, p53 | ^107^ |
| lnc00462717 | Downregulated | - | Proliferation, migration and apoptosis | MDM2 | ^108^ |
| MALAT1 | Downregulated | - | Proliferation | Ki-67, MMP2, MAPK | ^109^ |
| MDC1-AS | Downregulated | - | Proliferation | MDC1, CycB1/CDK2 | ^95^ |
| TSLC1-AS1 | Downregulated | - | Proliferation, migration and invasion | TSLC1 | ^110^ |
| TUG1 | Downregulated | - | Proliferation and apoptosis | BCL-2, CASP3, CASP9 | ^111^ |

**Table S6. microRNAs associated with GBM pathogenesis.**

| **microRNA** | **Expression** | **Targets** | **Function** | **References** |
| --- | --- | --- | --- | --- |
| miR-21 | Upregulated | TNFR,  caspase-3,  caspase-9, APAF1 | Apoptosis | ^112^ |
|  |  | Alter Bax/Bcl-2 ratio | Chemoresistance | ^113^ |
|  |  | IGFBP3, RECK, TIMP3, VEGF | Migration and invasion | ^114–116^ |
|  |  | Bmpr2/Btg2 | Angiogenesis | ^117^ |
|  |  | PTEN | Immune suppression | ^118^_,_^119^ |
| miR-221/222 | Upregulated | p27, p57 | Induce proliferation and  reduce cell death | ^120^ |
|  |  | DNM3 | Chemoresistance | ^121^ |
| miR-221-3p/  miR-222-3p | Upregulated | FAT1,  PDCD10, PTEN/PUMA | Clonogenicity, migration and invasion | ^122^ |
| miR-125b | Upregulated | 3’-UTR of Bmf | Tumor progression | ^123^ |
| miR-141-3p | Upregulated | p53 | Tumor progression and chemoresistance | ^112^ |
| miR-25 | Upregulated | Increasing expression of CADM2 | Migration, invasiveness and tumor progression | ^124^ |
| miR-25 | Upregulated | MDM2, NEFL | Tumor progression | ^125^ |
| miR-210-3P | Upregulated | Alter mitochondrial membrane potential, improved function | Tumor progression and EMT | ^126^ |
| miR-93 | Upregulated | Integrin β8 | Tumor progression, migration, invasion and chemoresistance | ^127^ |
|  |  | ITGB8 | Tumor progression and angiogenesis | ^128^ |
| miR-155-3p | Upregulated | PCDH7, SIX1 | Tumor progression immune evasion and chemoresistance | ^129^ |
| miR-155-5p | Upregulated | ACOT12 | Migration and invasion | ^130^ |
| miR-26a | Upregulated | AP-2α/Nanog | Tumor progression | ^131^ |
| miR-33a | Upregulated | PKA/Notch | Tumor progression | ^132^ |
|  |  | PDE8A, PDL1, PTEN, UVRAG | Tumor progression and GSC self-renewal | ^133^ |
| miR-300 | Upregulated | LZTS2 | Tumor progression and GSC self-renewal | ^134^ |
| miR-182 | Upregulated | BCL2L12, CYLD, HIF2A, MET, PCDH8, STAT3 | Tumor progression invasion and chemoresistance | ^135,136^ |
| miR-210-3p | Upregulated | HIF3A, ISCU | Tumor progression, migration and chemoresistance | ^137^ |
| miR-10b | Upregulated | APAF1, BCL2L11, CDH1, CDKN1A, CDKN2A, CYLD, FOXO3, HOXD10, NOTCH1, PAX6, PTCH1, PTEN, TFAP2C, p53 | Tumor progression migration, invasion and EMT | ^138–142^ |
| miR-10b-5p | Upregulated | TFAP2A | Migration and invasion | ^143^ |
| miR-9 | Upregulated | MYC, OCT4 | Angiogenesis | ^144^ |
| miR-1238 | Upregulated | CAV-1 | Chemoresistance | ^145^ |
| miR-301a | Upregulated | TCEAL7 | Chemoresistance | ^146^ |
| miR-603 | Upregulated | IGF1, IGF1R | Radio-resistance | ^147^ |
| miR-27b-3p | Upregulated | MLL4/PRDM1 | Stemness | ^148^ |
| miR-26a | Upregulated | PTEN | Angiogenesis | ^149^ |
| miR-148a-3p | Upregulated | ERRFI1 | Angiogenesis | ^150^ |
| miR-148a | Upregulated | CADM1 | Migration and invasion | ^151^ |
| miR-182-5p | Upregulated | KLF2, KLF4 | Angiogenesis | ^152^ |
| miR-451 | Upregulated | c-MYC | Immune suppression | ^153^ |
| miR-10a | Upregulated | Rora | Immune suppression | ^119^ |
| miR-92a | Upregulated | Prkar1a | Immune suppression | ^154^ |
| miR-214-5p | Upregulated | CXCR5 | Immune suppression | ^155^ |
| miR-1246 | Upregulated | TERF2IP/ DUSP3 | Immune suppression | ^143^ |
|  |  | FAK | Migration and invasion | ^143^ |
| miR-7239-3p | Upregulated | Bmai-1 | Tumor progression | ^156^ |
| miR-29a | Upregulated | Hbp1 | Immune suppression | ^154^ |
| miR-1587 | Upregulated | NCOR1 | Tumor progression | ^157^ |
| miR-301a | Upregulated | PTEN | Tumor progression | ^158^ |
| miR-H6-3p | Upregulated | EPB41L1, SH3PXD2A | Tumor progression | ^159^ |
| miR-134 | Downregulated | PI3K/AKT | Tumor progression, apoptosis and EMT | ^160^ |
| miR-34a | Downregulated | c-MET RTK, Notch-1, Notch-2, CDK6, CCND1, SIRT1 | Tumor progression | ^161^ |
|  |  | BCL2, CDK6, MET, MSI1, NOTCH1, NOTCH2, PDGFRA, RICTOR, SIRT1, SMAD4, WNT6, YY1 | Tumor progression and chemoresistance | ^162–165^ |
| miR-9 | Downregulated | FOXG1, CAMTA1, CREB, FOXP2 | Tumor progression | ^166^ |
|  |  | JAK1, JAK2, JAK3, LDHA, MAPK14, MAPKAP3, NF1, PTCH1, S1PR1, SMC1A, SOX2, STMN1 | Tumor progression, migration, invasion, aerobic glycolysis, chemoresistance and EMT | ^167–169^ |
| miR-186 | Downregulated | Inhibition of SMAD6 | Apoptosis | ^170^ |
| miR-142-3p | Downregulated | Inactivated Wnt/β-catenin, activated caspase-3 | Tumor progression and migration | ^171^ |
| miR-379-5p | Downregulated | MGST1 modulation | Migration, invasion and EMT | ^172^ |
| miR-665 | Downregulated | Wnt/β-catenin inhibition | Migration and invasion | ^173^ |
| miR-296 | Downregulated | EAG1 | Invasion and multidrug resistance | ^174^ |
| miR-320a | Downregulated | Aquaporin 4 | Invasion and migration | ^175^ |
| miR-125b | Downregulated | MAZ, VEGF | Angiogenesis and tumor progression | ^176,177^ |
|  |  | Mitochondrial apoptosis | Chemoresistance | ^178^ |
| miR-124-3p | Downregulated | PI3K/AKT/NF-κB | Tumor progression, invasion and migration | ^179^ |
|  |  | AURKA, FOSL2, RHOG, SOS1 | Tumor progression, invasion and chemoresistance | ^180–182^ |
| miR-128,  miR-149 | Downregulated | RAP1B | Invasion and  chemoresistance | ^183^ |
| miR-181a/b/c/d | Downregulated | RAP1B/CDC42, RhoA/N-cadherin | Chemoresistance and tumor progression | ^184^ |
|  |  | FBXO11, MGMT, RAP1B, SP1 | Migration, invasion and chemoresistance | ^184–186^ |
| miR-195 | Downregulated | CCNE1 | Chemoresistance | ^187^ |
| miR-524-5p  miR-324-5p | Downregulated | EZH2 | Tumor progression and chemoresistance | ^152^ |
| miR-137 | Downregulated | RTVP-1 | GSC elf-renewal | ^188^ |
| miR-30a | Downregulated | AKT, BDNF | GSC self-renewal | ^189^ |
|  |  | BECN1 | Chemoresistance |  |
| miR-30c | - | JAK–STAT | Neural differentiation | ^190^ |
| miR-29a | Downregulated | MDM2/4 | Tumor progression, migration and invasion | ^191^ |
|  |  | CDC42, DNMT3A, DNMT3B, PDGFA, PDGFC, QKI-6, TRAF4 | Tumor progression migration and invasion | ^192–195^ |
| miR-101-3p | Downregulated | GSK3B, PTGS2, SOX9, TRIM44 | Tumor progression, invasion, migration, metastasis and chemoresistance | ^196^ |
| miR-128-3p | Downregulated | BMI1, E2F3, MET, PDGFRα, PTGS2 | Tumor progression, migration, invasion and chemoresistance | ^197^ |
| miR-142-3p | Downregulated | AKT1, HMGA2, IL6, MGMT, RAC1 | Tumor progression, migration, invasion, chemoresistance and immunosuppression | ^198,199^ |
| miR-146a-5p | Downregulated | NOTCH1, POU3F2, SMARCA5 | Tumor progression and stemness | ^200,201^ |
| miR-146b-5p | Downregulated | EGFR, MMP16, TRAF6 | Tumor progression, migration and invasion | ^202–204^ |
| miR-7 | Downregulated | EGFR, FAK, IRS2, OGT, RAF1, SATB1, TBX2, YY1 | Tumor progression, migration, invasion, angiogenesis and EMT | ^184,205,206^ |
| miR-4524b-5p | Downregulated | ALDH1A3, PI3K/AKT/mTOR | Radio-resistance and tumor progression | ^207^ |

**Table S7. Extracellular vesicles as a biomarker for GBM diagnosis.**

| **EV Source** | **Isolation method** | **Biomarkers** | **Status** | **Identification method** | **References** |
| --- | --- | --- | --- | --- | --- |
| Blood | Microfluidic chip | IDH1 | Upregulated | RT-qPCR and nested PCR | ^208^ |
|  | Ultracentrifugation | TrkB | Upregulated | Western blotting | ^209^ |
|  |  | PTRF | Upregulated | RT-qPCR | ^210^ |
|  |  | INFγ, IL10, IL13, CD80, CD86, ICOSL | Downregulated | Cytokine assay and  ELISA | ^211^ |
|  |  | NLGN3, PTTG1, EGFR | Upregulated | Flow cytometry and RT-qPCR | ^212^ |
|  |  | FASN | Upregulated | RT-qPCR | ^213^ |
|  |  | IDH1(G395A), gDNA | Upregulated | PCR | ^214^ |
|  |  | miR-210 | Upregulated | RT-qPCR | ^215^ |
|  | Dielectrophoresis | GFAP,  TAU | Upregulated | DEP-immunofluorescence | ^216^ |
|  | Size exclusion  chromatography | SDC1 | Upregulated | LC-MS/MS and  ELISA | ^217^ |
|  | ExoQuick | RN-U6,  miR-320,  miR-574-3P | Upregulated | Microfluidic TLDA and miRNA array | ^218^ |
|  |  | miR-29b | Upregulated | RT-qPCR | ^219^ |
|  |  | miR-301-a | Upregulated | RT-qPCR | ^158^ |
|  |  | RN-U6–1 | Upregulated | Digital droplet PCR | ^220^ |
|  |  | LINC00470 | Upregulated | RT-qPCR | ^221^ |
|  | ExoQuick-TC | miR-21, miR-222, miR-124–3p | Upregulated | RT-qPCR | ^222^ |
|  | Total Exosome  Isolation reagent | lnc-HOTAIR | Upregulated | RT-qPCR | ^223^ |
|  |  | circ-METRN | Upregulated | RT-qPCR | ^224^ |
|  |  | miR-454-3p | Upregulated | RT-qPCR | ^225^ |
|  | qEV (SEC + CD44 based  isolation) | miR-15b-3p, miR-21-3p, miR155-5p, let-7a-5p | Upregulated | RT-qPCR | ^226^ |
|  | Ultracentrifugation | RPL11, RPS12, TMSL3, B2M | Downregulated | RT-qPCR and microarray | ^227^ |
|  | ExoQuick | miR-766-5p, miR-376b-5p | Downregulated | Small RNA sequencing |  |
|  | Total Exosome  isolation reagent | EGFRvIII, EGFR | - | Semi-nested PCR | ^228^ |
| CSF | Ultracentrifugation | miR-21, | Upregulated | RT-qPCR | ^216^ |
|  |  | EGFR, EGFRvIII | Upregulated | RT-qPCR | ^229^ |
|  | Filtration | LGALS9 | - | LC-MS | ^230^ |
| Blood, CSF | Ultracentrifugation | Mutant-IDH1 | Upregulated | BEAMing and  Digital droplet PCR | ^231^ |
|  |  | miR-21 | Upregulated | RT-qPCR | ^232^ |
|  |  | PD-L1 DNA | Upregulated | Digital droplet PCR | ^213^ |
| Blood, CSF, | Total exosome isolation reagent | miR-182-5p | Upregulated | RT-qPCR | ^233^ |
| CUSA fluid | Ultracentrifugation | TCP1, CCT2, CCT5, CCT6A, CCT7 | - | LC-MS/MS | ^234^ |
|  |  | miRNAs, piRNAs | - | Small RNA sequencing | ^235^ |

**Table S8. Clinical investigations of vaccines against GBM.**

| **Vaccine/combinations** | **Combination** | **Clinical trial identifier** | **Phase** | **Status** |
| --- | --- | --- | --- | --- |
| EGFRvIII peptide | TMZ | NCT00458601 | II | Not recruiting |
| EGFRvIII peptide | TMZ | NCT01480479 | III | Completed |
| EGFRvIII peptide | DI-TMZ | NCT00643097 | II | Completed |
| CMV pp65 DC | DI-TMZ | NCT00639639 | I | Completed |
| DCVax-L | - | NCT00045968 | III | Active, not recruiting |
| CMV pp65 DC + 111In-labeled DC + Toxoid | Basiliximab | NCT02366728 | II | Completed |
| HSPPC-96 | TMZ | NCT00905060 | II | Completed |
| ICT-107 (autologous DC pulsed with synthetic peptides mimicking GAAs) |  | NCT01280552 | II | Completed |
| HSPPC-96 peptide | - | NCT00293423 | II | Completed |
| HSPPC-96 peptide | TMZ, radiotherapy | NCT02122822 | I | Completed |
| IDH1 peptide | - | NCT02454634 | I | Completed |
| EGFRvIII peptide | Bevacizumab | NCT01498328 | II | Completed |
| APVAC1 and APVAC2 (personalized peptide) + poly-ICLC and GM-CSF | - | NCT02149225 | I | Completed |
| Personalized neoantigen | - | NCT02287428 | I | Recruiting |
| IMA-950 (peptide comprising multiple GAAs) and polyICLC | Varlilumab (immunostimulatory anti-CD27 antibody) | NCT02924038 | I | Ongoing study |
| H3.3K27 M peptide + Td and poly-ICLC | - | NCT02960230 | I | Completed |
| AV-GBM-1 (autologous DC loaded with tumor associated antigens from a short-term cell culture of autologous tumor cells) | - | NCT03400917 | I | Completed |
| IGF-1 R/AS ODN | - | NCT02507583 | I | Completed |
| SVN53-67/M57-KLH peptide | TMZ | NCT02455557 | II | Active, not recruiting |
| EO2401 peptide | - | NCT04116658 | II | Active, not recruiting |
| pp65-shLAMP DC with GM-CSF | - | NCT02465268 | II | Completed |
| Tumor stem cell derived mRNA transfected DC | - | NCT00846456 | I/II | Completed |
| Tumor lysate-pulsed autologous DC | - | NCT01006044 | II | Completed |

**Table S9. Oncolytic virus in clinical trial for GBM treatment.**

| **Oncolytic virus** | **Clinical trial identifier** | **Origin** | **Phase** | **Status** |
| --- | --- | --- | --- | --- |
| Δ-24-RGD | NCT01582516 | Adenovirus | I/II | Completed |
| MSC-Ad5-DNX-2401 | NCT03896568 | Adenovirus | I | Recruiting |
| AloCELYVIR | NCT04758533 | Adenovirus | I/II | Recruiting |
| C134 | NCT03657576 | Herpesvirus | I | Active |
| G207 | NCT04482933 | Herpesvirus | II | Not recruiting |
| G207 | NCT00157703 | Herpesvirus | I | Completed |
| Genetically engineered HSV-1  MVR-C5252 | NCT05095441 | Herpesvirus | I | Not recruiting |
| Genetically engineered HSV-1 M032 | NCT02062827 | Herpesvirus | I | Recruiting |
| G207 infused through catheters | NCT03911388 | Herpesvirus | I | Recruiting |
| Oncolytic viral vector  rQNestin34.5v.2 | NCT03152318 | Herpesvirus | I | Recruiting |
| DNX-2401 | NCT03896568 | Adenovirus | I | Recruiting |
| DNX-2440 | NCT03714334 | Adenovirus | I | Recruiting |
| DNX-2401 | NCT00805376 | Adenovirus | I | Completed |
| DNX-2401 | NCT02197169 | Adenovirus | I | Completed |
| H-1PV | NCT01301430 | Parvovirus | I/II | Completed |
| Recombinant nonpathogenic  polio-rhinovirus chimera  (PVSRIPO) | NCT01491893 | Poliovirus | I | Active, not recruiting |
| PVSRIPO | NCT03043391 |  | I | Active, not recruiting |
| PVSRIPO | NCT02986178 |  | II | Active, not recruiting |
| REOLYSIN | NCT00528684 | Reovirus | I | Completed |
| Pelareorep | NCT02444546 | Reovirus | I | Active not recruiting |
| Combination of modified  TG6002 and 5-Flucytosine | NCT03294486 | Vaccinia  virus | I/II | Unknown |
| Combination of M032 and Pembrolizumab | NCT05084430 | Herpes virus | I/II | Active |
| Combination of NSC-CRAd-Survivin-pk7 and radiotherapy/TMZ | NCT03072134 | Adenovirus | I | Completed |
| Combination of DNX-2401 and Pembrolizumab | NCT02798406 | Adenovirus | II | Completed |

**References**

1. Taal, W., Bromberg, J. E. C. & van den Bent, M. J. Chemotherapy in glioma. *CNS Oncol.* **4**, 179–192 (2015).

2. Louis, D. N. et al. The 2021 WHO Classification of Tumors of the Central Nervous System: a summary. *Neuro Oncol*. **23**, 1231–1251 (2021).

3. Nguyen, H.-M., Guz-Montgomery, K., Lowe, D. B. & Saha, D. Pathogenetic Features and Current Management of Glioblastoma. *Cancers* **13**, 856 (2021).

4. Banu, Z. Glioblastoma Multiforme: A review of its pathogenesis and treatment. *Int. Res. J. Pharm.* **9**, 7–12 (2019).

5. Azam, Z., To, S.-S. T. & Tannous, B. A. Mesenchymal Transformation: The Rosetta Stone of Glioblastoma Pathogenesis and Therapy Resistance. *Adv. Sci.* **7**, 2002015 (2020).

6. Hanif, F., Muzaffar, K., Perveen, K., Malhi, S. M. & Simjee, S. U. Glioblastoma Multiforme: A Review of its Epidemiology and Pathogenesis through Clinical Presentation and Treatment. *Asian. Pac. J. Cancer Prev.* **18**, 3–9 (2017).

7. Ghadami, E., Jafari, M., Razipour, M., Maghsudlu, M. & Ghadami, M. Circular RNAs in glioblastoma. *Clin. Chim. Acta* **565**, 120003 (2025).

8. Wu, X. et al*.* A novel protein encoded by circular SMO RNA is essential for Hedgehog signaling activation and glioblastoma tumorigenicity. *Genome Biol.* **22**, 33 (2021).

9. Salami, R., Salami, M., Mafi, A., Vakili, O. & Asemi, Z. Circular RNAs and glioblastoma multiforme: focus on molecular mechanisms. *Cell Commun. Signal.* **20**, 13 (2022).

10. Li, D., Li, L., Chen, X., Yang, W. & Cao, Y. Circular RNA SERPINE2 promotes development of glioblastoma by regulating the miR-361-3p/miR-324-5p/BCL2 signaling pathway. *Mol. Ther. Oncolytics* **22**, 483–494 (2021).

11. Wang, Q. et al. Circular RNA circ_0001588 sponges miR-211-5p to facilitate the progression of glioblastoma via up-regulating YY1 expression. *J. Gene Med.* **23**, e3371 (2021).

12. Chen, B. et al. Circular RNA circLGMN facilitates glioblastoma progression by targeting miR-127-3p/LGMN axis. *Cancer Lett.* **522**, 225–237 (2021).

13. Tirpe, A. et al. The Glioblastoma CircularRNAome. *Int. J. Mol. Sci.* **24**, 14545 (2023).

14. Wei, Y. et al. EIF4A3-induced circular RNA ASAP1 promotes tumorigenesis and temozolomide resistance of glioblastoma via NRAS/MEK1/ERK1-2 signaling. *Neuro Oncol.* **23**, 611–624 (2021).

15. Qian, L., Guan, J., Wu, Y. & Wang, Q. Upregulated circular RNA circ_0074027 promotes glioblastoma cell growth and invasion by regulating miR-518a-5p/IL17RD signaling pathway. *Biochem. Biophys. Res. Commun.* **510**, 515–519 (2019).

16. Wang, T. et al. Blocking hsa_circ_0006168 suppresses cell proliferation and motility of human glioblastoma cells by regulating hsa_circ_0006168/miR-628-5p/IGF1R ceRNA axis. *Cell Cycle* **20**, 1181–1194 (2021).

17. Zhu, F., Cheng, C., Qin, H., Wang, H. & Yu, H. A novel circular RNA circENTPD7 contributes to glioblastoma progression by targeting ROS1. *Cancer Cell Int.* **20**, 118 (2020).

18. Tang, C., He, X., Jia, L. & Zhang, X. Circular RNAs in glioma: Molecular functions and pathological implications. *Non-coding RNA Res.* **9**, 105–115 (2024).

19. Guo, X. & Piao, H. Research Progress of circRNAs in Glioblastoma. *Front. Cell Dev. Biol.* **9**, 791892 (2021).

20. Liu, L. et al. Circular RNA circNF1 siRNA Silencing Inhibits Glioblastoma Cell Proliferation by Promoting the Maturation of miR-340. *Front. Neurol.* **12**, 658076 (2021).

21. Lv, T. et al. Circ-EPB41L5 regulates the host gene EPB41L5 via sponging miR-19a to repress glioblastoma tumorigenesis. *Aging* **12**, 318–339 (2020).

22. Yang, Y. et al. Novel Role of FBXW7 Circular RNA in Repressing Glioma Tumorigenesis. *J. Natl. Cancer Inst.* **110**, 304–315 (2018).

23. Li, P. et al. Circular RNA Sequencing Reveals Serum Exosome Circular RNA Panel for High-Grade Astrocytoma Diagnosis. *Clin. Chem.* **68**, 332–343 (2022).

24. Geng, X. et al. Exosomal circWDR62 promotes temozolomide resistance and malignant progression through regulation of the miR-370-3p/MGMT axis in glioma. *Cell Death Dis.* **13**, 1–15 (2022).

25. Wang, R. et al. CircNT5E Acts as a Sponge of miR-422a to Promote Glioblastoma Tumorigenesis. *Cancer Res.* **78**, 4812–4825 (2018).

26. Jin, P. et al. CircRNA circHIPK3 serves as a prognostic marker to promote glioma progression by regulating miR-654/IGF2BP3 signaling. *Biochem. Biophys. Res. Commun.* **503**, 1570–1574 (2018).

27. Qiao, J., Liu, M., Tian, Q. & Liu, X. Microarray analysis of circRNAs expression profile in gliomas reveals that circ_0037655 could promote glioma progression by regulating miR-214/PI3K signaling. *Life Sci.* **245**, 117363 (2020).

28. He, Q. et al. circ-SHKBP1 Regulates the Angiogenesis of U87 Glioma-Exposed Endothelial Cells through miR-544a/FOXP1 and miR-379/FOXP2 Pathways. *Mol. Ther. Nucleic Acids* **10**, 331–348 (2018).

29. Mutalifu, N. et al. Circ_0000215 Increases the Expression of CXCR2 and Promoted the Progression of Glioma Cells by Sponging miR-495-3p. *Technol. Cancer Res. Treat.* **19**, 1533033820957026 (2020).

30. Chen, Z. & Duan, X. hsa_circ_0000177-miR-638-FZD7-Wnt Signaling Cascade Contributes to the Malignant Behaviors in Glioma. *DNA Cell Biol.* **37**, 791–797 (2018).

31. Zheng, J. et al. TTBK2 circular RNA promotes glioma malignancy by regulating miR-217/HNF1β/Derlin-1 pathway. *J. Hematol. Oncol.* **10**, 52 (2017).

32. Wu, Z. et al. Hsa_circ_0043278 functions as competitive endogenous RNA to enhance glioblastoma multiforme progression by sponging miR-638. *Aging* **12**, 21114–21128 (2020).

33. Zheng, K. et al. CircRNA PIP5K1A promotes the progression of glioma through upregulation of the TCF12/PI3K/AKT pathway by sponging miR-515-5p. *Cancer Cell Int.* **21**, 27 (2021).

34. Wang, B., Li, B. & Si, T. Knockdown of circ0082374 inhibits cell viability, migration, invasion and glycolysis in glioma cells by miR-326/SIRT1. *Brain Res.* **1748**, 147108 (2020).

35. Lv, X., Wang, M., Qiang, J. & Guo, S. Circular RNA circ-PITX1 promotes the progression of glioblastoma by acting as a competing endogenous RNA to regulate miR-379-5p/MAP3K2 axis. *Eur. J. Pharmacol.* **863**, 172643 (2019).

36. Lu, Y. et al. circ_0001730 promotes proliferation and invasion via the miR-326/Wnt7B axis in glioma cells. *Epigenomics* **11**, 1335–1352 (2019).

37. Jiang, Y. et al. The U2AF2 /circRNA ARF1/miR-342–3p/ISL2 feedback loop regulates angiogenesis in glioma stem cells. *J. Exp. Clin. Cancer Res.* **39**, 182 (2020).

38. He, J. et al. Circular RNA MAPK4 (circ-MAPK4) inhibits cell apoptosis via MAPK signaling pathway by sponging miR-125a-3p in gliomas. *Mol. Cancer* **19**, 17 (2020).

39. Liu, X. et al. SRSF10 inhibits biogenesis of circ-ATXN1 to regulate glioma angiogenesis via miR-526b-3p/MMP2 pathway. *J. Exp. Clin. Cancer Res.* **39**, 121 (2020).

40. Li, G. et al. A novel circular RNA, hsa_circ_0046701, promotes carcinogenesis by increasing the expression of miR-142-3p target ITGB8 in glioma. *Biochem. Biophys. Res. Commun.* **498**, 254–261 (2018).

41. Zhou, J. et al. CircularRNA circPARP4 promotes glioblastoma progression through sponging miR-125a-5p and regulating FUT4. *Am. J. Cancer Res.* **11**, 138–156 (2021).

42. Wang, J., Li, T. & Wang, B. Circ-UBAP2 functions as sponges of miR-1205 and miR-382 to promote glioma progression by modulating STC1 expression. *Cancer Med.* **10**, 1815–1828 (2021).

43. Roh, J., Im, M., Kang, J., Youn, B. & Kim, W. Long non-coding RNA in glioma: novel genetic players in temozolomide resistance. *Animal Cells Syst.* **27**, 19–28 (2023).

44. Zhou, J. et al. Circular RNA hsa_circ_0008344 regulates glioblastoma cell proliferation, migration, invasion, and apoptosis. *J. Clin. Lab. Anal.* **32**, e22454 (2018).

45. Barbagallo, D. et al. CircSMARCA5 Inhibits Migration of Glioblastoma Multiforme Cells by Regulating a Molecular Axis Involving Splicing Factors SRSF1/SRSF3/PTB. *Int. J. Mol. Sci.* **19**, 480 (2018).

46. Xia, X. et al. A novel tumor suppressor protein encoded by circular AKT3 RNA inhibits glioblastoma tumorigenicity by competing with active phosphoinositide-dependent Kinase-1. *Mol. Cancer* **18**, 131 (2019).

47. Sun, Y., Jing, Y. & Zhang, Y. Serum lncRNA-ANRIL and SOX9 expression levels in glioma patients and their relationship with poor prognosis. *World J. Surg. Oncol.* **19**, 287 (2021).

48. Shen, J. et al. Serum HOTAIR and GAS5 levels as predictors of survival in patients with glioblastoma. *Mol. Carcinog.* **57**, 137–141 (2018).

49. Tan, S. K. et al. Serum long noncoding RNA HOTAIR as a novel diagnostic and prognostic biomarker in glioblastoma multiforme. *Mol. Cancer* **17**, 74 (2018).

50. Wang, X. et al. Serum-derived extracellular vesicles facilitate temozolomide resistance in glioblastoma through a HOTAIR-dependent mechanism. *Cell Death Dis.* **13**, 344 (2022).

51. Amer, R. G. et al. Prognostic utility of lncRNAs (LINC00565 and LINC00641) as molecular markers in glioblastoma multiforme (GBM). *J. Neurooncol.* **158**, 435–444 (2022).

52. Ho, K.-H., Shih, C.-M., Liu, A.-J. & Chen, K.-C. Hypoxia-inducible lncRNA MIR210HG interacting with OCT1 is involved in glioblastoma multiforme malignancy. *Cancer Sci.* **113**, 540–552 (2022).

53. Xie, J. et al. LncRNA SAMMSON overexpression distinguished glioblastoma patients from patients with diffuse neurosarcoidosis. *Neuroreport* **30**, 817–821 (2019).

54. Ma, C.-C. et al. Long non-coding RNA ATB promotes glioma malignancy by negatively regulating miR-200a. *J. Exp. Clin. Cancer Res.* **35**, 90 (2016).

55. Deguchi, S. et al. Oncogenic effects of evolutionarily conserved noncoding RNA ECONEXIN on gliomagenesis. *Oncogene* **36**, 4629–4640 (2017).

56. Zhang, W. et al. Long noncoding RNA FTX is upregulated in gliomas and promotes proliferation and invasion of glioma cells by negatively regulating miR-342-3p. *Lab. Invest.* **97**, 447–457 (2017).

57. Wu, X. et al. Blocking MIR155HG/miR-155 axis inhibits mesenchymal transition in glioma. *Neuro Oncol.* **19**, 1195–1205 (2017).

58. Wu, X. et al. The Involvement of Long Non-Coding RNAs in Glioma: From Early Detection to Immunotherapy. *Front. Immunol.* **13**, 897754 (2022).

59. Katsushima, K. et al. Targeting the Notch-regulated non-coding RNA TUG1 for glioma treatment. *Nat. Commun.* **7**, 13616 (2016).

60. Wei, C. et al. LncRNA HOXA11-AS promotes glioma malignant phenotypes and reduces its sensitivity to ROS via Tpl2-MEK1/2-ERK1/2 pathway. *Cell Death Dis.* **13**, 1–15 (2022).

61. Wang, Z.-H. et al. Long non-coding RNA CCAT1 promotes glioma cell proliferation via inhibiting microRNA-410. *Biochem. Biophys. Res. Commun.* **480**, 715–720 (2016).

62. Mousavi, S. M. et al. Non-coding RNAs and glioblastoma: Insight into their roles in metastasis. *Mol. Ther. Oncolytics* **24**, 262–287 (2022).

63. Fu, Z. et al. Malat1 activates autophagy and promotes cell proliferation by sponging miR-101 and upregulating STMN1, RAB5A and ATG4D expression in glioma. *Biochem. Biophys. Res. Commun.* **492**, 480–486 (2017).

64. Ma, J. et al. Knockdown of long non-coding RNA MALAT1 increases the blood-tumor barrier permeability by up-regulating miR-140. *Biochim. Biophys. Acta* **1859**, 324–338 (2016).

65. Xue, W. et al. PVT1 regulates the malignant behaviors of human glioma cells by targeting miR-190a-5p and miR-488-3p. *Biochim. Biophys. Acta Mol. Basis Dis.* **1864**, 1783–1794 (2018).

66. Sun, Y. et al. Long Noncoding RNA UCA1 Targets miR-122 to Promote Proliferation, Migration, and Invasion of Glioma Cells. *Oncol. Res.* **26**, 103–110 (2018).

67. Zhao, W., Sun, C. & Cui, Z. A long noncoding RNA UCA1 promotes proliferation and predicts poor prognosis in glioma. *Clin. Transl. Oncol.* **19**, 735–741 (2017).

68. He, Z. et al. The lncRNA UCA1 interacts with miR-182 to modulate glioma proliferation and migration by targeting iASPP. *Arch. Biochem. Biophys.* **623–624**, 1–8 (2017).

69. Yao, Y. et al. Knockdown of long non-coding RNA XIST exerts tumor-suppressive functions in human glioblastoma stem cells by up-regulating miR-152. *Cancer Lett.* **359**, 75–86 (2015).

70. Cheng, Z. et al. Long Non-coding RNA XIST Promotes Glioma Tumorigenicity and Angiogenesis by Acting as a Molecular Sponge of miR-429. *J. Cancer* **8**, 4106–4116 (2017).

71. Yu, H. et al. Knockdown of long non-coding RNA XIST increases blood-tumor barrier permeability and inhibits glioma angiogenesis by targeting miR-137. *Oncogenesis* **6**, e303 (2017).

72. Zhao, H. et al. The lncRNA H19 interacts with miR-140 to modulate glioma growth by targeting iASPP. *Arch. Biochem. Biophys.* **610**, 1–7 (2016).

73. Jia, P. et al. Long non-coding RNA H19 regulates glioma angiogenesis and the biological behavior of glioma-associated endothelial cells by inhibiting microRNA-29a. *Cancer Lett.* **381**, 359–369 (2016).

74. Guo, J. et al. Long non-coding RNA NEAT1 regulates permeability of the blood-tumor barrier via miR-181d-5p-mediated expression changes in ZO-1, occludin, and claudin-5. *Biochim. Biophys. Acta Mol. Basis Dis.* **1863**, 2240–2254 (2017).

75. Zhou, K. et al. Knockdown of long non-coding RNA NEAT1 inhibits glioma cell migration and invasion via modulation of SOX2 targeted by miR-132. *Mol. Cancer* **17**, 105 (2018).

76. Zhu, D. et al. A promising new cancer marker: Long noncoding RNA EGFR-AS1. *Front. Oncol.* **13**, 1130472 (2023).

77. Wang, S., Hui, Y., Li, X. & Jia, Q. Silencing of lncRNA CCDC26 Restrains the Growth and Migration of Glioma Cells In Vitro and In Vivo via Targeting miR-203. *Oncol. Res.* **26**, 1143–1154 (2018).

78. Cui, Y., Yi, L., Zhao, J.-Z. & Jiang, Y.-G. Long Noncoding RNA HOXA11-AS Functions as miRNA Sponge to Promote the Glioma Tumorigenesis Through Targeting miR-140-5p. *DNA Cell Biol.* **36**, 822–828 (2017).

79. Stackhouse, C. T., Gillespie, G. Y. & Willey, C. D. Exploring the Roles of lncRNAs in GBM Pathophysiology and Their Therapeutic Potential. *Cells* **9**, 2369 (2020).

80. Moradi, F., Mohajerani, F. & Sadeghizadeh, M. CCAT2 knockdown inhibits cell growth, and migration and promotes apoptosis through regulating the hsa-mir-145-5p/AKT3/mTOR axis in tamoxifen-resistant MCF7 cells. *Life Sci.* **311**, 121183 (2022).

81. Zhang, H., Wei, D.-L., Wan, L., Yan, S.-F. & Sun, Y.-H. Highly expressed lncRNA CCND2-AS1 promotes glioma cell proliferation through Wnt/β-catenin signaling. *Biochem. Biophys. Res. Commun.* **482**, 1219–1225 (2017).

82. Yadav, B. et al. LncRNAs associated with glioblastoma: From transcriptional noise to novel regulators with a promising role in therapeutics. *Mol. Ther. Nucleic Acids* **24**, 728–742 (2021).

83. Hu, G.-W. et al. Knockdown of linc-OIP5 inhibits proliferation and migration of glioma cells through down-regulation of YAP-NOTCH signaling pathway. *Gene* **610**, 24–31 (2017).

84. He, A., He, S., Li, X. & Zhou, L. ZFAS1: A novel vital oncogenic lncRNA in multiple human cancers. *Cell Prolif.* **52**, e12513 (2018).

85. Lv, Q.-L. et al*.* A Long Noncoding RNA ZEB1-AS1 Promotes Tumorigenesis and Predicts Poor Prognosis in Glioma. *Int. J. Mol. Sci.* **17**, 1431 (2016).

86. Kiang, K. M.-Y. et al*.* CRNDE Expression Positively Correlates with EGFR Activation and Modulates Glioma Cell Growth. *Target. Oncol.* **12**, 353–363 (2017).

87. Wang, Y. et al. CRNDE, a long-noncoding RNA, promotes glioma cell growth and invasion through mTOR signaling. *Cancer Lett.* **367**, 122–128 (2015).

88. Pastori, C. et al. The Bromodomain protein BRD4 controls HOTAIR, a long noncoding RNA essential for glioblastoma proliferation. *Proc. Natl. Acad. Sci.* **112**, 8326–8331 (2015).

89. Yin, T., Wu, J., Hu, Y., Zhang, M. & He, J. Long non-coding RNA HULC stimulates the epithelial-mesenchymal transition process and vasculogenic mimicry in human glioblastoma. *Cancer Med.* **10**, 5270–5282 (2021).

90. Xiang, J. et al. Silencing of Long Non-Coding RNA MALAT1 Promotes Apoptosis of Glioma Cells. *J. Korean Med. Sci.* **31**, 688–694 (2016).

91. Li, J., Ji, X & Wang, H. Targeting Long Noncoding RNA HMMR-AS1 Suppresses and Radiosensitizes Glioblastoma. *Neoplasia* **20**, 456-466 (2018).

92. Hazra, R., Debnath, R. & Tuppad, A. Glioblastoma stem cell long non-coding RNAs: therapeutic perspectives and opportunities. *Front. Genet.* **15**, 1416772 (2024).

93. Ho, K.-H., Shih, C.-M., Liu, A.-J. & Chen K.-C. Hypoxia-inducible lncRNA MIR210HG interacting with OCT1 is involved in glioblastoma multiforme malignancy. *Cancer Sci.* **113**, 540-552 (2022).

94. Pokorná, M., Černá, M., Boussios, S., Ovsepian, S. V. & O’Leary, V. B. lncRNA Biomarkers of Glioblastoma Multiforme. *Biomedicines* **12**, 932 (2024).

95. Bagheri-Mohammadi, S., Karamivandishi, A., Mahdavi, S. A. & Siahposht-Khachaki, A. New sights on long non-coding RNAs in glioblastoma: A review of molecular mechanism. *Heliyon* **10**, e39744 (2024).

96. Peng, Z., Liu, C. & Wu, M. New insights into long noncoding RNAs and their roles in glioma. *Mol. Cancer* **17**, 61 (2018).

97. Chen, W. et al. LncRNA HOXA-AS3 promotes the malignancy of glioblastoma through regulating miR-455-5p/USP3 axis. *J. Cell. Mol. Med.* **24**, 11755–11767 (2020).

98. He, C., Jiang, B., Ma, J. & Li, Q. Aberrant NEAT1 expression is associated with clinical outcome in high grade glioma patients. *APMIS* **124**, 169–174 (2016).

99. Hashemi, M. et al. Shedding light on function of long non-coding RNAs (lncRNAs) in glioblastoma. *Non-coding RNA Res.* **9**, 508–522 (2024).

100. Wang, P. et al. Long non-coding RNA CASC2 suppresses malignancy in human gliomas by miR-21. *Cell Signal.* **27**, 275–282 (2015).

101. Cao, S. et al. Tumor-suppressive function of long noncoding RNA MALAT1 in glioma cells by suppressing miR-155 expression and activating FBXW7 function. *Am. J. Cancer Res.* **6**, 2561–2574 (2016).

102. Qin, N., Tong, G.-F., Sun, L.-W. & Xu, X.-L. Long Noncoding RNA MEG3 Suppresses Glioma Cell Proliferation, Migration, and Invasion by Acting as a Competing Endogenous RNA of miR-19a. *Oncol. Res.* **25**, 1471–1478 (2017).

103. Shang, C., Guo, Y., Hong, Y. & Xue, Y.-X. Long Non-coding RNA TUSC7, a Target of miR-23b, Plays Tumor-Suppressing Roles in Human Gliomas. *Front. Cell. Neurosci.* **10**, 235 (2016).

104. Li, J., An, G., Zhang, M. & Ma, Q. Long non-coding RNA TUG1 acts as a miR-26a sponge in human glioma cells. *Biochem. Biophys. Res. Commun.* **477**, 743–748 (2016).

105. Wang, R. et al. Long noncoding RNA CASC2 predicts the prognosis of glioma patients and functions as a suppressor for gliomas by suppressing Wnt/β-catenin signaling pathway. *Neuropsychiatr Dis. Treat.* **13**, 1805–1813 (2017).

106. Chen, J. et al. Lnc-H19-derived protein shapes the immunosuppressive microenvironment of glioblastoma. *Cell Rep. Med.* **5**, 101806 (2024).

107. Xu, L.-M. et al. Over-expression of the long non-coding RNA HOTTIP inhibits glioma cell growth by BRE. *J. Exp. Clin. Cancer Res.* **35**, 162 (2016).

108. Wang, A., Meng, M., Zhao, X. & Kong, L. Long non-coding RNA ENST00462717 suppresses the proliferation, survival, and migration by inhibiting MDM2/MAPK pathway in glioma. *Biochem. Biophys. Res. Commun.* **485**, 513–521 (2017).

109. Han, Y. et al. Tumor-suppressive function of long noncoding RNA MALAT1 in glioma cells by downregulation of MMP2 and inactivation of ERK/MAPK signaling. *Cell Death Dis.* **7**, e2123 (2016).

110. Sharma, R. K., Calderon, C. & Vivas-Mejia, P. E. Targeting Non-coding RNA for Glioblastoma Therapy: The Challenge of Overcomes the Blood-Brain Barrier. *Front. Med. Technol.* **3**, 678593 (2021).

111. Li, J., Zhang, M., An, G. & Ma, Q. LncRNA TUG1 acts as a tumor suppressor in human glioma by promoting cell apoptosis. *Exp. Biol. Med.* **241**, 644–649 (2016).

112. Bassot, A. et al. Identification of a miRNA multi-targeting therapeutic strategy in glioblastoma. *Cell Death Dis.* **14**, 1–16 (2023).

113. Shi, L. et al. MiR-21 protected human glioblastoma U87MG cells from chemotherapeutic drug temozolomide induced apoptosis by decreasing Bax/Bcl-2 ratio and caspase-3 activity. *Brain Res.* **1352**, 255–264 (2010).

114. Akers, J. C. et al. miRNA contents of cerebrospinal fluid extracellular vesicles in glioblastoma patients. *J. Neurooncol.* **123**, 205–216 (2015).

115. Yang, C. H. et al. MicroRNA-21 Promotes Glioblastoma Tumorigenesis by Down-regulating Insulin-like Growth Factor-binding Protein-3 (IGFBP3). *J. Biol. Chem.* **289**, 25079–25087 (2014).

116. Gabriely, G. et al. MicroRNA 21 promotes glioma invasion by targeting matrix metalloproteinase regulators. *Mol. Cell. Biol.* **28**, 5369–5380 (2008).

117. Aloizou, A.-M. et al. The role of MiRNA-21 in gliomas: Hope for a novel therapeutic intervention? *Toxicol. Rep.* **7**, 1514–1530 (2020).

118. Shaikh, M. A. J. et al. Unraveling the impact of miR-21 on apoptosis regulation in glioblastoma. *Pathol. Res. Pract.* **254**, 155121 (2024).

119. Guo, X. et al. Immunosuppressive effects of hypoxia-induced glioma exosomes through myeloid-derived suppressor cells via the miR-10a/Rora and miR-21/Pten Pathways. *Oncogene* **37**, 4239–4259 (2018).

120. Zhang, J. et al. miR-221/222 promote malignant progression of glioma through activation of the Akt pathway. *Int. J. Oncol.* **36**, 913–920 (2010).

121. Yang, J.-K. et al. Exosomal miR-221 targets DNM3 to induce tumor progression and temozolomide resistance in glioma. *J. Neurooncol.* **131**, 255–265 (2017).

122. Malik, N. et al. Protumorigenic role of the atypical cadherin FAT1 by the suppression of PDCD10 via RelA/miR221-3p/222-3p axis in glioblastoma. *Mol. Carcinog.* **62**, 1817–1831 (2023).

123. Ahmed, S. P., Castresana, J. S. & Shahi, M. H. Glioblastoma and MiRNAs. *Cancers* **13**, 1581 (2021).

124. Peng, G. et al. miR-25 promotes glioblastoma cell proliferation and invasion by directly targeting NEFL. *Mol. Cell. Biochem.* **409**, 103–111 (2015).

125. Suh, S.-S. et al. MicroRNAs/TP53 feedback circuitry in glioblastoma multiforme. *Proc. Natl. Acad, Sci.* **109**, 5316–5321 (2012).

126. Makowska, M., Smolarz, B. & Romanowicz, H. microRNAs (miRNAs) in Glioblastoma Multiforme (GBM)—Recent Literature Review. *Int. J. Mol. Sci.* **24**, 3521 (2023).

127. Afshar, R. M., Mollaei, H. R., Shokrizadeh, M. & Iranpour, M. Evaluation Expression of Microrna-93 and Integrin Β8 in Different Types of Glioma Tumors. *Asian Pac. J. Cancer Prev.* **18**, 603–608 (2017).

128. Fang, L. et al. MicroRNA miR-93 promotes tumor growth and angiogenesis by targeting integrin-β8. *Oncogene* **30**, 806–821 (2011).

129. Chen, G., Chen, Z. & Zhao, H. MicroRNA-155-3p promotes glioma progression and temozolomide resistance by targeting Six1. *J. Cell. Mol. Med.* **24**, 5363–5374 (2020).

130. Bao, Z. et al. Exosomal miR-155-5p derived from glioma stem-like cells promotes mesenchymal transition via targeting ACOT12. *Cell Death Dis.* **13**, 725 (2022).

131. Huang, W. et al. The miR-26a/AP-2α/Nanog signaling axis mediates stem cell self-renewal and temozolomide resistance in glioma. *Theranostics* **9**, 5497–5516 (2019).

132. Wang, H. et al. miR-33a promotes glioma-initiating cell self-renewal via PKA and NOTCH pathways. *J. Clin. Invest.* **124**, 4489–4502 (2014).

133. Xia, W. et al. PD-L1 Inhibitor Regulates the miR-33a-5p/PTEN Signaling Pathway and Can Be Targeted to Sensitize Glioblastomas to Radiation. *Front. Oncol.* **10**, 821 (2020).

134. Zhang, D. et al. mir-300 promotes self-renewal and inhibits the differentiation of glioma stem-like cells. *J. Mol. Neurosci.* **53**, 637–644 (2014).

135. Kouri, F. M., Ritner, C. & Stegh, A. H. miRNA-182 and the regulation of the glioblastoma phenotype - toward miRNA-based precision therapeutics. *Cell Cycle* **14**, 3794–3800 (2015).

136. Song, L. et al. TGF-β induces miR-182 to sustain NF-κB activation in glioma subsets. *J. Clin. Invest.* **122**, 3563–3578 (2012).

137. He, H. et al. MiR-210-3p Inhibits Proliferation and Migration of C6 Cells by Targeting Iscu. *Neurochem. Res.* **45**, 1813–1824 (2020).

138. Gabriely, G. et al. Human glioma growth is controlled by microRNA-10b. *Cancer Res.* **71**, 3563–3572 (2011).

139. Ma, C. et al. MicroRNA-10b mediates TGF-β1-regulated glioblastoma proliferation, migration and epithelial-mesenchymal transition. *Int. J. Oncol.* **50**, 1739–1748 (2017).

140. Lin, J., Teo, S., Lam, D. H., Jeyaseelan, K. & Wang, S. MicroRNA-10b pleiotropically regulates invasion, angiogenicity and apoptosis of tumor cells resembling mesenchymal subtype of glioblastoma multiforme. *Cell Death Dis.* **3**, e398 (2012).

141. Zhen, L., Li, J., Zhang, M. & Yang, K. MiR-10b decreases sensitivity of glioblastoma cells to radiation by targeting AKT. *J. Biol. Res.* **23**, 14 (2016).

142. Ananta, J. S., Paulmurugan, R. & Massoud, T. F. Tailored Nanoparticle Codelivery of antimiR-21 and antimiR-10b Augments Glioblastoma Cell Kill by Temozolomide: Toward a ‘Personalized’ Anti-microRNA Therapy. *Mol. Pharm.* **13**, 3164–3175 (2016).

143. Qian, M. et al. Exosomes derived from hypoxic glioma deliver miR-1246 and miR-10b-5p to normoxic glioma cells to promote migration and invasion. *Lab. Invest.* **101**, 612–624 (2021).

144. Chen, X. *et al.* MiR-9 promotes tumorigenesis and angiogenesis and is activated by MYC and OCT4 in human glioma. *J. Exp. Clin. Cancer Res.* **38**, 99 (2019).

145. Yin, J. et al. Exosomal transfer of miR-1238 contributes to temozolomide-resistance in glioblastoma. *EBioMedicine* **42**, 238–251 (2019).

146. Yue, X., Lan, F. & Xia, T. Hypoxic Glioma Cell-Secreted Exosomal miR-301a Activates Wnt/β-catenin Signaling and Promotes Radiation Resistance by Targeting TCEAL7. *Mol. Ther*. **27**, 1939–1949 (2019).

147. Ramakrishnan, V. et al. Radiation-induced extracellular vesicle (EV) release of miR-603 promotes IGF1-mediated stem cell state in glioblastomas. *EBioMedicine* **55**, 102736 (2020).

148. Zhao, G. et al. M2-like tumor-associated macrophages transmit exosomal miR-27b-3p and maintain glioblastoma stem-like cell properties. *Cell Death Discov.* **8**, 1–10 (2022).

149. Wang, Z.-F., Liao, F., Wu, H. & Dai, J. Glioma stem cells-derived exosomal miR-26a promotes angiogenesis of microvessel endothelial cells in glioma. *J. Exp. Clin. Cancer Res.* **38**, 201 (2019).

150. Wang, M. et al. Glioma exosomal microRNA-148a-3p promotes tumor angiogenesis through activating the EGFR/MAPK signaling pathway via inhibiting ERRFI1. *Cancer Cell Int.* **20**, 518 (2020).

151. Cai, Q., Zhu, A. & Gong, L. Exosomes of glioma cells deliver miR-148a to promote proliferation and metastasis of glioblastoma via targeting CADM1. *Bull. Cancer* **105**, 643–651 (2018).

152. Mafi, A. et al. Recent insights into the microRNA-dependent modulation of gliomas from pathogenesis to diagnosis and treatment. *Cell. Mol. Biol. Lett.* **27**, 65 (2022).

153. van der Vos, K. E. *et al.* Directly visualized glioblastoma-derived extracellular vesicles transfer RNA to microglia/macrophages in the brain. *Neuro Oncol.* **18**, 58–69 (2016).

154. Guo, X. et al. Glioma exosomes mediate the expansion and function of myeloid-derived suppressor cells through microRNA-29a/Hbp1 and microRNA-92a/Prkar1a pathways. *Int. J. Cancer* **144**, 3111–3126 (2019).

155. Yang, J.-K. et al. Exosomal miR-214-5p Released from Glioblastoma Cells Modulates Inflammatory Response of Microglia after Lipopolysaccharide Stimulation through Targeting CXCR5. *CNS Neurol. Disord. Drug Targets* **18**, 78–87 (2019).

156. Li, X. et al. Microglial Exosome miR-7239-3p Promotes Glioma Progression by Regulating Circadian Genes. *Neurosci. Bull.* **37**, 497–510 (2021).

157. Figueroa, J. et al. Exosomes from Glioma-Associated Mesenchymal Stem Cells Increase the Tumorigenicity of Glioma Stem-like Cells via Transfer of miR-1587. *Cancer Res.* **77**, 5808–5819 (2017).

158. Lan, F. et al. Serum exosomal miR-301a as a potential diagnostic and prognostic biomarker for human glioma. *Cell. Oncol.* **41**, 25–33 (2018).

159. Tang, S., Bertke, A. S., Patel, A., Margolis, T. P. & Krause, P. R. Herpes Simplex Virus 2 MicroRNA miR-H6 Is a Novel Latency-Associated Transcript-Associated MicroRNA, but Reduction of Its Expression Does Not Influence the Establishment of Viral Latency or the Recurrence Phenotype. *J. Virol.* **85**, 4501–4509 (2011).

160. Qi, A., Han, J., Jia, F. & Liu, C. miR-3175 and miR-134 affect proliferation, invasion and apoptosis of glioma cells through PI3K/AKT signaling pathway. *J. BUON.* **24**, 2465–2474 (2019).

161. Xue, F., Shen, R. & Chen, X. Analysis of gene profiles in glioma cells identifies potential genes, miRNAs, and target sites of migratory cells. *Tumori* **101**, 542–548 (2015).

162. Yin, D. et al. miR-34a functions as a tumor suppressor modulating EGFR in glioblastoma multiforme. *Oncogene* **32**, 1155–1163 (2013).

163. Li, W.-B. et al. MicroRNA-34a targets notch1 and inhibits cell proliferation in glioblastoma multiforme. *Cancer Biol. Ther.* **12**, 477–483 (2011).

164. Li, Y. et al. MicroRNA-34a inhibits glioblastoma growth by targeting multiple oncogenes. *Cancer Res.* **69**, 7569–7576 (2009).

165. Ma, Z. et al. WNT signaling modulates chemoresistance to temozolomide in p53-mutant glioblastoma multiforme. *Apoptosis* **27**, 80–89 (2022).

166. Banelli, B. et al. MicroRNA in Glioblastoma: An Overview. *Int. J. Genomics* **2017**, 7639084 (2017).

167. Du, P. et al. ANXA2P2/miR-9/LDHA axis regulates Warburg effect and affects glioblastoma proliferation and apoptosis. *Cell Signal.* **74**, 109718 (2020).

168. Song, Y. et al. MicroRNA-9 inhibits vasculogenic mimicry of glioma cell lines by suppressing Stathmin expression. *J. Neurooncol.* **115**, 381–390 (2013).

169. Zu, Y. et al. MiR-9 Promotes Apoptosis Via Suppressing SMC1A Expression in GBM Cell Lines. *Curr. Chem. Genom. Transl. Med.* **11**, 31–40 (2017).

170. Mahinfar, P. et al. The Role of microRNAs in Multidrug Resistance of Glioblastoma. *Cancers* **14**, 3217 (2022).

171. Ordóñez-Rubiano, E. G. et al. The potential of miRNA-based approaches in glioblastoma: An update in current advances and future perspectives. *Curr. Res. Pharmacol. Drug Discov.* **7**, 100193 (2024).

172. Yang, B., Xia, S., Ye, X., Jing, W. & Wu, B. MiR-379-5p targets microsomal glutathione transferase 1 (MGST1) to regulate human glioma in cell proliferation, migration and invasion and epithelial-mesenchymal transition (EMT). *Biochem. Biophys. Res. Commun.* **568**, 8–14 (2021).

173. Goenka, A. et al. The Role of Non-Coding RNAs in Glioma. *Biomedicines* **10**, 2031 (2022).

174. Bai, Y. et al. MiR-296-3p regulates cell growth and multi-drug resistance of human glioblastoma by targeting ether-à-go-go (EAG1). *European J. Cancer* **49**, 710–724 (2013).

175. Balandeh, E. et al. Roles of Non-coding RNAs and Angiogenesis in Glioblastoma. *Front. Cell. Dev. Biol.* **9**, 716462 (2021).

176. Huang, T. et al. A regulatory circuit of miR-125b/miR-20b and Wnt signalling controls glioblastoma phenotypes through FZD6-modulated pathways. *Nat. Commun.* **7**, 12885 (2016).

177. Wu, N. et al. MiR-125b acts as an oncogene in glioblastoma cells and inhibits cell apoptosis through p53 and p38MAPK-independent pathways. *Br. J. Cancer* **109**, 2853–2863 (2013).

178. Shi, L. et al. MicroRNA-125b-2 confers human glioblastoma stem cells resistance to temozolomide through the mitochondrial pathway of apoptosis. *Int. J. Oncol.* **40**, 119–129 (2012).

179. Zhang, G. et al. miRNA-124-3p/neuropilin-1(NRP-1) axis plays an important role in mediating glioblastoma growth and angiogenesis. *Int. J. Cancer* **143**, 635–644 (2018).

180. Luo, L., Chi, H. & Ling, J. MiR-124-3p suppresses glioma aggressiveness via targeting of Fra-2. *Pathol. Res. Pract.* **214**, 1825–1834 (2018).

181. Qiao, W. et al. miR-124 suppresses glioblastoma growth and potentiates chemosensitivity by inhibiting AURKA. *Biochem. Biophys. Res. Commun.* **486**, 43–48 (2017).

182. Cai, S. et al. miR‑124‑3p inhibits the viability and motility of glioblastoma multiforme by targeting RhoG. *Int. J. Mol. Med.* **47**, 69 (2021).

183. Møller, H. G. et al. A Systematic Review of MicroRNA in Glioblastoma Multiforme: Micro-modulators in the Mesenchymal Mode of Migration and Invasion. *Mol. Neurobiol.* **47**, 131–144 (2013).

184. She, X. et al. miR-181 subunits enhance the chemosensitivity of temozolomide by Rap1B-mediated cytoskeleton remodeling in glioblastoma cells. *Med. Oncol.* **31**, 892 (2014).

185. Yin, J. et al. MiR-181b suppress glioblastoma multiforme growth through inhibition of SP1-mediated glucose metabolism. *Cancer Cell Int.* **20**, 69 (2020).

186. Zhang, W. et al. miR-181d: a predictive glioblastoma biomarker that downregulates MGMT expression. *Neuro Oncol.* **14**, 712–719 (2012).

187. Wang, H. et al. MicroRNA-195 reverses the resistance to temozolomide through targeting cyclin E1 in glioma cells. *Anticancer Drugs* **30**, 81–88 (2019).

188. Wang, Y. et al. miR-137: A Novel Therapeutic Target for Human Glioma. *Mol. Ther. Nucleic Acids* **21**, 614–622 (2020).

189. Peng, L. et al. MicroRNA-30a suppresses self-renewal and tumorigenicity of glioma stem cells by blocking the NT5E-dependent Akt signaling pathway. *FASEB J.* **34**, 5128–5143 (2020).

190. Chao, C.-C., Kan, D., Lu, K.-S. & Chien, C.-L. The role of microRNA-30c in the self-renewal and differentiation of C6 glioma cells. *Stem Cell Res.* **14**, 211–223 (2015).

191. Chen, Q., Wang, W., Chen, S., Chen, X. & Lin, Y. miR-29a sensitizes the response of glioma cells to temozolomide by modulating the P53/MDM2 feedback loop. *Cell. Mol. Biol. Lett.* **26**, 21 (2021).

192. Shi, C. et al. miR-29a/b/c function as invasion suppressors for gliomas by targeting CDC42 and predict the prognosis of patients. *Br. J. Cancer* **117**, 1036–1047 (2017).

193. Shi, C. et al. The miR-29 family members induce glioblastoma cell apoptosis by targeting cell division cycle 42 in a p53-dependent manner. *Eur. J. Clin. Invest.* **53**, e13964 (2023).

194. Xu, H. et al*.* miR-29s inhibit the malignant behavior of U87MG glioblastoma cell line by targeting DNMT3A and 3B. *Neurosci. Lett.* **590**, 40–46 (2015).

195. Yang, Y. et al. MicroRNA-29a inhibits glioblastoma stem cells and tumor growth by regulating the PDGF pathway. *J. Neurooncol.* **145**, 23–34 (2019).

196. Li, L., Shao, M.-Y., Zou, S.-C., Xiao, Z.-F. & Chen, Z.-C. MiR-101-3p inhibits EMT to attenuate proliferation and metastasis in glioblastoma by targeting TRIM44. *J. Neurooncol*. **141**, 19–30 (2019).

197. Lin, Y. & Wu, Z. MicroRNA-128 inhibits proliferation and invasion of glioma cells by targeting COX-2. *Gene* **658**, 63–69 (2018).

198. Chiou, G.-Y. et al. Epigenetic regulation of the miR142-3p/interleukin-6 circuit in glioblastoma. *Mol. Cell* **52**, 693–706 (2013).

199. Gheidari, F., Arefian, E., Jamshidi Adegani, F., Fallah Atanaki, F. & Soleimani, M. The miR-142 Suppresses U-87 Glioblastoma Cell Growth by Targeting EGFR Oncogenic Signaling Pathway. *Iran J. Pharm. Res.* **20**, 202–212 (2021).

200. Mei, J., Bachoo, R. & Zhang, C.-L. MicroRNA-146a Inhibits Glioma Development by Targeting Notch1. *Mol. Cell. Biol.* **31**, 3584–3592 (2011).

201. Hu, H.-Q., Sun, L.-G. & Guo, W.-J. Decreased miRNA-146a in glioblastoma multiforme and regulation of cell proliferation and apoptosis by target Notch1. *Int. J. Biol. Markers* **31**, e270-275 (2016).

202. Li, Y. et al. miR-146b-5p inhibits glioma migration and invasion by targeting MMP16. *Cancer Lett.* **339**, 260–269 (2013).

203. Katakowski, M. et al. MiR-146b-5p suppresses EGFR expression and reduces in vitro migration and invasion of glioma. *Cancer Invest.*  **28**, 1024–1030 (2010).

204. Xia, H. et al. microRNA-146b inhibits glioma cell migration and invasion by targeting MMPs. *Brain Res.* **1269**, 158–165 (2009).

205. Kefas, B. et al. microRNA-7 inhibits the epidermal growth factor receptor and the Akt pathway and is down-regulated in glioblastoma. *Cancer Res.* **68**, 3566–3572 (2008).

206. Jia, B. et al. MiR-7-5p suppresses stemness and enhances temozolomide sensitivity of drug-resistant glioblastoma cells by targeting Yin Yang 1. *Exp. Cell Res.* **375**, 73–81 (2019).

207. Yu, H. et al. MiR-4524b-5p-targeting ALDH1A3 attenuates the proliferation and radioresistance of glioblastoma via PI3K/AKT/mTOR signaling. *CNS Neurosci. Ther.* **30**, e14396 (2024).

208. Chen, C. et al. Microfluidic isolation and transcriptome analysis of serum microvesicles. *Lab Chip* **10**, 505–511 (2010).

209. Cela, I., Capone, E., Trevisi, G. & Sala, G. Extracellular vesicles in glioblastoma: Biomarkers and therapeutic tools. *Semin. Cancer Biol.* **101**, 25–43 (2024).

210. Huang, K. et al. The role of PTRF/Cavin1 as a biomarker in both glioma and serum exosomes. *Theranostics* **8**, 1540–1557 (2018).

211. Cumba Garcia, L. M., Peterson, T. E., Cepeda, M. A., Johnson, A. J. & Parney, I. F. Isolation and Analysis of Plasma-Derived Exosomes in Patients With Glioma. *Front. Oncol.* **9**, 651 (2019).

212. Wang, H. et al. Evaluation of serum extracellular vesicles as noninvasive diagnostic markers of glioma. *Theranostics* **9**, 5347–5358 (2019).

213. Ricklefs, F. L. et al. Immune evasion mediated by PD-L1 on glioblastoma-derived extracellular vesicles. *Sci. Adv.* **4**, eaar2766 (2018).

214. Nikoobakht, M. et al. A systematic update to circulating extracellular vesicles proteome; transcriptome and small RNA-ome as glioma diagnostic, prognostic and treatment-response biomarkers. *Cancer Treat. Res. Commun.* **30**, 100490 (2022).

215. Chen, Y., Jin, Y. & Wu, N. Role of Tumor-Derived Extracellular Vesicles in Glioblastoma. *Cells* **10**, 512 (2021).

216. Döring, K. et al. The Diagnostic Potential of Extracellular Vesicles Derived From the Blood Plasma of Glioblastoma Patients. *In Vivo.* **38**, 2735–2739 (2024).

217. Indira Chandran, V. et al. Ultrasensitive Immunoprofiling of Plasma Extracellular Vesicles Identifies Syndecan-1 as a Potential Tool for Minimally Invasive Diagnosis of Glioma. *Clin. Cancer Res.* **25**, 3115–3127 (2019).

218. Manterola, L. et al. A small noncoding RNA signature found in exosomes of GBM patient serum as a diagnostic tool. *Neuro Oncol.* **16**, 520–527 (2014).

219. Indira Chandran, V., Gopala, S., Venkat, E. H., Kjolby, M. & Nejsum, P. Extracellular vesicles in glioblastoma: a challenge and an opportunity. *npj Precis. Onc.* **8**, 1–8 (2024).

220. Puigdelloses, M. et al. RNU6-1 in circulating exosomes differentiates GBM from non-neoplastic brain lesions and PCNSL but not from brain metastases. *Neurooncol. Adv.* **2**, vdaa010 (2020).

221. Ma, W., Zhou, Y., Liu, M., Qin, Q. & Cui, Y. Long non-coding RNA LINC00470 in serum derived exosome: a critical regulator for proliferation and autophagy in glioma cells. *Cancer Cell Int.* **21**, 149 (2021).

222. Santangelo, A. et al. A microRNA signature from serum exosomes of patients with glioma as complementary diagnostic biomarker. *J. Neurooncol.* **136**, 51–62 (2018).

223. Tan, S. K. et al. Serum long noncoding RNA HOTAIR as a novel diagnostic and prognostic biomarker in glioblastoma multiforme. *Mol. Cancer* **17**, 74 (2018).

224. Wang, X. et al. Identification of low-dose radiation-induced exosomal circ-METRN and miR-4709-3p/GRB14/PDGFRα pathway as a key regulatory mechanism in Glioblastoma progression and radioresistance: Functional validation and clinical theranostic significance. *Int. J. Biol. Sci.* **17**, 1061–1078 (2021).

225. Shao, N. et al. miR-454-3p Is an Exosomal Biomarker and Functions as a Tumor Suppressor in Glioma. *Mol. Cancer Ther.* **18**, 459–469 (2019).

226. Tzaridis, T. et al. Analysis of Serum miRNA in Glioblastoma Patients: CD44-Based Enrichment of Extracellular Vesicles Enhances Specificity for the Prognostic Signature. *Int. J. Mol. Sci.* **21**, 7211 (2020).

227. Dai, J., Jiang, Y., Hu, H., Zhang, S. & Chen, Y. Extracellular vesicles as modulators of glioblastoma progression and tumor microenvironment. *Pathol. Oncol. Res.* **30**, 1611549 (2024).

228. Manda, S. V. et al. Exosomes as a biomarker platform for detecting epidermal growth factor receptor-positive high-grade gliomas. *J. Neurosurg.* **128**, 1091–1101 (2018).

229. Figueroa, J. M. et al. Detection of wild-type EGFR amplification and EGFRvIII mutation in CSF-derived extracellular vesicles of glioblastoma patients. *Neuro Oncol.* **19**, 1494–1502 (2017).

230. Wang, M. et al. Exosomal LGALS9 in the cerebrospinal fluid of glioblastoma patients suppressed dendritic cell antigen presentation and cytotoxic T-cell immunity. *Cell Death Dis.* **11**, 896 (2020).

231. Chen, W. W. et al. BEAMing and Droplet Digital PCR Analysis of Mutant IDH1 mRNA in Glioma Patient Serum and Cerebrospinal Fluid Extracellular Vesicles. *Mol. Ther. Nucleic Acids* **2**, e109 (2013).

232. Tankov, S. & Walker, P. R. Glioma-Derived Extracellular Vesicles – Far More Than Local Mediators. *Front. Immunol.* **12**, (2021).

233. Li, J., Yuan, H., Xu, H., Zhao, H. & Xiong, N. Hypoxic Cancer-Secreted Exosomal miR-182-5p Promotes Glioblastoma Angiogenesis by Targeting Kruppel-like Factor 2 and 4. *Mol. Cancer Res.* **18**, 1218–1231 (2020).

234. Hallal, S. et al. Extracellular Vesicles from Neurosurgical Aspirates Identifies Chaperonin Containing TCP1 Subunit 6A as a Potential Glioblastoma Biomarker with Prognostic Significance. *Proteomics* **19**, e1800157 (2019).

235. Hallal, S. et al. Deep Sequencing of Small RNAs from Neurosurgical Extracellular Vesicles Substantiates miR-486-3p as a Circulating Biomarker that Distinguishes Glioblastoma from Lower-Grade Astrocytoma Patients. *Int. J. Mol. Sci.* **21**, 4954 (2020).
